# Understanding zoonotic spillover interfaces for the design of a One Health education intervention: a qualitative baseline study from rural Thailand

**DOI:** 10.1186/s42522-026-00228-1

**Published:** 2026-08-04

**Authors:** Kate Bärnighausen, Monchai Phongsiri, Kyra Lilier, Simaphon Tomklang, Theerayuth Labooth, Thanapat Chaibut, Pongsakon Jaruton, Kotthamon Chanprathad, Sarutaya Suphawatthanaphan, Hans Overgaard, Mark Donald C. Reñosa, Bianca Joyce Sornillo, Jonas Wachinger, Hussein Amin, Mohamed Shelil, Cassia Rocha Pompeu, Uliana Kachnova, Doreen Montag, Vidhya Sasitharan, Till Bärnighausen, Marina Treskova, Patcharin Lapanun

**Affiliations:** 1 Heidelberg Institute of Global Health (HIGH), Im Neuenheimer Feld 130.3, 69120 Heidelberg, Germany; 2 The Center for Research on Plurality in the Mekong Region (CERP), Faculty of Humanities and Social Sciences, Khon Kaen University, Khon Kaen, Thailand; 3 Faculty of Medicine, Khon Kaen University, Khon Kaen, Thailand; 4 Faculty of Science and Technology, Norwegian University of Life Sciences, Ås, Norway; 5 Department of Epidemiology and Biostatistics, Research Institute for Tropical Medicine, Department of Health, Muntinlupa, Philippines; 6 Wolfson Institute of Population Health, Queen Mary University of London, London, UK; 7 Interdisciplinary Centre for Scientific Computing, Heidelberg University, Heidelberg, Germany; 8 Department of Epidemiology and Global Health, Umeå University, Umeå, Sweden

**Keywords:** Community, Qualitative, Zoonotic spillover, Risk interfaces, One Health, Prevention, Intervention

## Abstract

**Background:**

Prevention of zoonotic spillover requires an understanding of how human, animal, and environmental interactions shape risk in everyday life. Efforts have focused largely on surveillance and response rather than on primary prevention informed by lived experience. In Southeast Asia—a recognised hotspot for zoonotic emergence—there is limited qualitative evidence describing how spillover risk is shaped by routine practices, environmental change, and structural constraints, and how such evidence can inform the design of One Health education interventions.

**Methods:**

We conducted a formative qualitative study in Nan Province, northern Thailand, employing shared walks, in-depth interviews, community mapping, and participant and non-participant observation. Data were analysed using reflexive thematic analysis and organised using a qualitative One Health Risk Interface Framework encompassing human–animal, human–environment, human-human, animal–animal and animal–environment interfaces.

**Results:**

Spillover risk and mitigation was produced and experienced through routine food preparation, hunting, farming, and caregiving practices. The human–animal interface includes frequent handling of wildlife and domestic animals, mixed-species environments, and informal meat processing. The human–environment interface is characterised by water scarcity, contamination, deforestation, landslides, and inadequate waste management. The human-human interface describes information and communication issues and how these problems can be addressed. The animal–animal and animal–environment interfaces reflect shifting ecologies driven by land-use change, secondary forests, and agricultural intensification, bringing wildlife, livestock, and people into closer proximity.

**Conclusion:**

Zoonotic spillover risk in rural Thailand is produced through interconnected behavioural, ecological, and structural processes that cannot be addressed through individual behaviour change alone. An education intervention will need to develop both knowledge and skills within a broader framework of conservation and acknowledgment of structural resource constraints.

**Supplementary Information:**

The online version contains supplementary material available at 10.1186/s42522-026-00228-1.

## Background

The COVID-19 pandemic demonstrated the need to understand the ecological and social origins of pandemics and to invest in primary prevention strategies [1]. Most emerging infectious diseases (EIDs) are zoonotic, arising when pathogens spill over from wildlife reservoirs into human populations through pathways shaped by environmental change, livelihoods, and social systems [2]. Spillover events of zoonotic origin are increasing by roughly 5% annually [3], reflecting accelerated anthropogenic disruption of planetary systems. If the current trend continues, spillover events could quadruple and related mortality increase twelvefold by 2050 [3]. Yet global investment remains heavily weighted toward surveillance and response, rather than proactive prevention, despite evidence that prevention is more cost-effective, equitable, and sustainable [4, 5].

The rise of zoonotic EIDs is driven by climate change [6], environmental degradation, population growth, land-use change, agricultural intensification, biodiversity loss, and socio-economic instability [2, 4]. Southeast Asia is a recognized hotspot for zoonotic spillover, as this is where many of these pressures converge [7]. While literature has advanced understanding of the biological mechanisms of spillover, much less is known about how spillover risk is produced and managed in everyday life [8]. In particular, the perspectives and everyday actions of people living in high-risk regions remain underexplored in both research and policy discussions, and are underutilized as sources upon which to inform and design interventions [8].

Effective spillover prevention requires interventions that are locally grounded, culturally resonant, and attentive to structural constraints [9]. Human-Centred Design (HCD) offers a participatory framework for developing interventions by beginning with an in-depth understanding of people’s experiences, motivations, and decision-making contexts [10]. In the empathise phase, researchers seek – often qualitatively - to understand what people do, why they do it, and how behaviours and interactions are embedded within routines, infrastructures, relationships, and place [11]. The insights generated during the empathise stage shape the content, structure, and delivery of any subsequent intervention and feed directly into the define and ideate phases, where they are synthesised into actionable problem statements and opportunity areas, informing message framing, pedagogical approaches, and the feasibility of behaviour change [11]. Across domains and disciplines, qualitative and participatory approaches have been key to the empathise stage of human centred intervention design, and have strengthened intervention relevance, community ownership, and sustainability [12–15]. However, despite the demand for qualitative approaches to understand intervention content and development, few zoonotic spillover prevention interventions have been meaningfully informed by qualitative or participatory research [5].

In this study, we used qualitative and participatory methods to develop an understanding of zoonotic spillover interfaces as conceptualised in One Health literature [3]. Through shared walks, community mapping, interviews, and observations, we documented how spillover risk and mitigation is produced within the lived, multispecies, and environmental realities of households, farms, markets, villages, and forest-edge spaces in Thailand. The baseline findings presented here constitute the empathise phase of a HCD process and provide the understanding required to co-design an educational intervention. The resulting intervention will be evaluated in a population-based cluster randomised controlled trial (cRCT). 

## Methods

### Study design

We employed a formative qualitative design using shared walks (SW), in-depth interviews (IDIs), participant and non-participant observations and community mapping (CM).

### PANDA project

PANDA is a One Health research project that investigates the interactions that drive zoonotic spillover and emerging infectious diseases. PANDA integrates epidemiological, environmental, and social science approaches to generate contextually grounded evidence for prevention and preparedness. The project synthesises existing knowledge on spillover risk and pandemic response, examines local perceptions, behaviours, and structural drivers across study sites, and analyses environmental change to better understand evolving risk landscapes. The PANDA project team will co-design and evaluate a community-tailored pandemic prevention and preparedness literacy (3PL) intervention and strengthen locally relevant, scalable strategies to reduce spillover risk and enhance resilience to future pandemics.

### Study sites

**Fig. 1:**
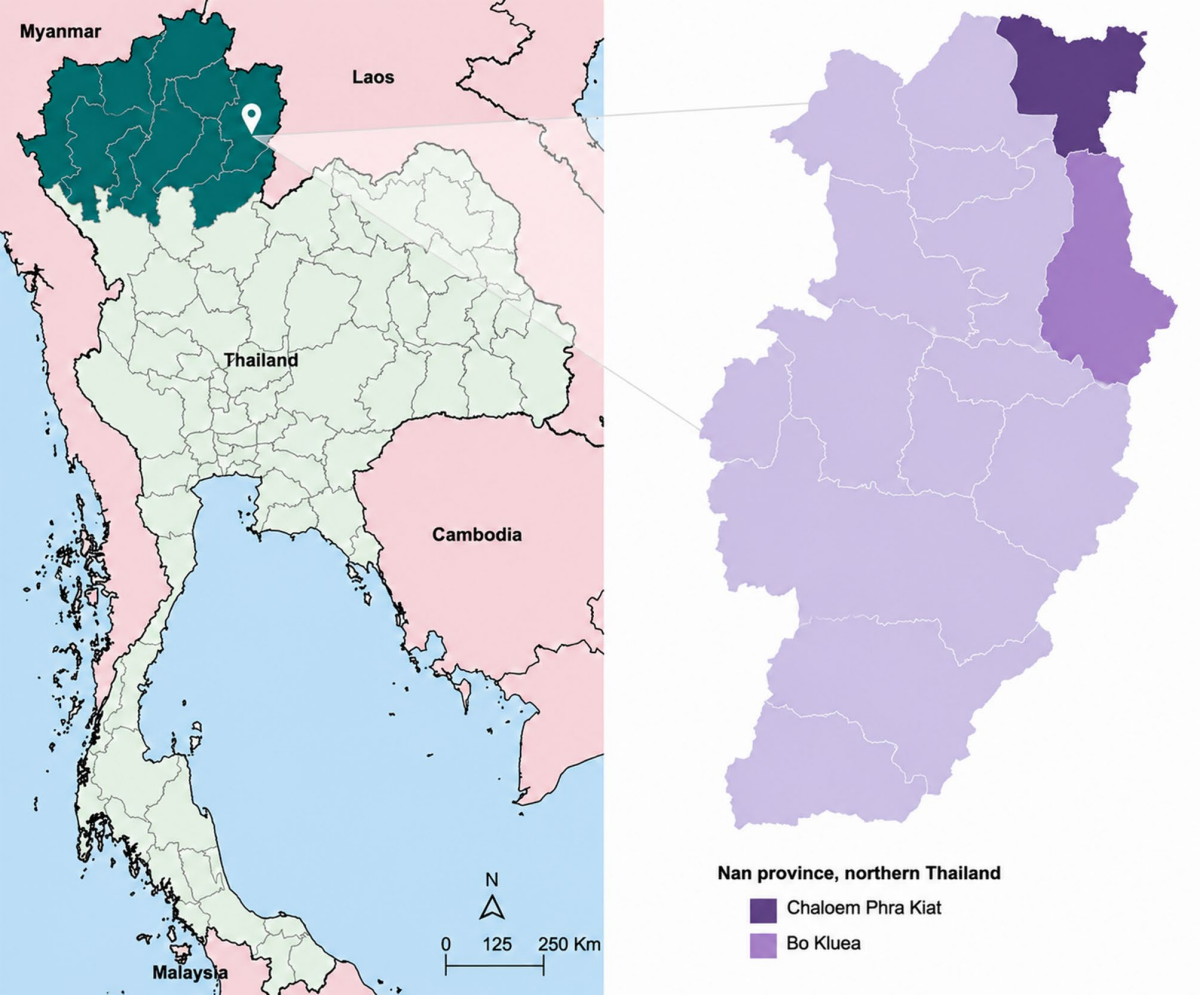
Data collection districts, Nan Province

Our study was conducted in four rural villages across two districts (Chaloem phra kiat and Bo Kluea) in the north-western province of Nan, Thailand (Fig. 1). Nan is located approximately 668 km north of Bangkok and has a population of 478,227 [16] across an area of 12,130 km^2^. Nan comprises 13 ethnic groups each with distinct languages, cultures and beliefs [17]. The landscape is characterized by a central valley dominated by agriculture and urban areas. This valley is surrounded by steep, forest-covered mountains with several protected parks. Over recent decades, Nan has experienced substantial deforestation, driven largely by agricultural expansion, particularly maize cultivation [18, 19]. The climate is characterized by a pronounced wet season from May to October, contributing for approximately 85% of the annual rainfall, followed by a cooler dry season from November to April. Mean annual temperature is 25.6 °C, with average precipitation reaching around 1,382 mm [20].

### Sampling and recruitment

We purposively selected adults (≥18 years) living in the study sites who held roles or responsibilities with relevance to zoonotic spillover risk and community-level decision-making. We selected equal representation of male and female adults. Participants were chosen to represent a range of social authority, livelihood practice, and local knowledge. This included community leaders (CLs), village health volunteers (VHVs), farmers, mothers, foresters, hunters, market vendors, cleaners, and traditional healers, including ritual practitioners. This sampling strategy was informed by representativeness, and was designed to capture the diversity of everyday practices, governance roles, and knowledge systems that shape how spillover risk is produced, interpreted, and managed at the community level.

### Preparation

Six Thai postgraduate-level research assistants (RAs) were trained in qualitative and participatory data collection methods. Training included the theoretical foundations of constructivism, the study objectives, and detailed preparation for IDIs, SWs, observations, and CM. RAs were actively engaged as co-researchers, contributing to the design, structure, and translation of all data collection tools to ensure linguistic accuracy and cultural relevance. We allocated two days for practical field training and to pilot test our data collection instruments and finalized all instruments – on the basis of our piloting – on the last day of training. Prior to data collection, the Thai research team undertook extensive community outreach and engagement, including community meetings with district and village leaders and household level discussions, to establish trust and secure local approval.

### Data collection

From the 11th to the 24th of January 2025, we collected qualitative data in Thailand. In each village, key community locations were established as our field bases, serving as points of contact for participants and the broader community. Participants were invited to attend an initial orientation session, during which the research team introduced the project and our research aims and methods. All participants received the information sheet in oral or written form and signed a consent form once RAs ensured the purpose and aims of the study were fully understood. Consent forms were either signed or inked with a thumb print. SWs were participant-led, in areas they felt were most relevant for the study. IDIs were conducted one-on-one in a private space chosen by the participant. Participant and non-participant observations were conducted at markets between 06:00–10.00, 10:00–14:00, 14:00–18:00 and 18:00–22:00 for four days. RAs conducting the observations were changed every four hours. CM was conducted in each village with key community members and facilitated by a trained moderator and supported by a notetaker. We conducted daily debriefing sessions to capture initial thoughts and insights into the data, and to refine data collection processes where it required improvement [21]. 

### Data analysis

All recordings, notes, photos and observations were translated from Thai to English using AI transcription software, and were quality checked and amended against the original audio recordings by a bilingual team at the Khon Kaen University Department of English Language. Data were analysed using a hybrid inductive and deductive reflexive thematic analysis (RTA) approach [22]. All data from IDIs, SWs, CM and observations were imported into Nvivo Pro 15. Analysis began with repeated readings of transcripts, fieldnotes, walk-route documentation, and photographed mapping outputs to achieve familiarity with the dataset [22]. We developed a set of inductive codes alongside deductive, theoretically informed codes. Our data was diverse and required extensive organization of interview transcripts, conversation notes, observational notes, photographs, and maps. Community mapping outputs were digitised and features such as water points, animal movement corridors, market zones, farms, waste sites, and forest edges were interpreted and triangulated with participants’ narrative accounts. We deliberately looked for data that could be used for intervention design and communication, and developed codes with this in mind. Following coding, data were grouped and examined to identify patterns of meaning. We developed themes that represented how spillover-related behaviours and conditions were experienced, enacted, or described in daily life. Themes were reviewed, refined, and finalised with the broader research team.

### Reflexivity and rigor

At the start of the training week, all researchers were asked to present a reflexivity statement to outline any preconceived ideas they had in relation to the research. We openly discussed thoughts and interpretations, and revisited and acknowledged them throughout data collection and analysis. Our daily debriefing sessions allowed us to review the data and compare our initial ideas in real time [21]. We embedded a week-long data analysis workshop into the analysis process where all RAs were invited to present their thoughts and ideas relating to the dataset and review and contribute to the findings we present here. We incorporated their feedback and interpretations into our guiding framework, and refined our analysis based on their reflections [23].

### Ethics

Ethics approval was obtained from Heidelberg University Ethics Commission (S-686/2024). Our ethics application at Khon Kean University was waived as deemed as not required. Our research was conducted in accordance with the Declaration of Helsinki [24].

## Results

We present findings from IDIs, SWs, CM sessions and four days of participant and non-participant observations. 36 participants (Tables 1 and 2) participated in an IDI, of which 26 also completed a SW. We present the characteristics of 16 participants from 4 CM sessions, some of whom were also interviewed and took part in a SW.

**Table 1:** Participants location 1

Characteristics	Number	%
Total	26	100
Female	16	62
Male	10	38
Age group		
18–29 Years	2	8
30–49 Years	12	46
50–69 Years	9	35
70 +	3	12
Religion		
Buddhist	24	92
Christian	0	0
Traditional	1	4
Traditional and Buddhist	1	4
Relationship Status		
Married	21	81
widowed	2	8
single	3	12
Number of children		
0	3	12
1–2	17	65
3- 5	6	23
6+	0	0
Highest Education Level		
No formal education	0	0
Primary Education	8	31
Middle School	3	12
High School Education	8	31
Tertiary Education	5	19
Vocational Training	2	8
Occupation (more than 1 possible)		
Household	3	12
Farmer	11	42
Hunter	1	4
Leader	6	23
Health Volunteer	2	8
Office Worker	1	4
Healer	2	8
Nurse	1	4
Garbage Collector	1	4
Vendor	4	15

**Table 2:** Participants location 2

Characteristics	Number	%
Total	10	100
Female	8	80
Male	2	20
Age group		
18–29 Years	2	20
30–49 Years	4	40
50–69 Years	4	40
70 +	0	0
Religion		
Buddhist	6	60
Christian	4	40
Traditional	0	0
Traditional and Buddhist	0	0
Relationship Status		
Married	8	80
widowed	1	10
single	1	10
Number of children		
0	0	0
1–2	7	70
3- 5	1	10
6+	2	20
Highest Education Level		
No formal education	3	30
Primary Education	1	10
Middle School	1	10
High School Education	4	40
Tertiary Education	0	0
Vocational Training	1	10
Occupation (more than 1 possible)		
Household	6	60
Farmer	9	90
Hunter	2	20
Health Volunteer	2	20

### Conceptual framework

**Fig. 2:**
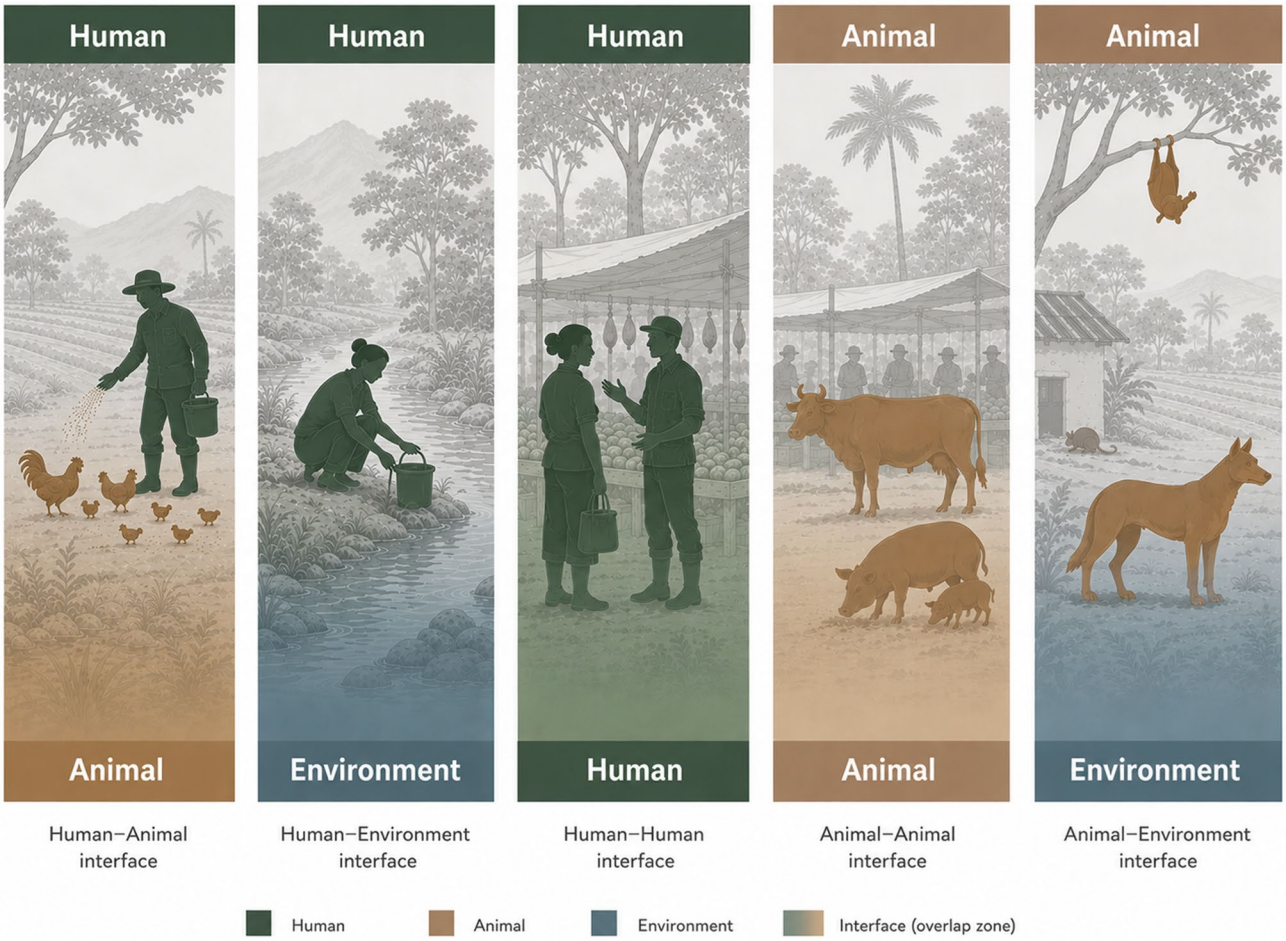
Risk interfaces

We situate our work within a One Health conceptual framework that recognises where human, animal, and environmental health are interconnected, and that zoonotic spillover occurs through interactions across these domains rather than at a single point of contact [25]. A One Health framing allows us to examine how everyday practices, livelihoods, environmental conditions, and human–animal relationships collectively shape opportunities for pathogen transmission while highlighting the broader social and ecological systems in which spillover risk is produced and experienced [25]. Within this conceptual framing, we organise and present our data using risk interfaces (Fig. 2) illustrating each with selected drawings, maps, and images that convey the context and range of practices (Supplementary Tables 3, 4, 5, 6 and 7). In One Health literature the term interface refers to a point or context in which humans, animals, and their surrounding environments interact in ways that create opportunities for infectious agents to move between hosts [26, 27]. These interactions may involve direct contact between humans and animals or indirect exposure through contaminated surfaces, materials, food, water, or other objects that can facilitate the transmission of pathogens such as viruses, bacteria, and parasites [26]. We extend this framing to present spillover risk as produced and experienced from interactions that occur at five interconnected interfaces: (1) the human–animal interface, (2) the human–environment interface, (3) the human-human interface, (4) the animal-animal interface and (5) the animal–environment interface. Interfaces 1,2,4 and 5 represent the ecological, behavioural, and socio-cultural spaces in which pathogens can be transmitted, amplified, or brought into contact with susceptible hosts. The human-human interface (3) describes where knowledge, beliefs, trust, misinformation, and behavioural norms circulate within communities. We conceptualise this human-human communication as an interface because it represents the point at which people interact with one another to interpret, negotiate, and act upon information related to spillover risk. These exchanges shape behaviours occurring at all other interfaces, influencing how people interact with animals, respond to environmental hazards, adopt preventive practices, and engage with public health interventions. We are not presenting these interfaces as measurable characteristics of pathogen contact, exposure, and ecological conditions that can be translated into variables, indicators, or risk scores. We describe them qualitatively without making assumptions of frequency or intensity of contact.

Figure 2 depicts the five interconnected interfaces. Each panel shows a distinct interaction space between humans, animals, and the environment. The coloured gradients represent the interface zone and highlight where interactions occur. Humans are shown in green, animals in orange and the environment in blue. The human–animal interface describes direct and indirect contact between people and both domestic and wild animals. Activities such as hunting, handling, slaughtering, preparing raw meat, consuming wildlife, or interacting with free-roaming domestic animals can facilitate cross-species pathogen transmission. The human–environment interface includes exposure to water, soil, vegetation, surfaces, or infrastructures that can harbour pathogens shed by animals or humans. Risk behaviours include using untreated water sources, living near unmanaged waste, or engaging in agricultural or foraging activities. We also describe environmental degradation, such as deforestation, land conversion, and extractive activities in this interface. The human-human interface describes where spillover risk and mitigation are shaped by approaches to disseminating information at the community and household level. The animal–animal interface captures the direct and indirect interactions among domestic animals and wildlife. The animal–environment interface describes how wildlife and domestic animals move through and interact with the environment. Examples include livestock grazing in wildlife habitats, shared water points for wild and domestic species, and waste sites that attract animals such as rodents and bats.

### Human–animal interface

Across villages, interactions between humans, domestic animals, wildlife, and animal products were shaped by livelihood practices, regulatory constraints, and changing ecological conditions. Participants consistently described a marked decline in encounters with large wildlife, attributing this to deforestation, land-use change, and hunting restrictions. As a result, most human–animal contact involved domestic animals and small wildlife species rather than large mammals.

On farms, contact with domestic animals was structured and spatially organised. Cattle and pigs were typically kept in farm areas away from village centres, while chickens were housed in shelters adjacent to homes. Farmers described routine handling of animals for feeding, cleaning, treatment, and occasional slaughter, with pigs and poultry slaughtered on site when required. Participants said that sick animals were usually recognised early and treated. During disease outbreaks, participants notified officials, isolated animals, vaccinated livestock (particularly chickens and cattle), and, in some cases, culled the sick animals. Dead animals were buried, while animals that died from disease were sometimes burned. Hunting and direct wildlife contact was limited to small animals such as rats, moles, squirrels, bats, and edible insects, which participants described as affordable, accessible sources of protein and, in the case of rodents, consumption also served as a form of pest control. Many participants emphasised that hunting practices were self-regulated, shaped by wildlife scarcity, legal restrictions, and COVID-19–related bans. Large wildlife was considered to be absent, with participants noting that hunting of larger species occurred primarily across the border in Laos, where meat was then sold at markets. Butchering and preparation of hunted animals occurred on farms, in fields, in forests, by rivers and in household kitchens. Meat was prepared without gloves, usually using a single cutting board, and handwashing between tasks was limited by water availability.

Within households, participants described confident and practiced approaches to assessing meat quality, and relied on colour, smell, texture, and visual inspection to judge safety. Sick animals were not eaten. Meat—both domestic and wild—was usually consumed cooked, and participants emphasised that raw consumption of wildlife had declined substantially over recent decades. A small number of dishes involving raw meat, most commonly buffalo, were prepared only at certain ceremonies. Ritual uses of animals were common, particularly involving cooked chicken; fewer rituals involved raw meat, and these were infrequent and dependent on resources and the specific event. Some participants said that dogs were vaccinated, whereas others said none were vaccinated. Participants frequently referenced established procedures for handling animal-related injuries, although understanding of appropriate wound care and rabies prophylaxis following dog bites appeared inconsistent.

Market environments were perceived to be separate from village life. Although geographically close, many participants stated that they did not buy meat from markets. Vendors were primarily from Laos, while customers often came from urban areas or other countries. The market is where we also observed meat handling: raw domestic and wild meat was displayed on tables, banana leaves, polystyrene, or floors; blood and bile were collected in small plastic bottles; and cutting occurred without gloves or cold storage. Mobile markets were a common and popular food distribution system. Vendors transported vegetables, meat, seafood, spices, and prepared foods by pickup truck or motorcycle to sell directly within villages, providing an important source of daily food for residents and playing a more central role in local food access than nearby border markets.

### Human–environment interface

Human exposure to contaminated water, surfaces, waste, and degraded environments was shaped by water scarcity, infrastructural gaps, and rapid environmental change. Participants consistently described water availability as the most pressing environmental concern, as the lack of water influenced hygiene practices, food preparation, farming, and everyday household routines.

In household and village settings, limited access to piped water meant that most participants relied on rainwater harvesting, stored water, and greywater reuse for domestic tasks such as laundry, cleaning, and sometimes crop or animal care. These constraints reduced handwashing and surface cleaning despite otherwise orderly and well-maintained domestic spaces. Food preparation areas often lacked sinks and were spatially merged with washing zones, with utensils stored in open spaces on floors or shelves. During shared walks, participants showed us drainage channels and waterways that were no longer considered safe for use, explaining that runoff now carried contaminants from nearby dump sites, including domestic wastewater, refuse, dog faeces, and agricultural residues. Waste management practices varied: some households separated waste, while others disposed of it in waste pits that were periodically burned. The accumulation of rubbish was observed in shared spaces, gutters, and informal dumps, including one located beside a school, where greywater and runoff created stagnant pools that persisted throughout the year.

On farms, human–environment interfaces were shaped by water limitations, land-use restrictions, and agricultural intensification. Water for cleaning was collected from ponds or streams, and there were visible hydrological connections between animal ponds and crop irrigation systems. Participants described mixing fermented fertilisers and animal manures close to livestock enclosures, and we observed poor drainage at animal handling points. Although chemical fertilisers and pesticides were widely used, many farmers described them as dangerous and expressed a preference for biological fertilisers or allowing land to recover naturally. However, farmers explained that such approaches were increasingly difficult to sustain due to restrictions relating to traditional cultivation practices and the requirement to plant permanent crops. Fields were regularly burned at specific times of year, with participants describing precautionary measures to limit dust pollution and prevent fires from spreading. 

Forest environments held both ecological and cultural significance. Participants explained that they enter forests to collect edible vegetables, plants, and herbs, and emphasised the presence of long-standing traditions and rituals governing land and plant use. People spoke of asking forest spirits for permission before harvesting and a reliance on inherited knowledge to identify medicinal plants. At the same time, forest exposures were shaped by the absence of hygiene infrastructure as hunting and ritual sites lacked access to water, soap, or disinfectants, meaning that contaminated hands and tools were not washed across multiple tasks. Deforestation and canopy loss were thought to accelerate changes in water flow, with participants pointing out streams that had dried, flooded unpredictably, or become seasonally unreliable. Landslides were common and worsening, and residents identified unstable slopes, the locations of recent landslides, and areas where vegetation removal had left soil exposed. As a result, some households considered their housing temporary and anticipated future relocation when land became uninhabitable. Participants also said that conservation zoning and reforestation initiatives restricted forest access, making everyday practices such as gathering forest products harder.

### Human – human interface

Our data points to the communication of information as a key component of risk in the human-human interface. Drawing on experiences from Covid, participants explained that contact between people was not an issue as lockdown and quarantine procedures were followed as directed. Rather – despite recognition of the efficient Village Health Volunteer (VHV) system - risk occurred through poor communication, misinformation and a lack of consistent messaging. Prevention of spillover would only be successful if these issues were addressed with the local context in mind. Community leaders (CLs) were viewed as gatekeepers whose engagement and endorsement of information would shape participation, credibility, and acceptance of new practices. CLs provided routine information via village loud speakers and the social media platform LINE. Although participants said this was a main source of important information, some VHVs preferred in-person house-to-house visits as not all community members were comfortable using smart phones and there was limited access to reliable internet. VHVs were considered trusted and competent providers of knowledge and services, and participants consistently referred to the progress made in tackling issues such as mosquito larve control. Participants could detail the process and procedures for prevention and treatment, including appropriate notification of illness to VHVs so preventative measures could be put in place. Participants stressed that messages must be locally relevant, grounded in familiar examples, and tailored to specific groups within the community. VHVs explained they required not only technical knowledge about zoonotic risks but also training in how to communicate these risks to diverse audiences, and how to embed sustained behaviour change within everyday routines. Communication needs described by VHVs and CLs extended across linguistic, cultural, and generational lines as communities are comprised people originating from multiple regions and ethnic backgrounds, each requiring materials in different languages, with culturally resonant cues. Age-specific messaging was thought to be important as distinct approaches were needed for children, adolescents, adults, and older people. Participants said that engaging materials such as videos, plays, and leaflets were effective modes of communication and, if supported with small useful gifts, would contrast with prior interventions that failed due to poor communication and contextual fit. The Royal Trust–initiated cement frog farm ponds were repeatedly cited as an example of a poorly designed intervention. The income-generating project proved culturally and practically unsuitable because a lack of clean water rendered the ponds non-functional, which led to the ponds being repurposed as rubbish receptacles.

### Animal-animal interface

We observed multiple forms of animal–animal interaction. On farms, there was mixed-species contact between pigs, chickens, and cattle where animals were housed in adjacent or interconnected pens, and dogs, cats, and poultry roamed freely around pig and cow enclosures. Participants described incursions by wild species—including rats, civets, bats, and occasionally elephants—into areas where domestic and farm animals were kept. In market settings, we did not see many animal–animal interactions but observed small numbers of live chickens kept near stalls, free-roaming dogs moving between vendors, and cattle passing through or standing adjacent to market perimeters. Storage containers holding insects, eels, frogs, and other live species were positioned near each other.

### Animal–environment interface

We observed interactions between domestic animals, wildlife, and land. In households, free-roaming dogs and poultry moved between indoor cooking areas and outdoor spaces. Participants reared buffalos, which were left to freely roam in large areas in or close to the forest. On farms, ongoing land-use change—including crop transitions, burning, and diversification into perennial fruit trees—altered local ecologies in ways that displaced and attracted wildlife. Participants described rising populations of crop-associated rats and insects, alongside increased feeding by bats, civets and squirrels on fruit trees. Chicken manure was applied to fields and hydro connections between fishponds and crop plots enabled animal waste to enter irrigation pathways. Forests in the study area were considered ‘secondary’ meaning a forest that was planted after the original forest was destroyed. These forests were less dense, with fewer species, smaller trees and shifting species compositions. National-park zoning and restricted access concentrated human activity at forest edges, bringing domestic animals, and wildlife into closer proximity. Smaller species (e.g., barking deer, mole rats, squirrels, bats, edible insects) were more commonly encountered than larger mammals, reflecting – what was described as - extensive deforestation that reduced the amount of larger forest animals, and brought our participants into contact with a narrower but more frequent set of wildlife species. In villages, free-roaming animals scavenged at open waste sites and dumps. Burial of dead animals was common practice and was not considered a risky behaviour. Market settings intensified animal–environment interactions by concentrating raw meat, wildlife products, waste, and food preparation within a confined area with inadequate sanitation and drainage.

## Discussion

We used qualitative and participatory methods to examine zoonotic spillover risk across human–animal, human–environment, human-human, animal-animal and animal–environment interfaces in rural communities in Thailand. We observed routine cross-species contact through wildlife handling, domestic animal management, and food preparation practices. Environmental exposures were shaped not only by household hygiene constraints but also by accelerating deforestation, contaminated water systems, and landslides. At the animal–environment interface, shifting ecologies, declining forest density, and changes in crop composition altered wildlife presence and brought people, livestock, and wild animals into closer proximity. Communication was a clear cross-cutting issue, with participants emphasising the need for locally relevant messaging, multilingual communication materials, engagement and endorsement of education approaches with trusted CLs, and training for VHVs.

Our findings resonate with broader conceptualisations of One Health and EcoHealth regarding where and how spillover risk is produced [28, 29]. Rather than locating risk primarily within individual behaviours or specific contact within interfaces, our data suggest that spillover is formed through interconnected socio-ecological systems shaped by livelihood demands, environmental change, infrastructure, governance decisions, and unequal access to resources [30]. We see a clear challenge to existing attempts to separate ecological and social processes, as ecosystem integrity, biodiversity, livelihoods, and human wellbeing are clearly mutually constitutive [29, 31]. The interconnection is evident in our work and supported by a body of research demonstrating that risk of zoonotic spillover often happens in routine, everyday practices involving wildlife contact, domestic animal husbandry, and food preparation [32, 33]. Evidence from Southeast Asia describes where smallholder farming systems, mixed-species environments, and traditional hunting practices are important in shaping cross-species interactions [34, 35], and other qualitative and ethnographic research from Laos, Cambodia, and Indonesia describes how hunting, butchering, and consumption of wildlife are informed by perceptions of safety [36], superior nutritional quality [36], livelihood needs [37], food security [38], and local ecological knowledge [39], rather than a cultural preference [40]. Multispecies ethnographies in Thailand and Vietnam have further illustrated how humans and animals co-produce risk through shared landscapes, seasonal routines, and everyday interactions that are rarely captured by biomedical framings alone [41, 42].

Research on live animal markets and peri-domestic environments similarly shows how mixed-species contact, environmental contamination, and informal food systems create sustained opportunities for pathogen exchange [43, 44]. Qualitative work from sub-Saharan Africa and Latin America highlights how households navigate risk within structural constraints, balancing disease prevention with livelihood needs, water scarcity, and competing priorities [45, 46]. These findings resonate with our observations of tidy homes, careful cooking practices, and risk mitigation that occurs despite infrastructural limitations. Our work echos studies demonstrating that land-use change, deforestation, and agricultural intensification shape both wildlife behaviour and local experiences of environmental instability [47–49]. For example, research has documented how farmers in shifting agroecological zones interpret changes in wildlife presence, water availability, and land degradation, and how such environmental changes, rather than isolated behaviours, create the conditions under which spillover becomes more likely [50, 51]. Moreover, changes in wildlife presence as a result of shifting agriculture has increased hunting and consumption of rats [40] alongside influencing biodiversity and ecosystem functioning [52].

While many identified interface behaviours appear high-risk from a biomedical perspective, our findings highlight that these practices are shaped primarily by interconnected socio-ecological systems [31] including economic constraints, affordability, and locally rational strategies, rather than by neglect. For many households, catching rats, bats, crickets, or other wildlife was described not primarily as a cultural tradition but as an affordable, accessible source of protein. Participants also framed rodent hunting as pest control, thereby serving two simultaneous functions. Ceremonial uses of animals were varied, and when they did occur at large events such as a wedding, participants used farm animals and cooked the meat thoroughly. Smaller crop-based rituals did involve wild animals and the consumption of raw meat but were conducted by a small group of farmers seasonally, suggesting that ritual practices do not drive wildlife contact when compared to economic necessity.

Many participants expressed a strong positive regard for nature, emphasising that forest animals were “clean” because they were not exposed to pesticides, plastics, or fumes. Therefore, we caution against deficit framings that portray communities as careless, and instead highlight how people actively navigate and minimise risk within structural limits. However, knowledge gaps remain, for example, wound care following dog bites is unclear, and transmission pathways across animals, meat products, forest interactions, water use and food preparation are varied. Participants would benefit from knowledge in relation to these domains.

Our findings also highlight how rapid shifts in agricultural production—often driven by external market forces or policy directives—were not accompanied by the infrastructure needed to support them. Expanding fruit orchards and high-value crops offered short-term income security but increased cascading vulnerabilities in regions with limited water access and unstable landscapes. Participants repeatedly noted that new crops required far more irrigation than local systems could sustainably provide, accelerating groundwater depletion, increasing reliance on contaminated surface water, and competition between farming and household needs. Similarly, deforestation linked to agricultural expansion, road building, and zoning decisions further altered hydrological patterns and increased landslide risk. Even though such upstream environmental processes lie beyond the scope of our educational intervention, it is important to acknowledge them because they structure the exposure interfaces that households must navigate daily. Rather than discouraging crop cultivation—which would be neither feasible nor ethical—we suggest promoting awareness of sustainable, locally adapted agricultural practices, such as selecting less water-intensive crop varieties, improving rainwater harvesting, and integrating agroecological approaches that reduce reliance on pesticides and chemical fertilisers. Including this kind of content in community education may help reinforce One Health principles that highlight how environmental stewardship supports human and animal wellbeing. In doing so, the intervention can empower communities with knowledge, without placing responsibility for large-scale infrastructural problems onto those least able to address them.

### Application to intervention design: content, method, and messenger

Within the HCD process, an important output of the empathise phase is a series of design questions that translate participants experiences into actionable design challenges and questions [11]. For example, observations of mixed-species contact, limited access to water, strong trust in village health volunteers, and the cultural value placed on wild meat highlighted that effective interventions would need to move beyond information provision alone [53]. The data pointed towards three interconnected design questions: What knowledge and skills should be prioritised? How should they be communicated within resource-constrained settings? And who is best positioned to communicate them? These questions - alongside evidence from behavioural and implementation science - informed the development of intervention concepts during the ideation phase and provide the organising structure for the recommendations presented below.

### Content 

Based on the information described across the interfaces, we recommend that the intervention content should focus on both knowledge and skills. Across infectious disease prevention, water, sanitation and hygiene (WASH), antimicrobial resistance, and zoonotic disease programmes, interventions have been most successful when educational content is paired with opportunities for skill development, social reinforcement, and environmental conditions that enable behaviour change [54]. The effectiveness of health education is constrained by structural barriers such as poverty, because limited access to clean water, inadequate infrastructure, unequal power relations, and broader political and environmental conditions hamper the application of aquired knowledge and skills [55, 56].

Educational messages could emphasise safe food-handling practices, including separation of chopping boards and knives for raw and cooked foods, handwashing, and avoiding the mixing of utensils across animal and food preparation tasks. These messages could be supported with practical skill-building demonstration sessions. However, such messages must be adapted to the reality that many households lack sufficient water, making extensive washing impractical. Rather than prescribing idealised hygiene practices, messages should focus on low-water or water-efficient strategies, such as wiping surfaces, using antibacterial gels or sprays, designating tools for raw meat, and safely storing utensils. 

Given the normalisation of mixed-species contact, educational content could also address risks associated with roaming domestic animals—particularly dogs and poultry entering food-preparation areas—while offering practical strategies that do not require costly infrastructure (e.g. designating animal-free zones during cooking). Knowledge regarding the vaccination of domestic and farm animals and appropriate wound cleaning, care and onward clinic referral (if bitten or scratched) should form a core component of the intervention content. Furthermore, as wild meat is widely perceived as healthy, desirable, and culturally valued, effective messaging should therefore build on, rather than confront, these beliefs by acknowledging cultural practices while highlighting specific points in handling and preparation where exposure risk is highest. Importantly, many upstream drivers of spillover risk—including water scarcity, deforestation, unstable landscapes, and contaminated drainage systems—are structural and not amenable to individual behaviour change. It would be neither ethical nor effective to ask households to wash more frequently when water is unavailable, nor to expect communities to alter land-use patterns driven by government zoning, commercial investment, or external economic pressures. Instead, educational messages can focus on strengthening understanding of upstream processes, emphasising how environmental degradation affects human and animal health without placing responsibility for structural conditions on local communities. Messages may highlight concepts such as the role of healthy forests in supporting clean and stable water systems, the relationship between soil health and food safety, and the ways environmental instability increases disease risk.

### Communication methods

The effectiveness of these messages will depend on the modes of communication through which they are delivered. Studies evaluating community-based health promotion programmes have found that opportunities to observe, practise, and discuss new behaviours improve both comprehension and uptake, particularly in settings where literacy levels vary [56]. Similarly, implementation research highlights the importance of tailoring communication materials to local cultural contexts and existing modes of information sharing to maximise acceptability and engagement [57]. As many households are resource-poor and VHVs operate with limited tools, educational sessions could embed practical artefacts or small gifts—such as chopping boards, separate knives for vegetables and meat, wound-cleaning supplies, and basic animal-handling tools (e.g. gloves or masks)—to reinforce learning through use. Leaving participants with functional objects may allow for messages to be enacted in daily routines rather than remaining abstract. As participants consistently emphasised the value of multiple, engaging and accessible formats, including videos, plays, demonstrations, and small visual materials we recommend providing consistent content across as many of these formats as possible.

### Messengers

Our findings indicate that any intervention is most likely to be effective when co-designed with CLs and VHVs and tailored to linguistic and cultural diversity. Community health workers have been shown to be effective when they share social, linguistic, and cultural characteristics with the populations they serve, enabling them to translate technical information into locally meaningful guidance [58]. The effectiveness is because trust, social proximity, and sustained engagement are important mechanisms through which health information is accepted and acted upon [59]. We recommend that CLs are mobilised to initiate engagement, legitimise intervention activities, and communicate when, where, and how educational sessions will take place. As VHVs were identified as trusted and competent providers of household health information, we feel they are well positioned to deliver One Health education alongside existing household visits. Training for VHVs could extend beyond technical knowledge of zoonotic risks to include skills in communicating with diverse audiences and embedding behaviour change within established routines. This aligns with evidence that VHV community-based programmes require adequate training, support, autonomy, and resources if they are to avoid overburdening volunteers and achieve sustained impact [60, 61]. Our data suggests that VHVs would value the additional training and responsibility, and would not perceive it as a burden.

## Limitations

Our findings highlight important limitations to educational approaches for spillover prevention. While interventions can support knowledge development, risk awareness, and the adoption of feasible risk-reduction practices, many of the conditions shaping spillover risk in these communities are structural and extend beyond the control of individual households. Water scarcity, deforestation, unstable landscapes, inadequate waste management systems, and limited infrastructure constrain the extent to which recommended behaviours can be implemented or have impact, regardless of knowledge or motivation. Consequently, even contextually adapted strategies, such as low-water hygiene practices, may be insufficient where access to basic resources is severely restricted. Educational interventions should therefore be viewed as one component of a broader One Health response rather than a standalone solution. The most reasonable contribution of an educational intervention may lie in helping communities recognise and navigate existing risks, while simultaneously generating locally grounded evidence that can inform advocacy, policy development, and investment in the environmental and infrastructural conditions necessary to support sustained risk reduction.

We acknowledge that a key limitation is that these were one-off visits and therefore provide a cross section of practices, perceptions, and environmental conditions across 3 weeks. Spillover risk is dynamic and may vary substantially across seasons and in response to changing ecological, economic, and social conditions. A more sustained presence within communities may have captured temporal variations that were not visible during the study period and provided deeper insight into how spillover risks occur, intensify and are managed over time. We recommend that future research adopts longitudinal and more-than-human approaches to better understand the seasonal and evolving nature of human–animal–environment interactions.

## Conclusion

We provide a descriptive, place-based examination of zoonotic spillover interfaces in rural Thailand. Our findings show that communities navigate these interfaces within complex structural, ecological, and economic constraints—including water scarcity, deforestation, unstable landscapes, mixed-species environments, and shifting agricultural systems—that directly influence how people live, farm, hunt, cook, and care for animals. Households actively implement contextually rational strategies to protect health, even in the absence of formal infrastructure. Many practices that might appear as risky from a biomedical perspective were locally understood as necessary, resource-efficient, or protective. While human-centred design provided a valuable framework for understanding community experiences and co-creating locally relevant intervention strategies, future research may benefit from engaging with emerging more-than-human design approaches that explicitly centre the perspectives, agencies, and interdependencies of animals, ecosystems, and environmental processes alongside those of people. The intervention resulting from our findings could place emphasis on protective and preventative skills across the interfaces – using artefacts and tools as methods to reinforce understanding - but also build understanding of broader upstream drivers of risk that can be mitigated by conservation of wildlife and natural ecosystems.

## Electronic supplementary material

Below is the link to the electronic supplementary material.

Supplementary Material 1: Table 3. Human-Animal interface, Table 4. Human-Environment interface, Table 5. Human-Communication interface, Table 6. Animal-Environment interface, Table 7. Animal- Animal interface.

## Data Availability

The dataset supporting the conclusions of this article is available upon request.
