# Supplementary material for: Understanding zoonotic spillover interfaces for the design of a One Health education intervention: a qualitative baseline study from rural Thailand
Source: One Health Outlook. 2026 Aug 4;8:38. doi: 10.1186/s42522-026-00228-1 (PMC13440113; doi:10.1186/s42522-026-00228-1)
Supplement: Supplementary file 1 [file 42522_2026_228_MOESM1_ESM.pdf]

Table 3. Human-Animal interface

Code	Quote	Image	SW Note
Disappearing wildlife	People hunted a lot, and the natural habitat is disappearing. There used to be so many fish too. Some younger people even used to throw a single homemade bomb and come back with sacks full of fish. That's no longer possible. (Man, 56)<br><br>It's kind of contrary to what we've always been told, right? That if there's more forest, animals should return. But from what I've seen with my own eyes, it's not like that at all. The trees are growing, sure—but it's eerily quiet. There's no sound, no trace of animals. In some places, it's completely silent, like there's no life there at all. […] Almost all of them [have disappeared]. Back then, if you walked a bit into the forest, you'd see signs of wild boar or monkeys. Now, they're all gone. I think they can't survive because the trees we planted are fast-growing species that just block sunlight. But they don't produce food or nesting places for the animals. These new forests look beautiful—they're full of trees—but there's no biodiversity. No food, no shelter. It doesn't	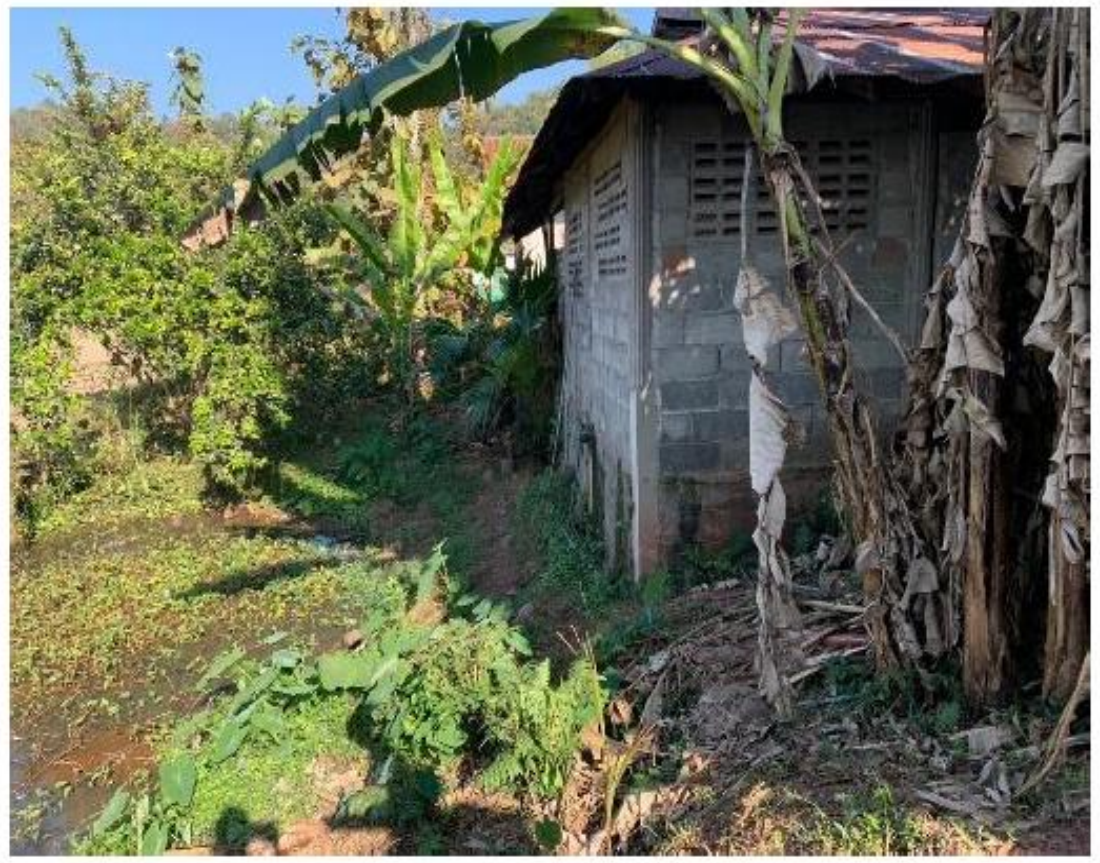 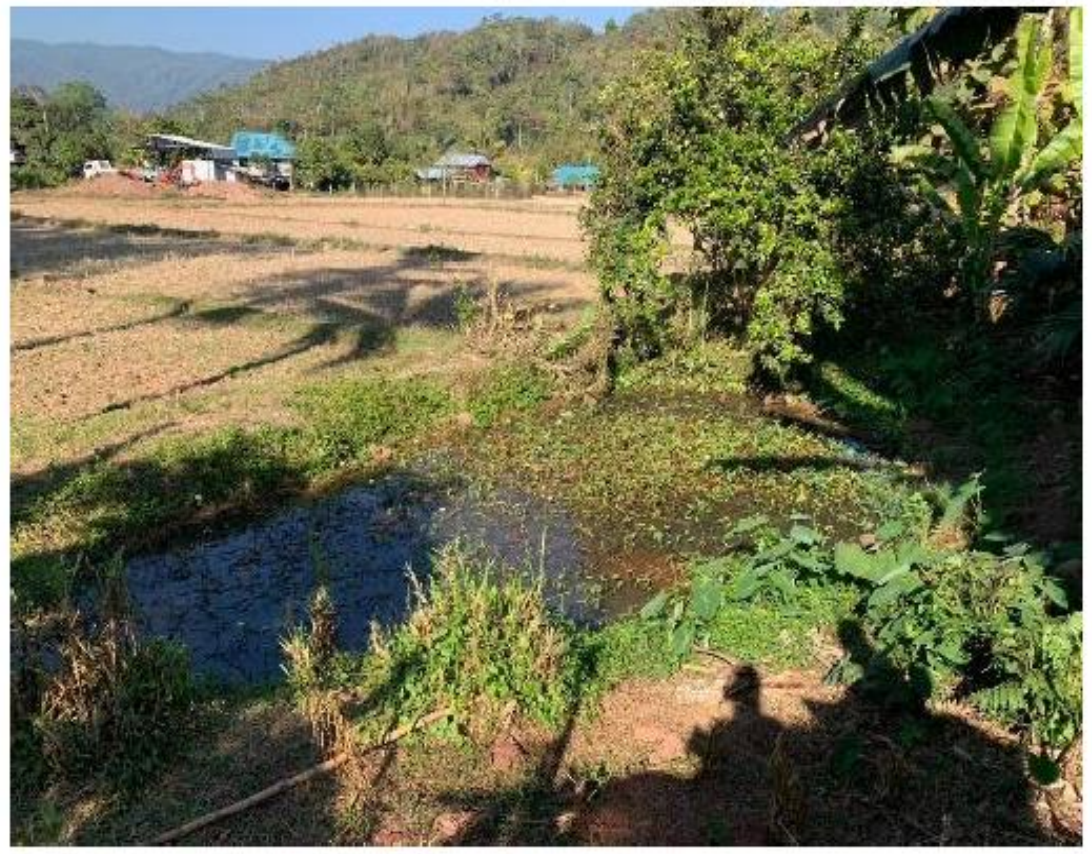<br><br>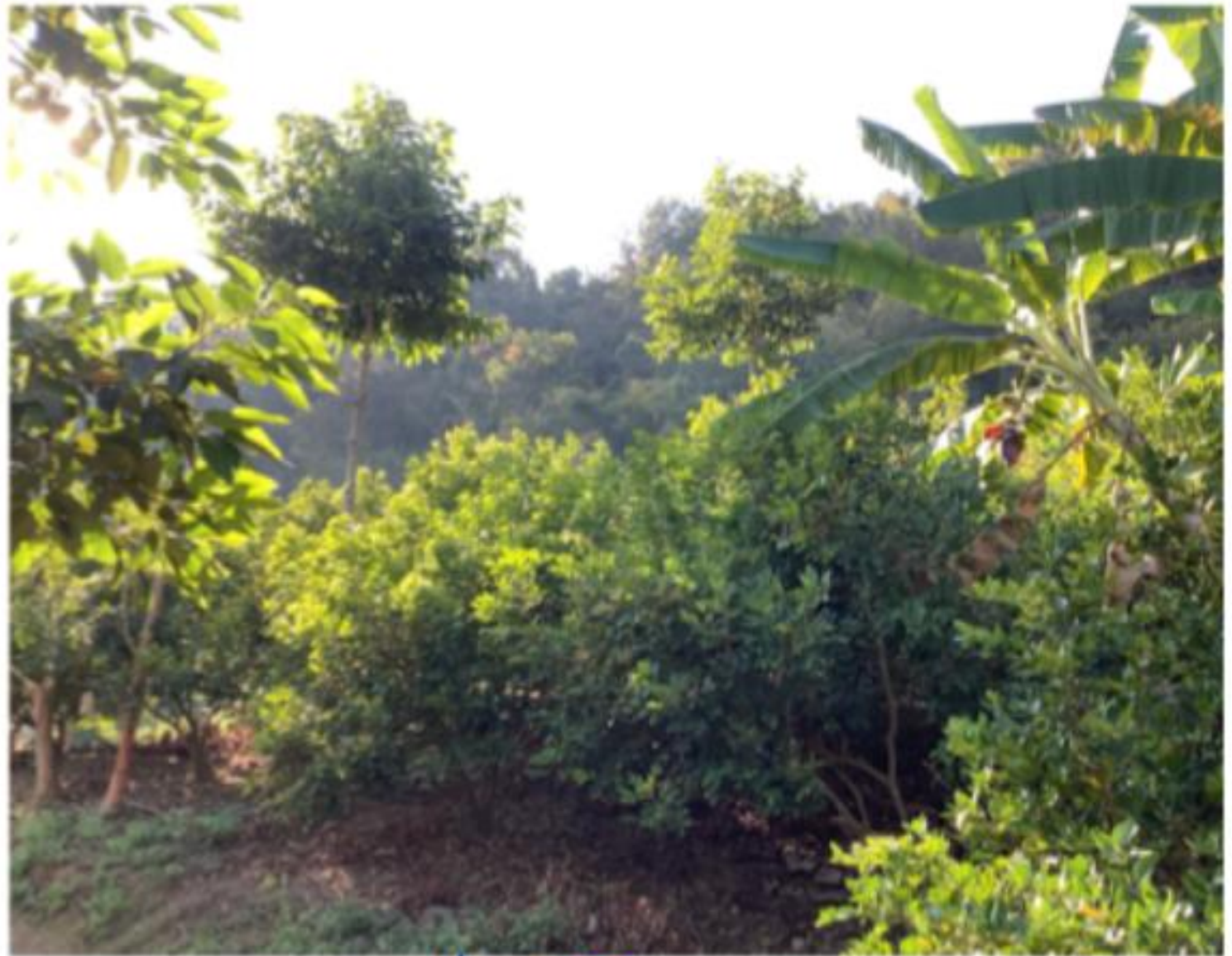<br>ต้นมะนาวที่ปลูกไว้	Behind the toilet, there is a fish pond. The interviewee participated in the fish farming project, but no fish is given yet. So, basically there is no fish in the pond.

	support wildlife at all. In the end, we're seeing strange animals move in instead—sometimes snakes or creatures we've never seen before. Sometimes they come to forage in our gardens or even inside our homes, so we have to be more cautious. In the past, we had forests and animals, and we could coexist. Now, we're trying to conserve, but it's having the opposite effect. […] Sometimes we act with good intentions but don't realize we're disrupting important parts of nature that we don't fully understand. (Man, 56)		
Interaction with wildlife in farms	Bats come out at night. If there are ripe bananas, they'll come. But I don't put up nets. […] I just let them eat. If a banana tree is ripe, they can have it. It's almost like I'm raising them. I don't know where they sleep at night, but they come to my orchard in large numbers. They use banana leaves. They'll bite and break off pieces of the leaves and hang upside down beneath. […] I don't [eat them]. (Man, 41)<br><br>Civets, they come to eat bananas quite often. You can see their bite marks on banana trees. Moles are almost gone around here. I've been setting traps for them. […] they eat		

	my plants' roots, sometimes they burrow into the roots and then move to another. I've lost many rambutan trees to them in the past. So, I've been using traps to catch them, when they died, I cook them for food. (Man, 41)	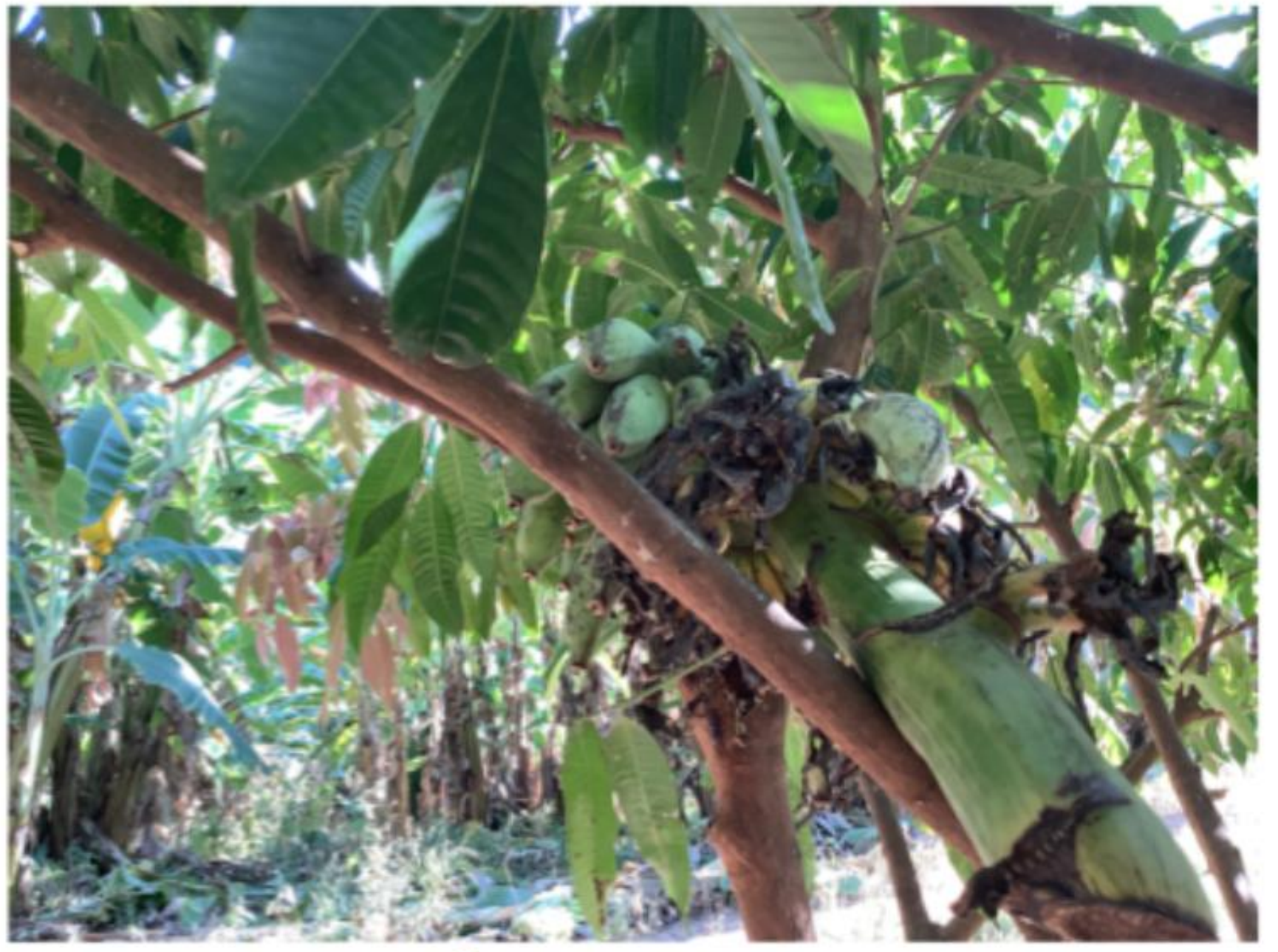<br>ค้างคาวกินกล้วย (รอบ)<br>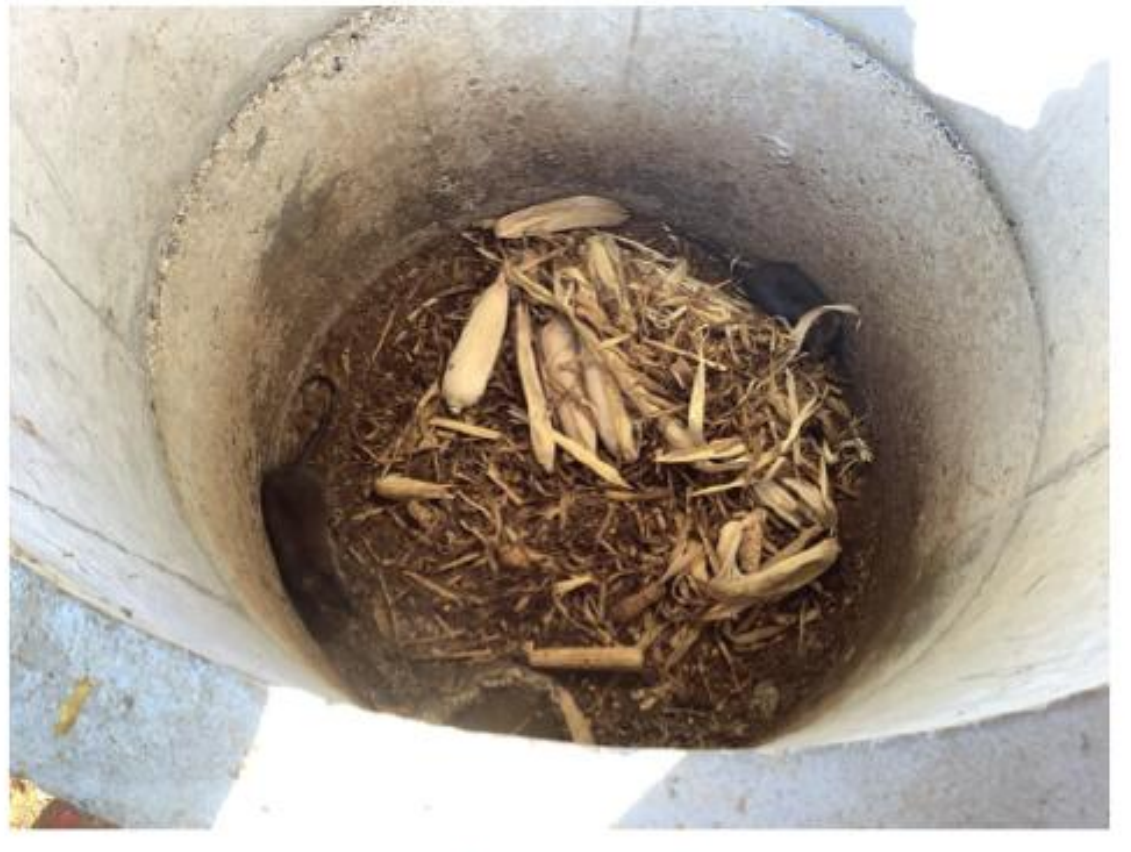<br>หนูเลี้ยงจากญาติสี่ เพชรบูรณ์<br>หนูที่ล่าได้สมัยนี้มีน้อย	

		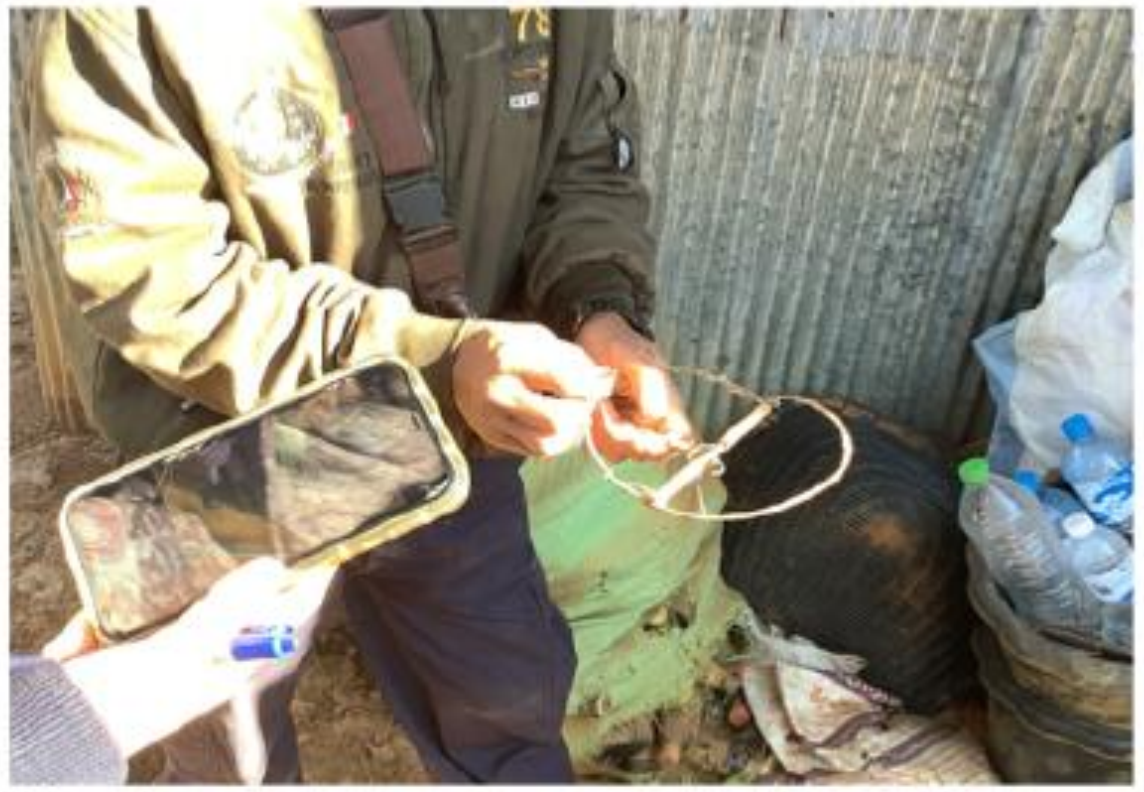<br>rat trap bought from Laos (border market)	
Hunting wildife	The past four or five years—I don't eat it, I don't accept it [wildmeat]. These days, if someone goes hunting, it's a crime. And not just for the hunter—even the person who eats it is at fault. I don't think village leaders eat that stuff anymore. Nobody wants to talk about it openly now. But in some villages, they still do. Some leaders still get offered wild meat. […] it's because these animals are starting to go extinct. They're disappearing fast. If we let hunting continue, our future generations won't have anything left to see. Nothing at all. We've already committed the wrongdoing, and the next generation will have to bear the consequences—just like the	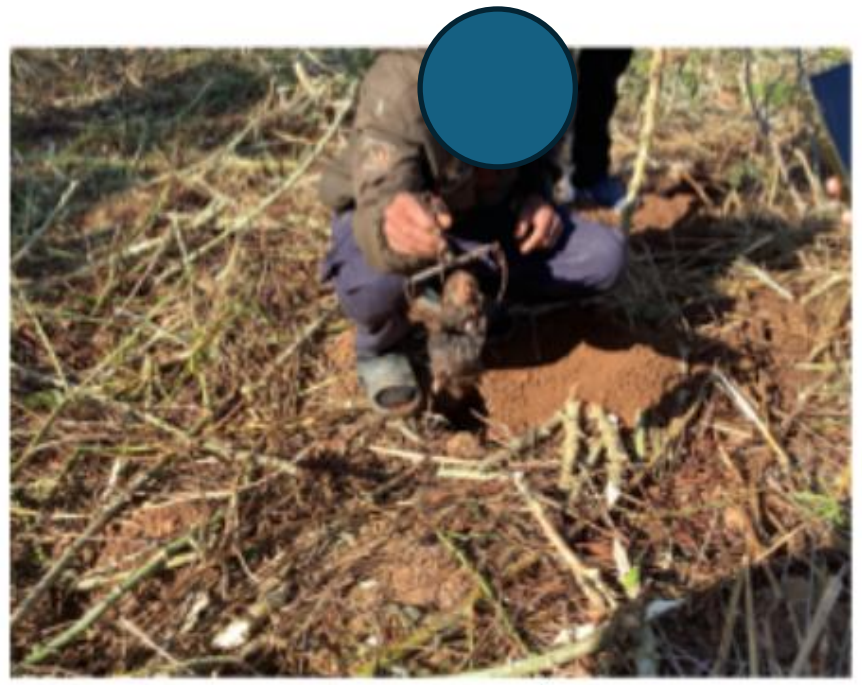<br>ตุ่นได้จากการดักจับที่ไร่มันสำปะหลัง ตัวที่ 1<br>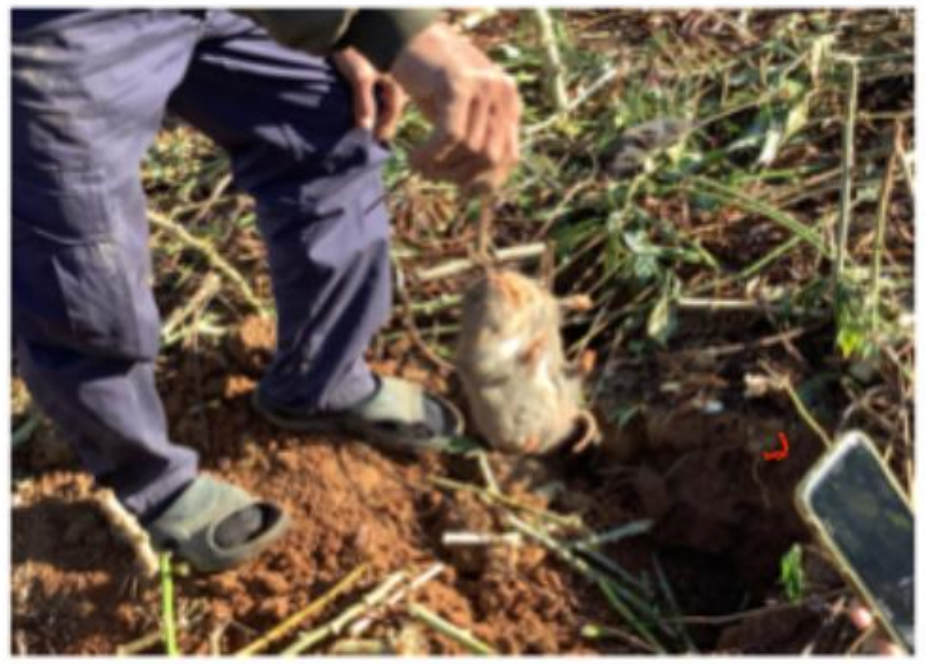<br>ตุ่นตัวที่ 2 ที่ดักได้<br>Activate Wind Go to Settings to a	Most of the time, Grandma's other son, who lives in the house next door, is the one who goes hunting birds and setting traps for rats. If he catches any, he brings them to Grandma to cook. (Women, 75)

	King once taught us. Once I became a community leader, I started educating others. Sometimes there are conflicts between people's beliefs and the actual practices. (Man, 56)<br><br>There are legal restrictions. The law says you can't hunt wildlife, which is true. But in reality, if people don't hunt, they don't have anything to eat. So some still go anyway. […] If they get the chance, they go. If not, they don't—it's as simple as that. Most people know who's doing what and just share things around quietly. […] They [leaders] know. Even before I became a community leader, I used to share meat with them. And after I became a leader, we still shared it just the same. […] Everyone knows, but no one reports it, and no one takes serious action. (Man, 56)<br><br>[I hunt] around the farmland, in the fields and orchards. […] I keep setting traps in different places. […] I cook them [right] right away. Sometimes, if there's any leftover, I grill it. After grilling, I put it in the fridge. (Man, 55, Hunter)	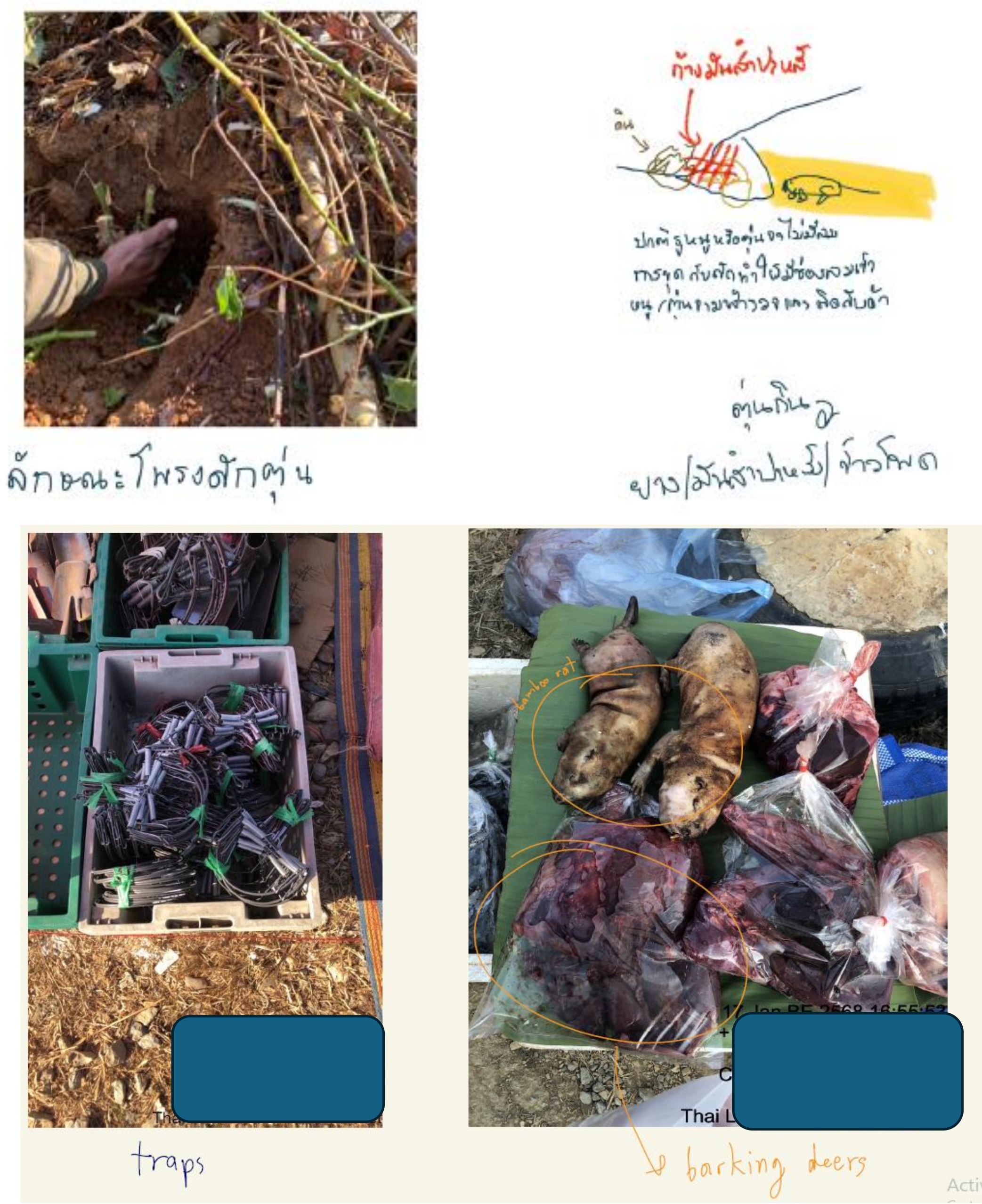	

		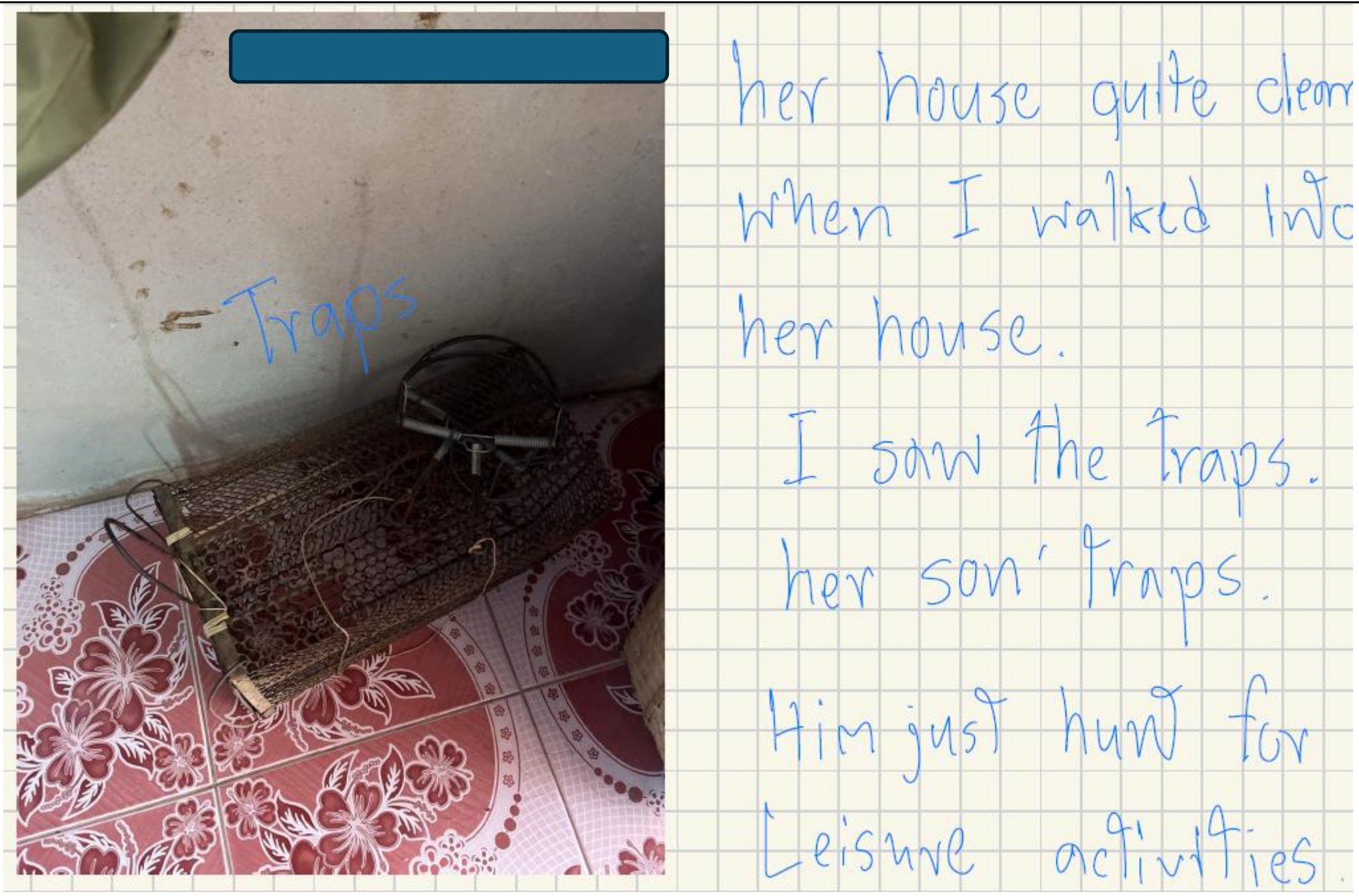	
Hunting with poison	I don't dare eat that kind of meat [from the market] because you never know where it came from. Maybe they used traps or poison in unclean places. There's a small water source near the old school—just a shallow pool where wild animals come to drink or bathe. People sometimes put traps or poison around that area. That kind of thing makes me worried. […] I don't know where they catch it, so I don't trust it enough to eat. […] I've heard of people getting food poisoning—fever and		

	symptoms like that—after eating it. So I avoid it. […] I always assume it might've been poisoned. Without poison, I doubt they could catch that many animals. […] People from outside who bought and ate it got sick. […] Sometimes they poison areas near water sources where animals come to drink. When the animals die there, they just collect the bodies. It's not safe. (Man, 56)<br><br>But in Laos, I wouldn't dare eat the meat. Sometimes they lace the water with chemicals. Once the animals drink it, they just collect the bodies. It happened last year. […] it's Chinese rat poison. Tiny red-topped bottles—10 baht each. They mix it with corn, and just a little is enough to kill an animal. […] just enough water to moisten it, then they add the poison. A bottle about the size of a typical cough syrup bottle. The animal eats it, dies, and then they sell the meat. […] They often use this method for hunting. You have to look at the body. If there's no gunshot wound, it's suspicious. Wild animals usually run away when they see people. If you catch one easily, something's not right. […] If someone wants to fake	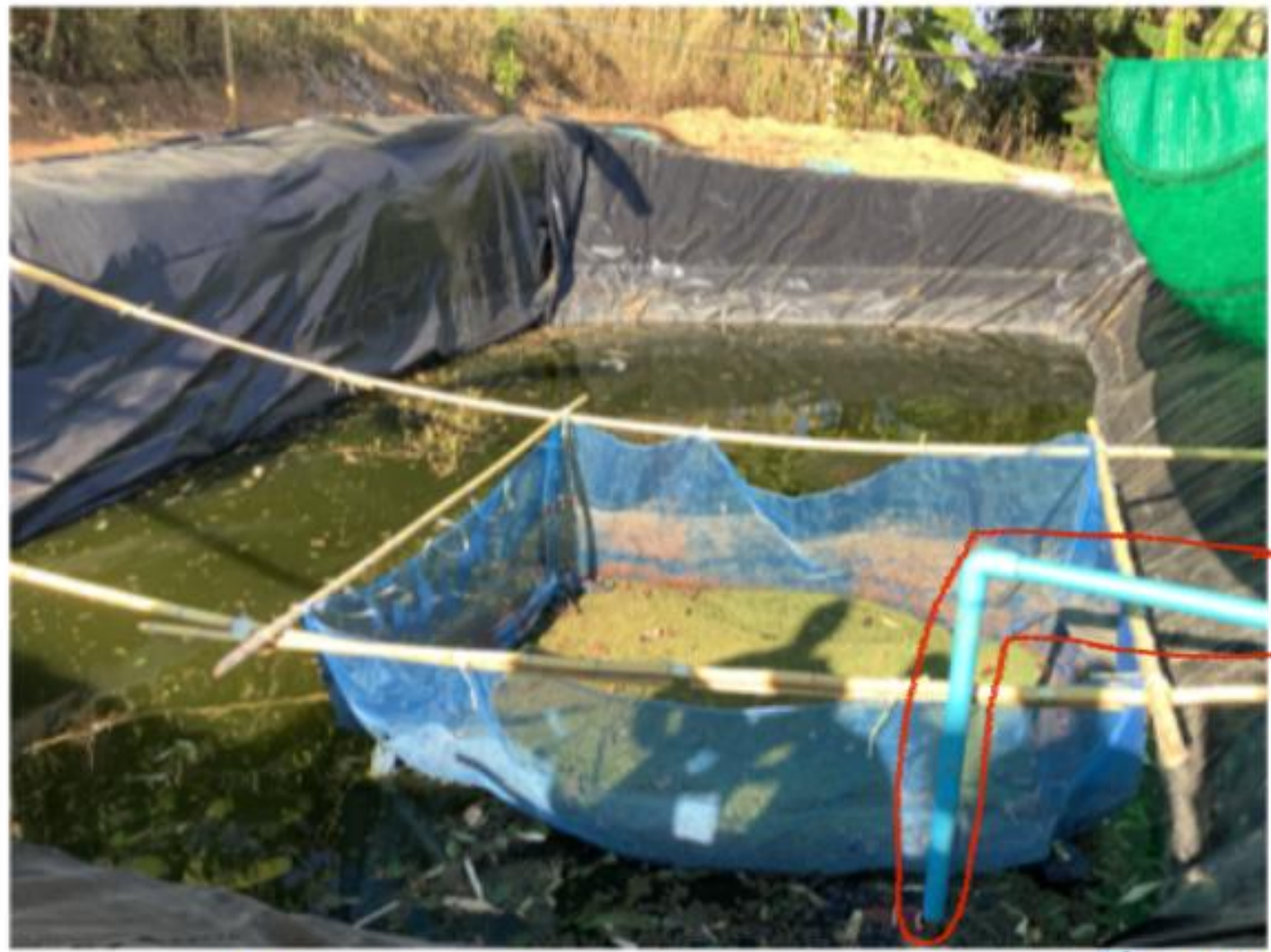 ต่อน้ำลง ไปรดต้นองุ่น<br>บ่อปลาเลี้ยง + แหนแดง (เลี้ยงไว้ให้สัตว์) ปลา หมู<br>นิล	

	a gunshot kill, they might stab it with a sharp metal rod to make it look like a bullet hole. But you have to look at the meat and the body carefully—if there's no bullet inside, you'll know. (Man, 56)		
Slaughtering of farm animals	They usually slaughter animals around festival times—before and after Buddhist Lent and at the end of the year. If they have a forested area nearby, they'll find a water source for cleaning. They keep it simple and do it there, with 4-5 people helping, including the owner of the animal. In our own property, which is located near the forest. Mostly, they slaughter animals near where they raise them because it's convenient. They help carry the animal over, place it down, and pile everything up. Whoever wants a share agrees on the amount beforehand and collects the money before butchering. They call this *pan hoon* (shared investment). For example, this animal costs 6,000 baht. We collect money from everyone in the group 1,000 baht each. Doesn't matter how many people there are in the party but money has to be collected first. […] If we collect 6,000 baht, that means six piles. The	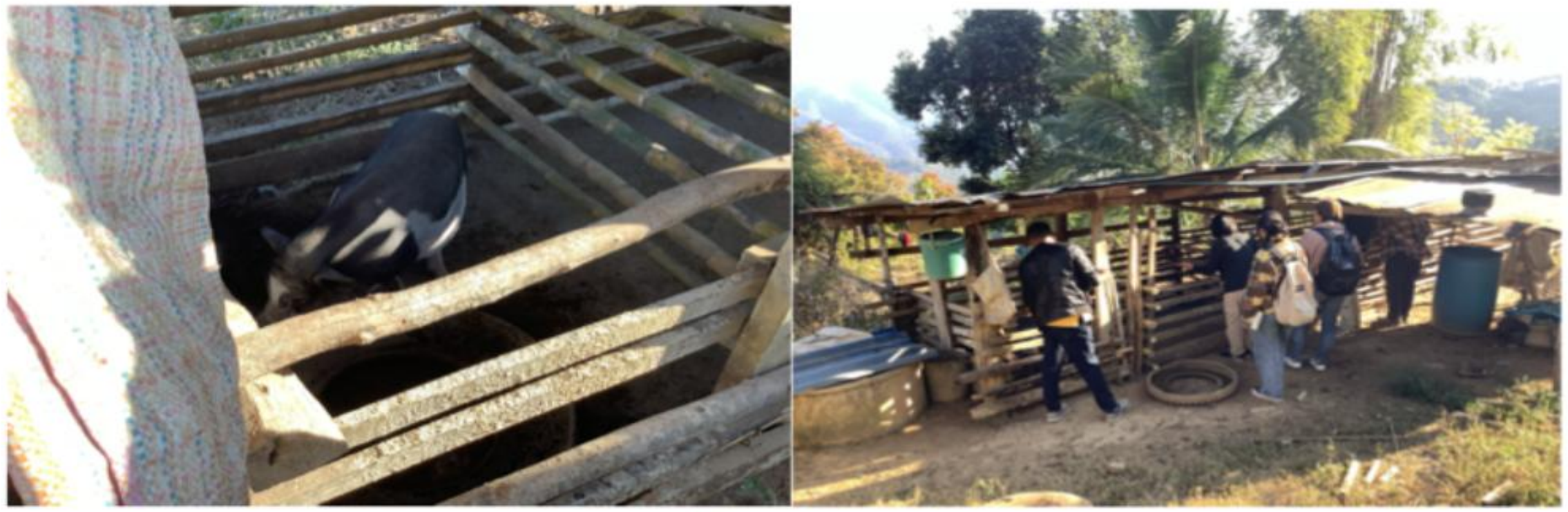 คอกหมู มีไก่อยู่โดยรอบ	

	animal owner also takes part in this. Everyone in the group should join the butchering process to see exactly how much we get, so there won't be any arguments about someone getting more. We should always collect the money before starting the butchering. However, if the portion of the animal we get is a lot, we will share it with the owner. Some parts, like the head, legs, and tail, will be given to the butcher, who doesn't have to be part of the six-person investment.<br>[…] most people keep it for themselves because they know how the cow was raised. Only a few would sell it after the share. Most eat it themselves or share it with others. Since these cows are raised by local people, everyone wants to have the meat. They know no chemicals or substances were used with the cow—it was raised naturally.<br>[…] because it's believed that they have no diseases.<br>[…] people here don't usually buy meat—we raise animals for food. For example, we slaughter a cow for a funeral to help reduce expenses. (Women, 46)		
Meat preparation in forest	He [husband] caught moles. He sets traps with grass. We have moles and rats. The rats		

	like to eat the corn, so we have to set traps. […] I cook. […] He burns off the skin for me.<br>[…] My husband. He does everything properly from the forest. […] Wild chickens. Sometimes they get caught in our traps because they come to eat the corn when it's ripe. […] Sometimes, my husband goes to the creek, and there are stone crabs. He catches those for us to eat. (Women, 43)<br><br>Mostly, it's [butchering] in the farm like this. […] in the areas near the Nan River, if they're butchering animals, they do it right by the river. We also prepare it there [at home]. Usually, when preparing meat, it's done in the farm area. (Man, 55)	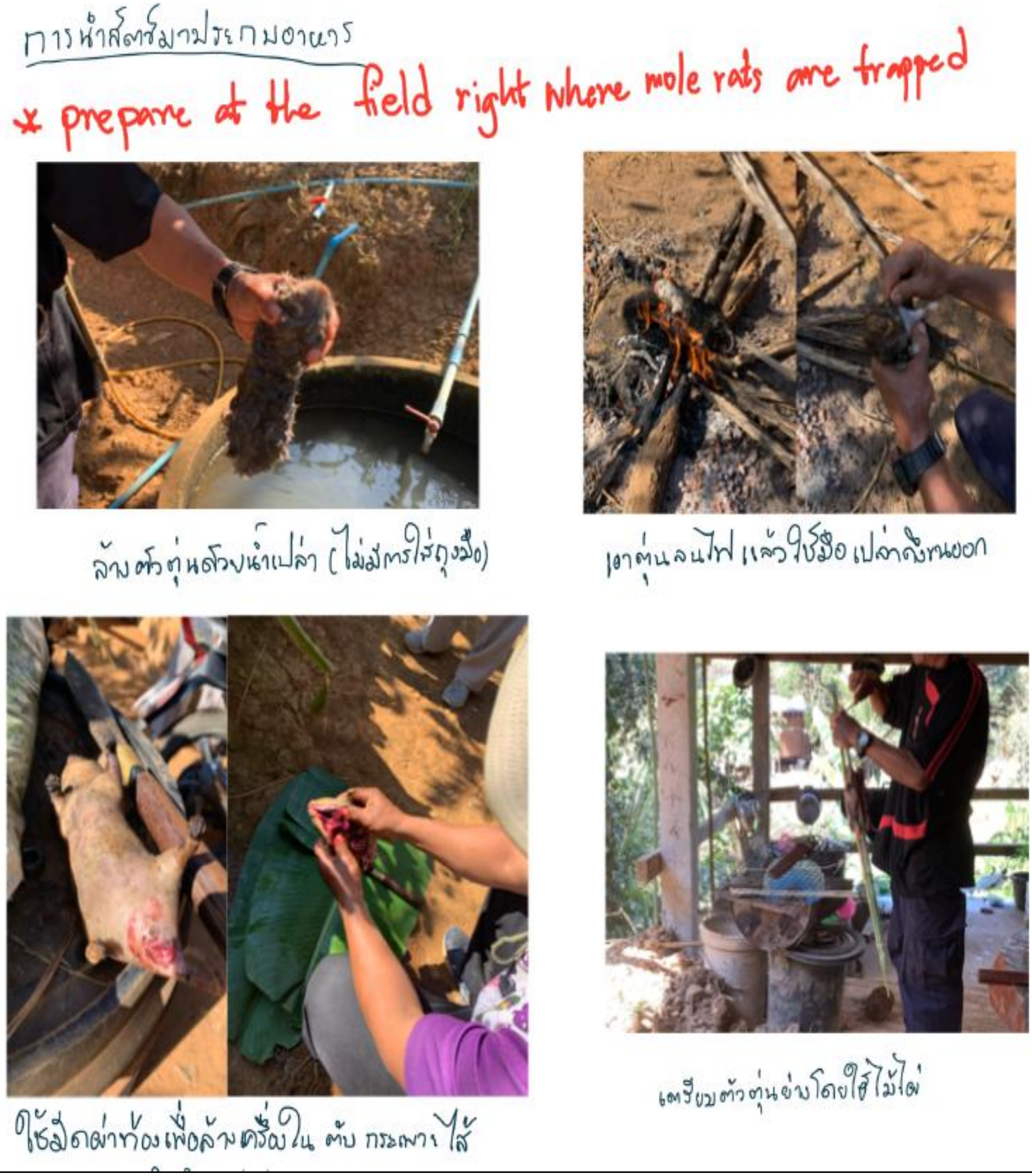	
Meat preparation	I just wash my hands. I burn the fur off, cut it open, and then wash my hands. (Man, 41)		When cooking, she does not wear gloves or other protective

<table>
<tr>
<td></td>
<td>Most of the time, my grandfather is the one who singes the fur, cuts open the belly, takes out the internal organs, and then makes a dry curry or grills it. We use the same one [cutting board for meat and vegetables]. After using it, we wash it. […] mostly he hunts them for us to eat. After we get them, we put them in the freezer. (Women, 35)</td>
<td>
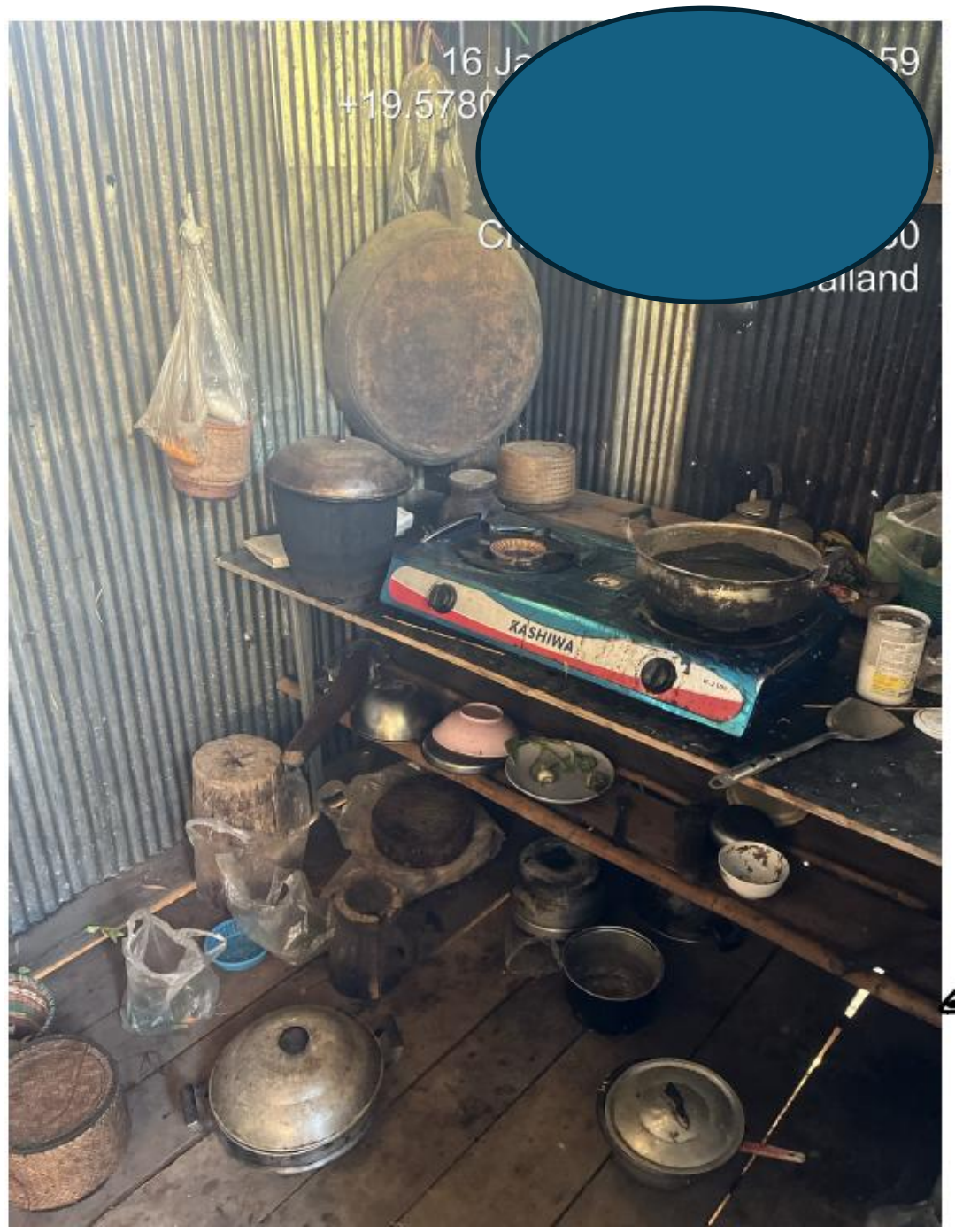
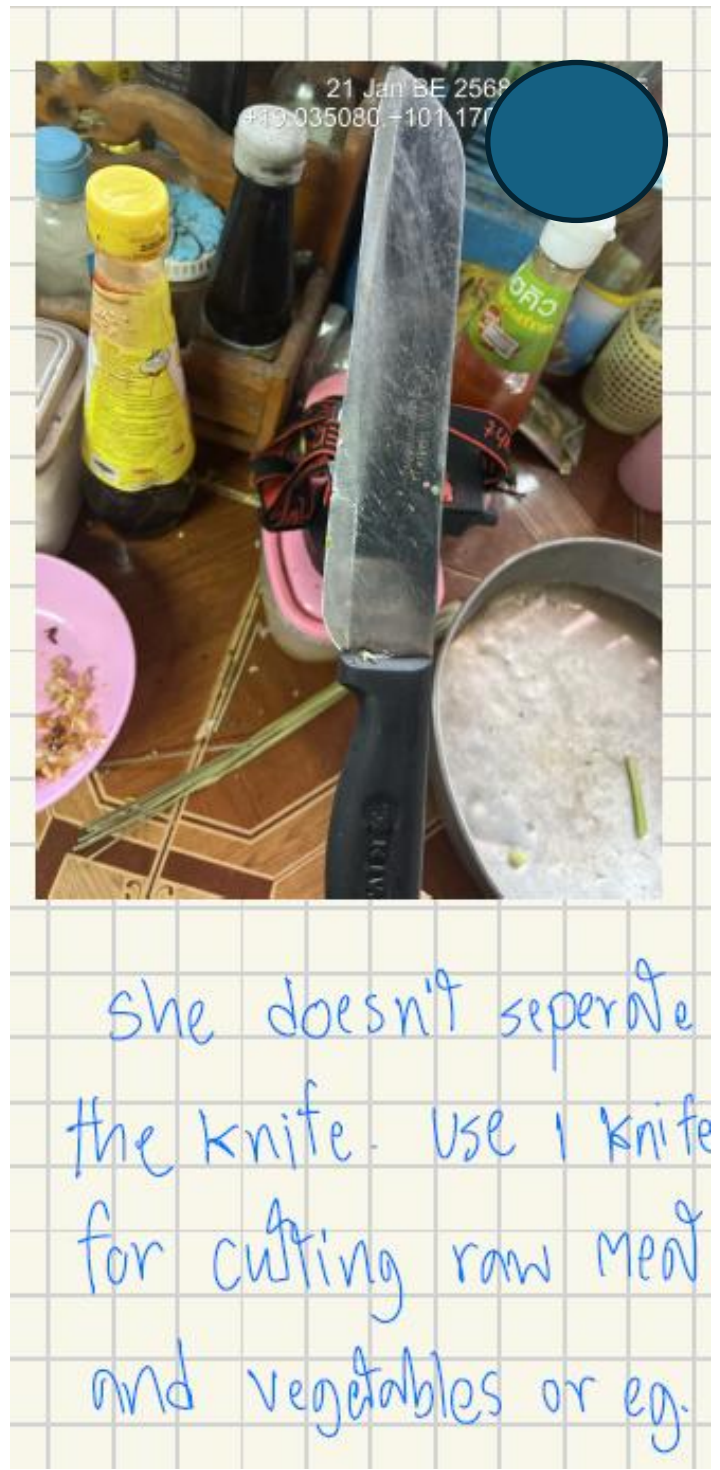

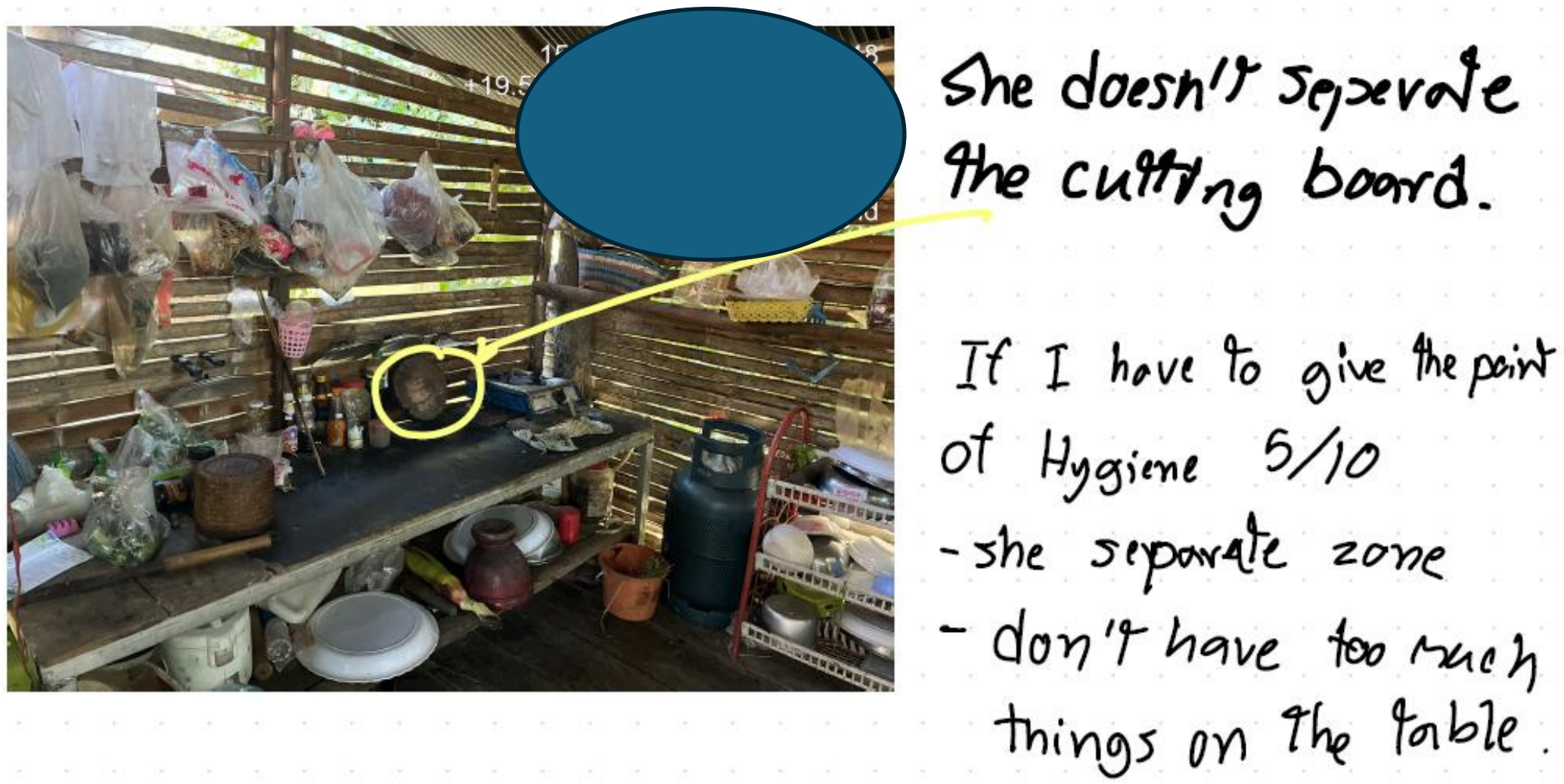

</td>
<td>equipment, but she washes her hands after handling raw meat or finishing the cooking process. (Women, 75)</td>
</tr>
</table>

Judging meat quality	I can tell. The meat will be red, it won't smell bad, and when you touch the belly, it should be firm. If it's not fresh, it will be mushy. If it's not fresh, the eyes will be sunken in. Mostly, I look at the belly. I prefer to buy fish that are still moving. (Women, 35)<br><br>The pork's color won't be bright red; it will look bruised. It will have a smell, especially around the fatty parts. When you touch the fat, it will feel slimy. If the pork is fresh, touching it won't leave anything on your hands. (Women, 46)		
Meat consumption and storage	Field rats, fish, squirrels, birds. Sometimes owls, sometimes partridges, doves. […] We eat both bamboo rats and moles. Moles are mostly used to make a curry with banana flowers and mustard greens. I will make a dry curry and grill the bamboo rats. […] This season is often because the meat is delicious and fatty. Bamboo rats and moles are not eaten often, but I eat field rats almost every day. (Women, 35)<br><br>It depends. Sometimes I go to the forest. Besides, […] There are rats, squirrels, moles, birds, dried fish, and fresh fish. Moreover, in the area we	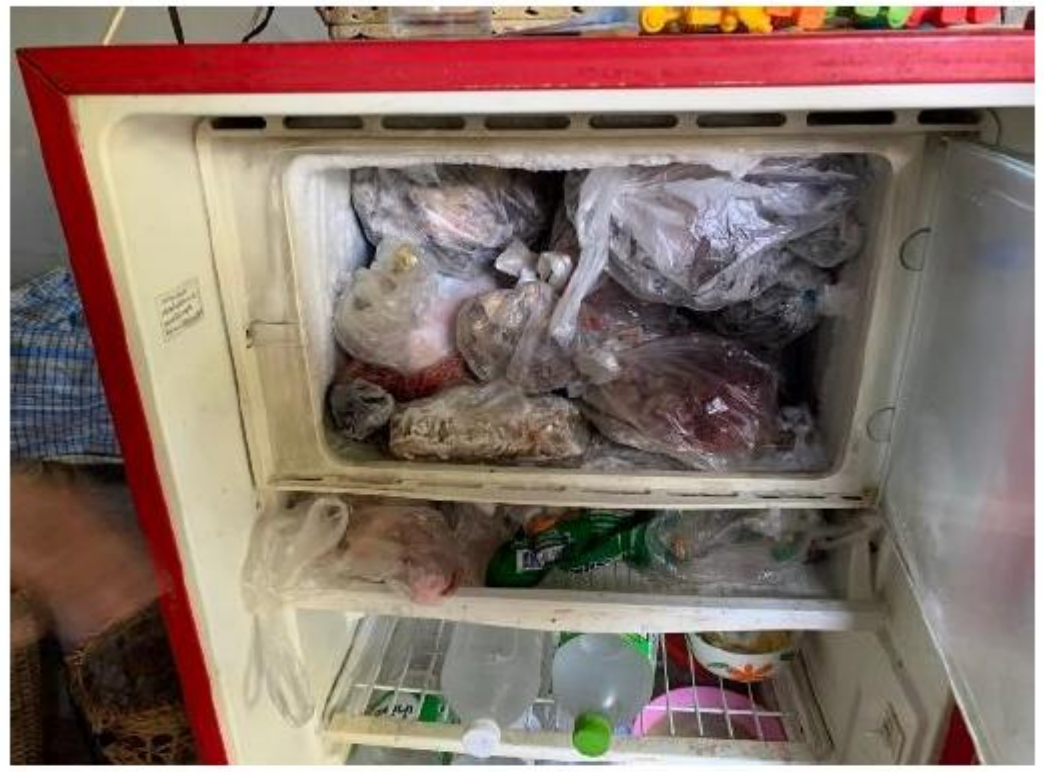 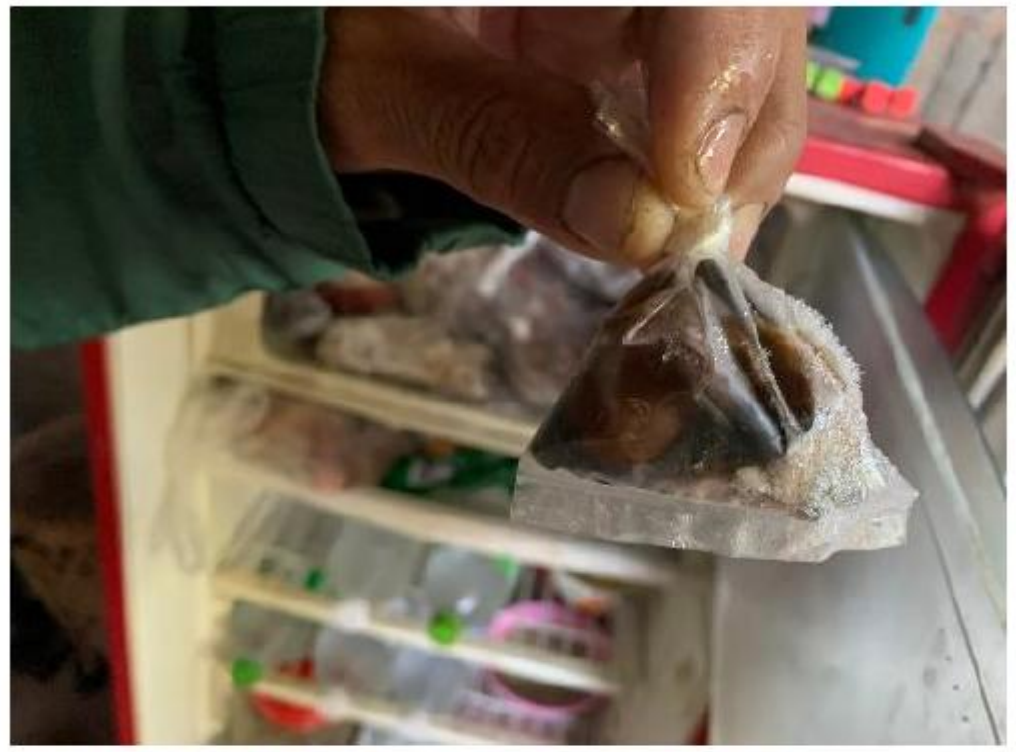<br>All kinds of meats, including fish, pork, and bats, are kept together in the freeze box.	

	hunt, there's Pak Kut (paco fern). It depends. Sometimes, I eat what I buy; other times, I eat what I find. We burn off its hair and clean its organs. Then, we either dry-grill or juicy-grill it. If we juicy-grill, we have to use salt to prevent odors. […] I had that [a bird] yesterday. It's a bird that eats tree seeds, I'm not sure what's it called. [People have eaten wild meat] Since ancient times. People can't go out and buy meat, so they have to eat whatever they can find. (Women, 46)<br><br>We eat wild animals, including birds. Common ones are spotted doves, green pigeons, nok khon—those kinds. There used to be a lot. […] We also eat woodpeckers and nok hua juk (red-whiskered bulbuls). My kids like them too. […] a lot of people enjoy them. The taste is kind of like chicken. Folks who like wild food usually go for them. […] people were too scared [to eat birds in a bird flu outbreak]. It was serious. Around here, birds were dying everywhere. (Man, 56)<br><br>People here eat raw buffalo and raw beef. Most men prefer it raw, especially right after it's slaughtered. For	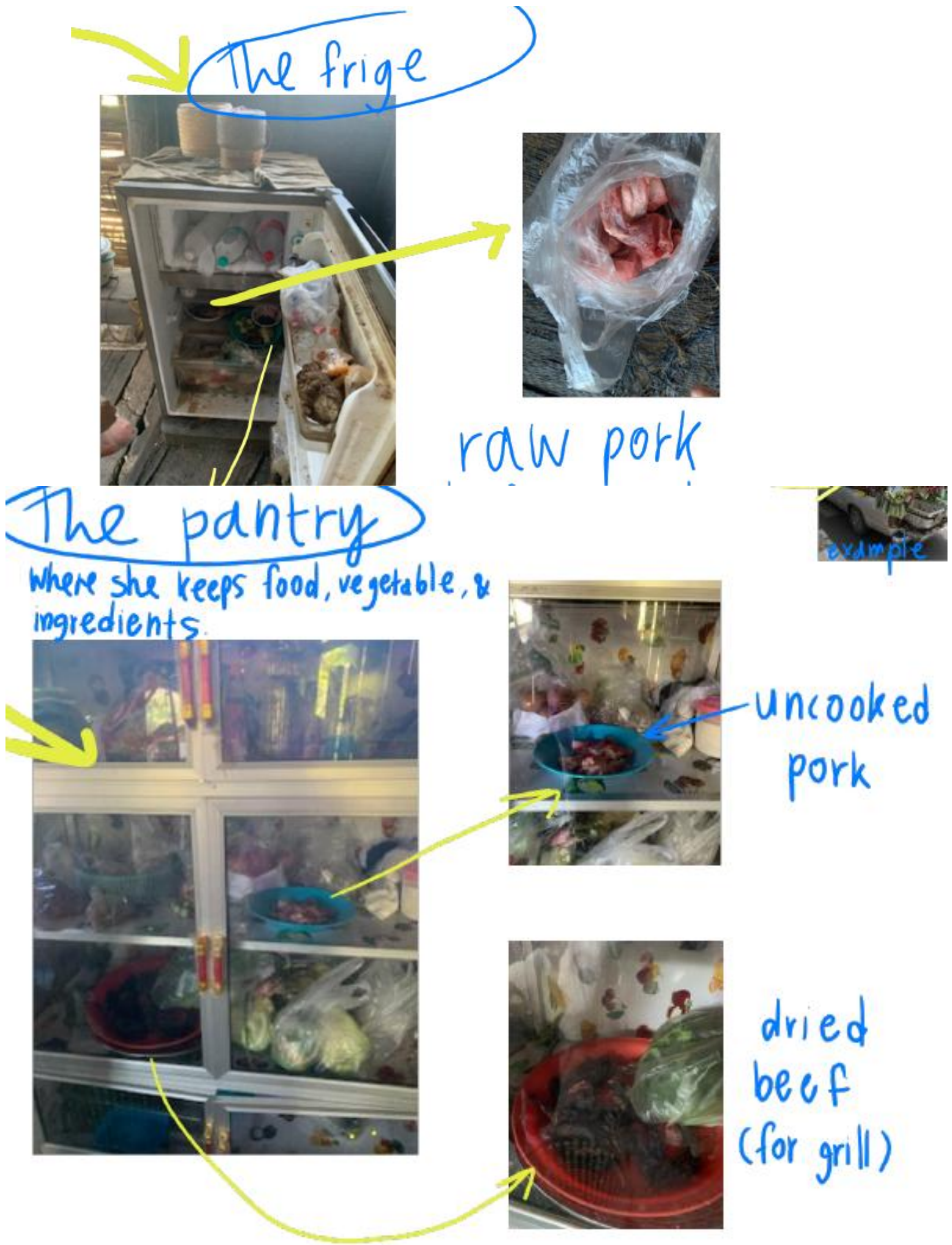	

	example, at a wedding, if there's cattle in the forest, the host assigns 10-20 close people to kill and butcher it. (Man, 46)<br><br>Yeah. People eat both raw and cooked meat. Like "sa," the Isaan dish—that's raw. […] It's delicious—sweet, especially with fish meat. It tastes really good. Same with pork. People used to eat raw pork in larb and no one got sick. […] Nothing much happened. People were fine. But these days, eating raw meat makes a lot of people sick. Nobody dares eat raw pork anymore.<br><br>[…] they're all scared now. People watch videos on TikTok, on TV—they see the warnings and don't want to take the risk. […] These days, if someone gets sick after eating raw pork, people say it's because of a bad smell or some hidden bacteria. (Man, 43)<br><br>People stopped eating wild animals completely [during the Covid-19 pandemic] —it just happened naturally. Everyone was scared. Rumors kept going around, saying the virus came from this or that. (Man, 43)	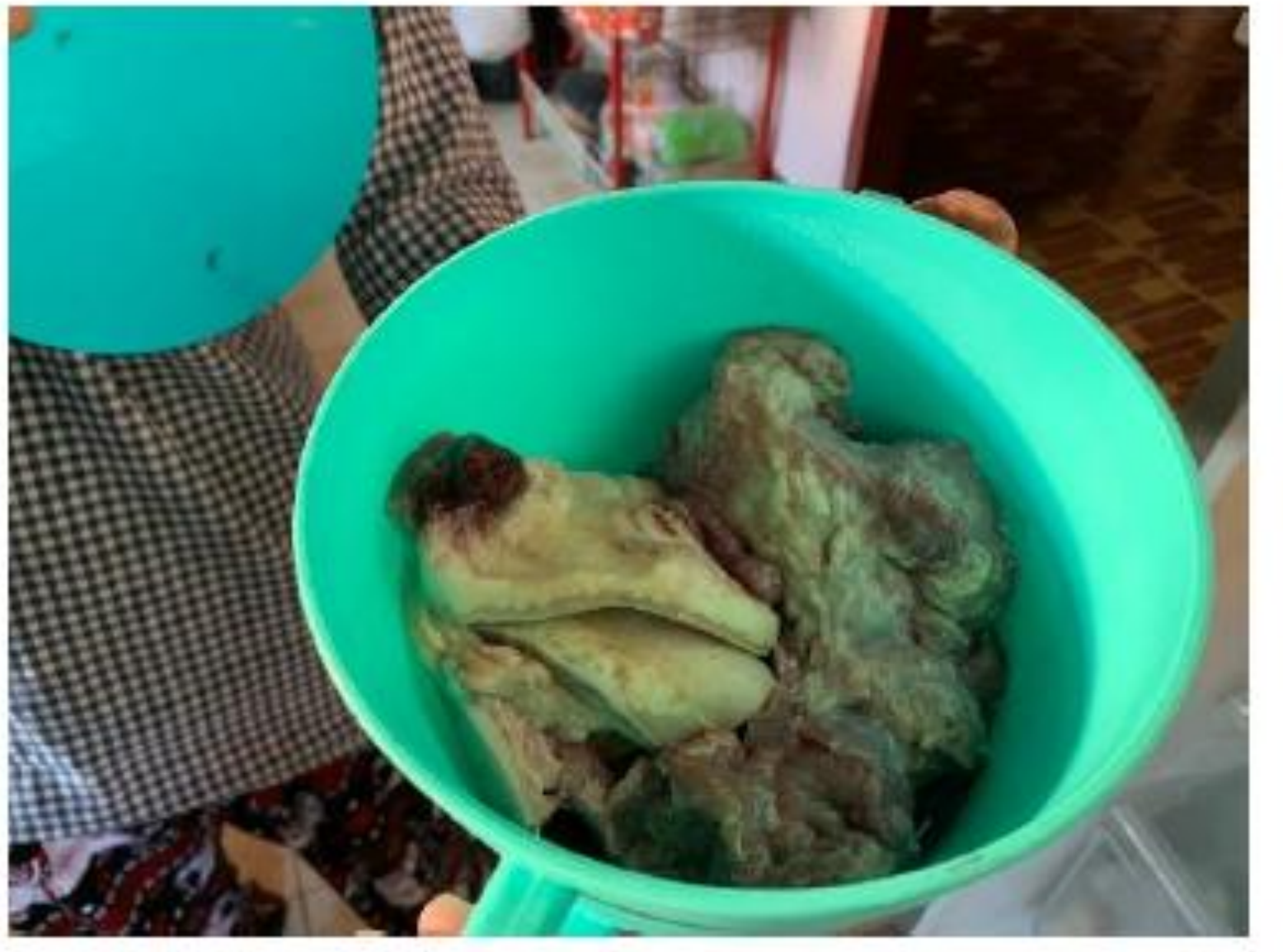	

Meat handling at the market		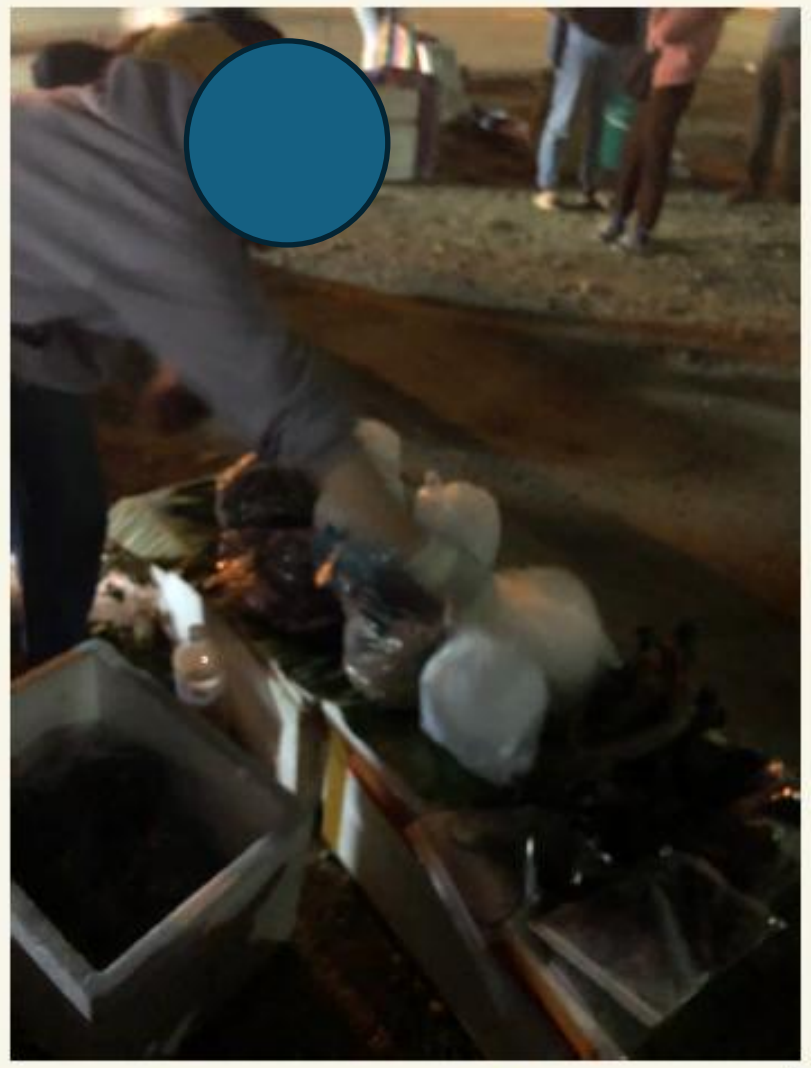 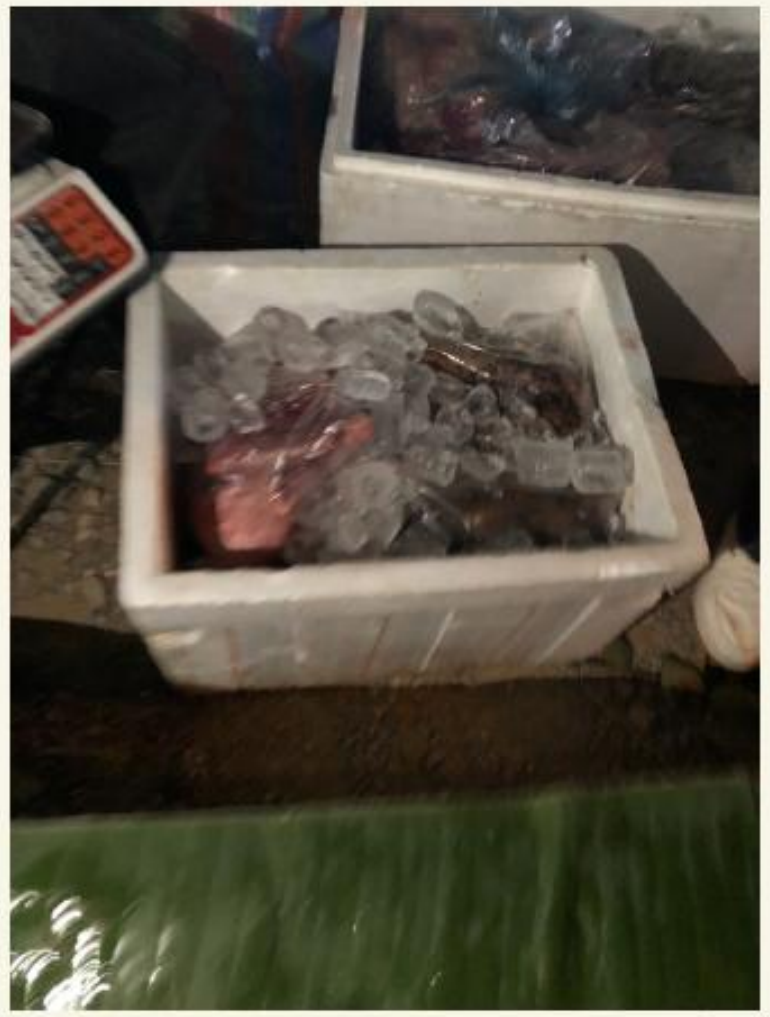 she bought some bags of ice and put it in the boxes to keep the meats fresh and look tasty	

		her warehouse<br>4 she placed all of the stuffs outside because she needed to make room for 7 ppl to sleep together in one place<br>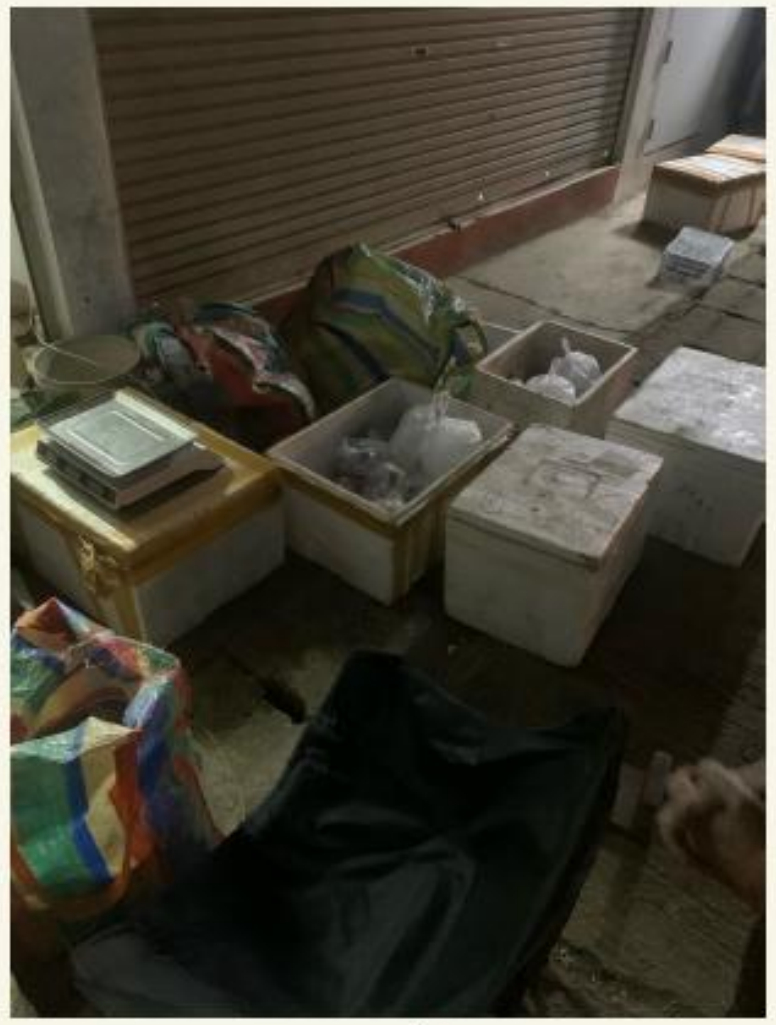 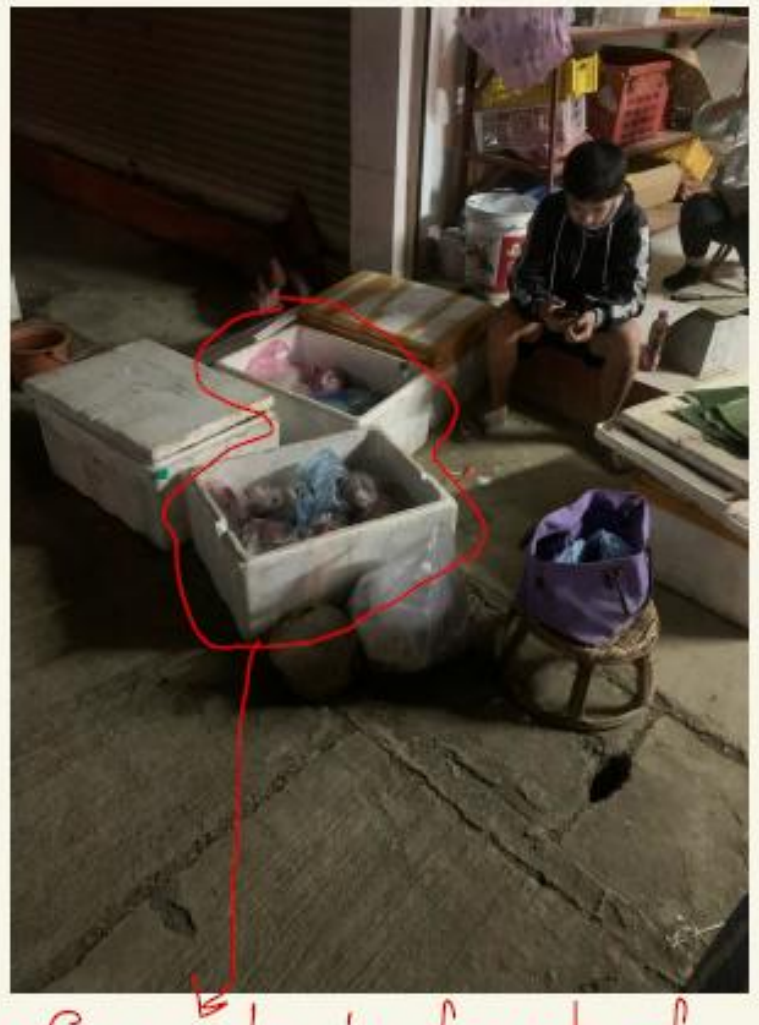<br>They put the boxes full of meats in front of the warehouse		

		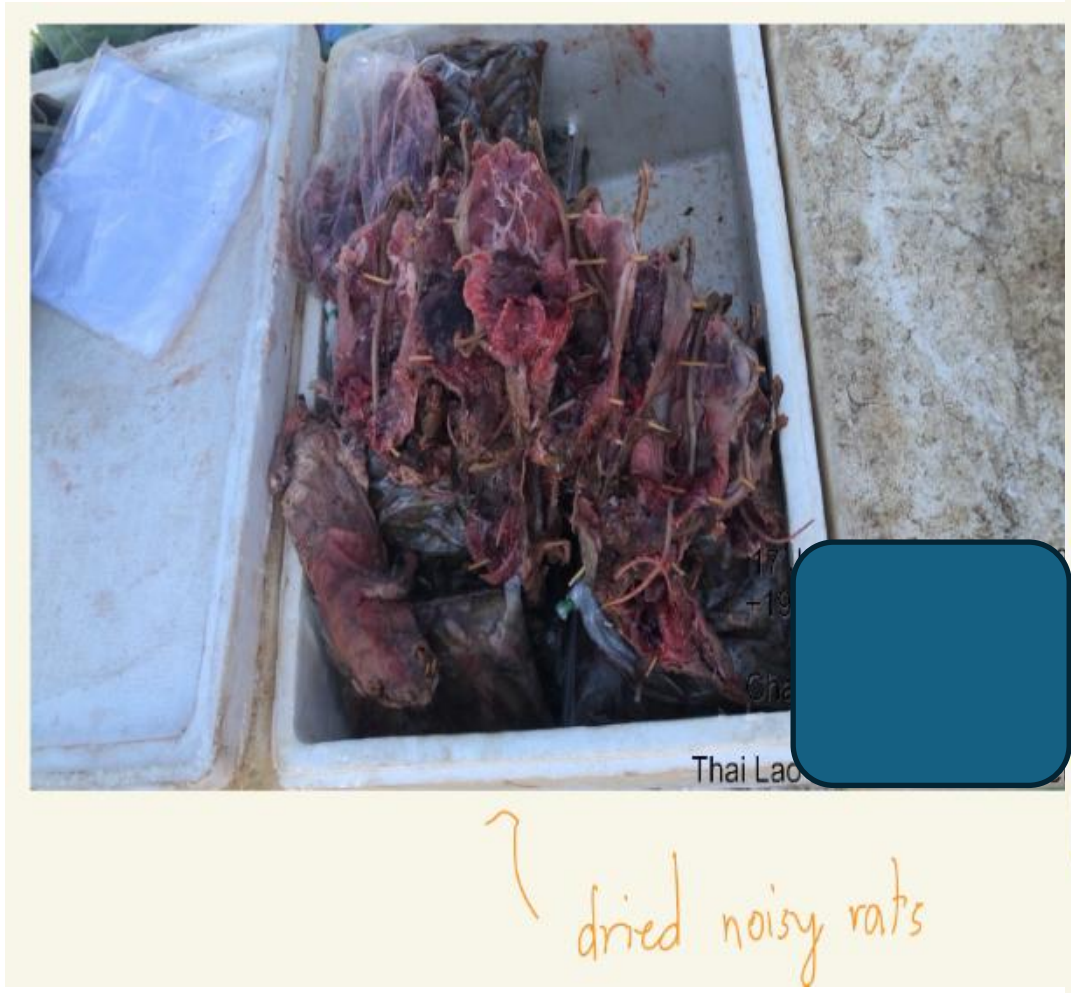 dried noisy rats 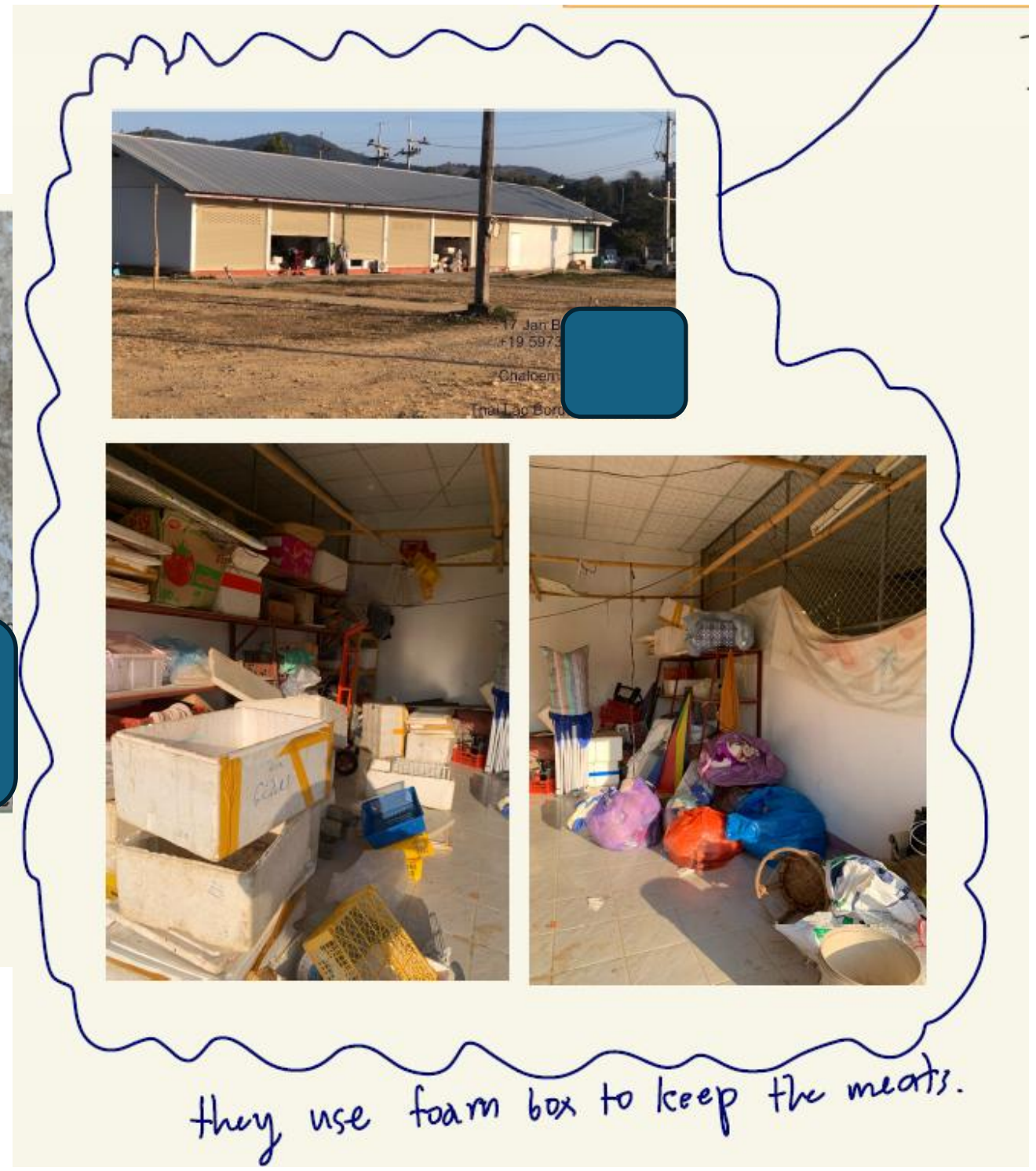 they use foam box to keep the meats.	

<table>
<tr><td></td><td></td><td>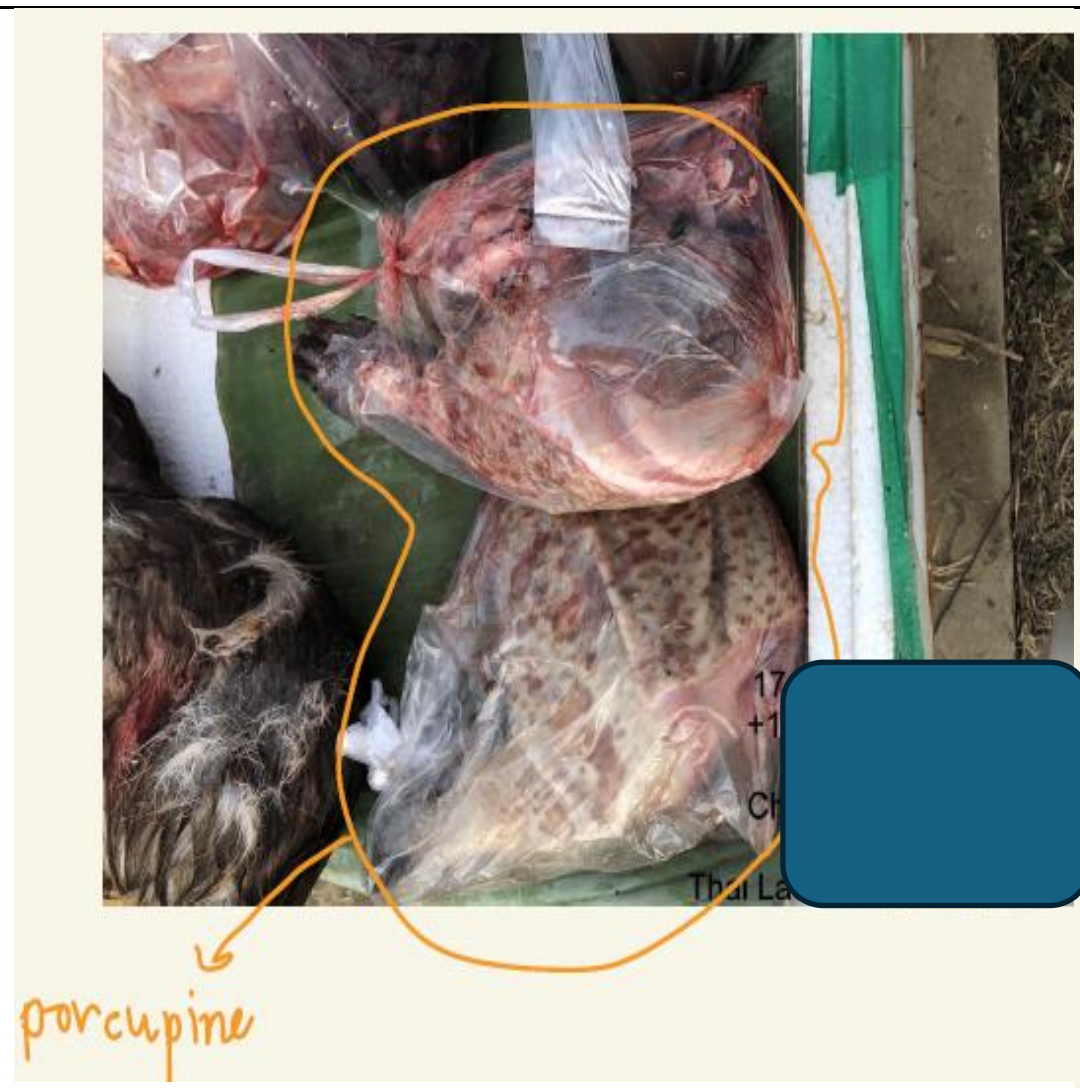

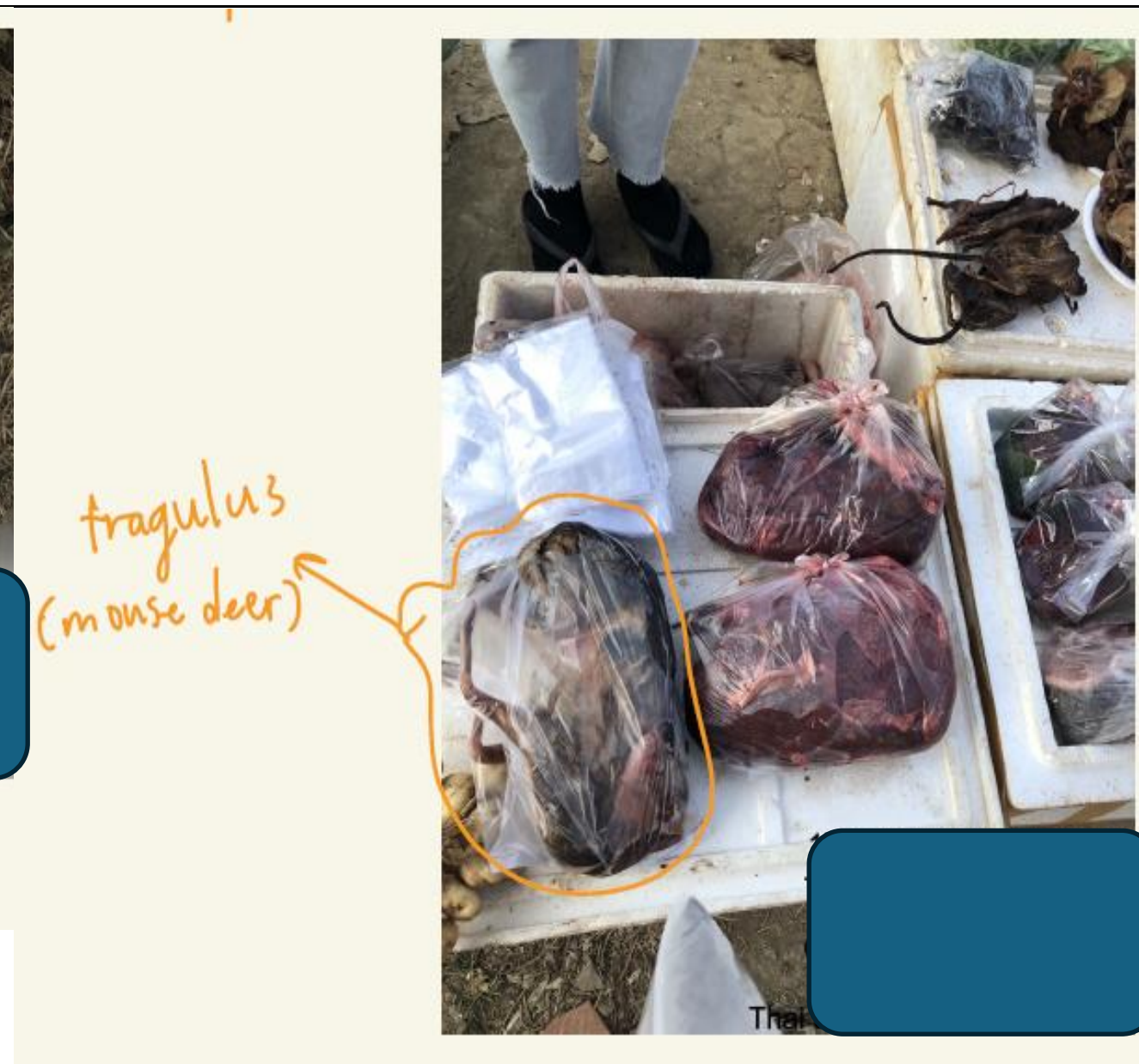

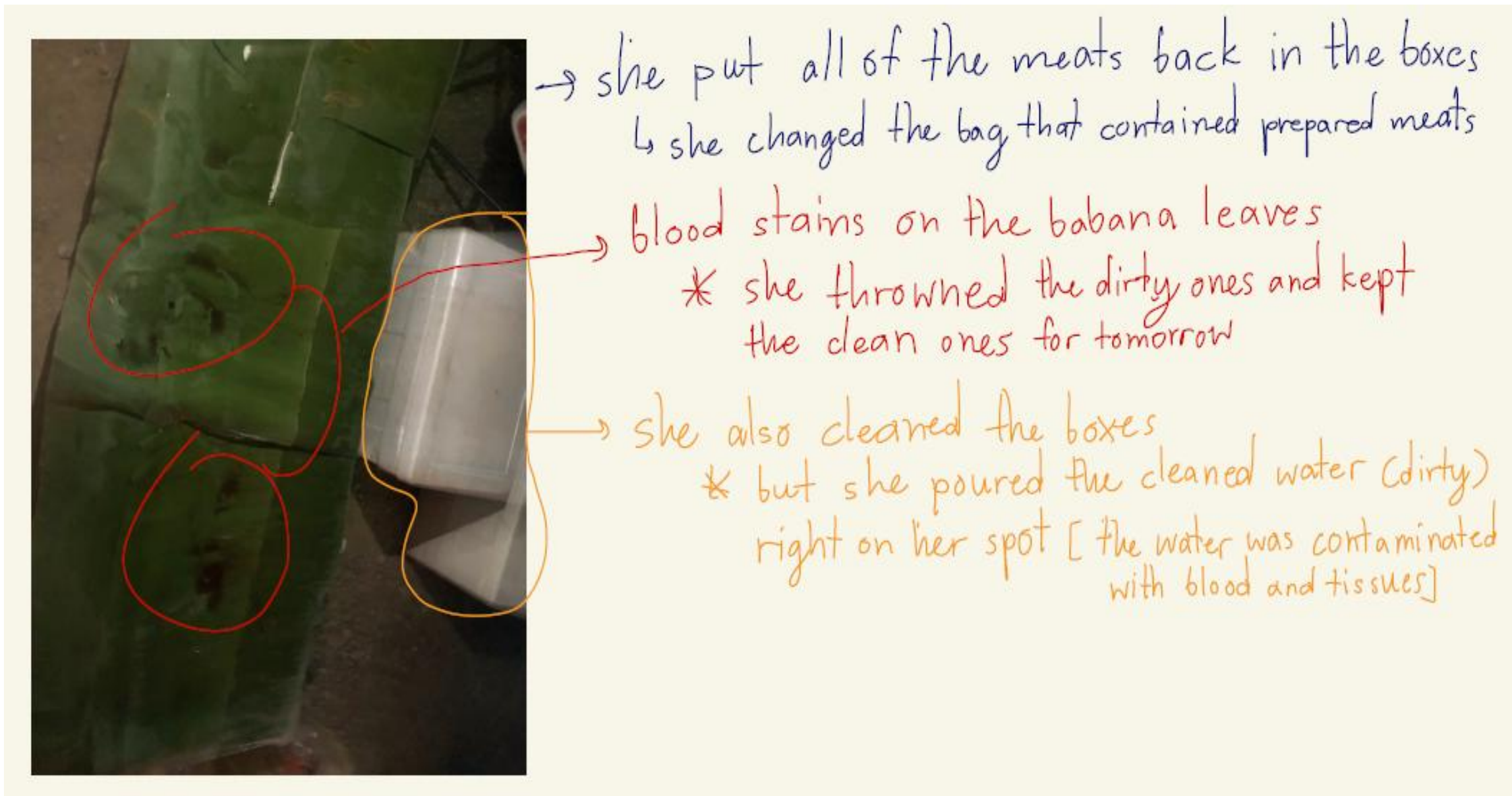
</td><td></td></tr>
<tr><td>Animals in rituals</td><td>In some houses, they really did encounter powerful</td><td></td><td>In the village, there is a</td></tr>
</table>

	spirits. The local spirit demanded raw beef—fresh red meat. I once saw a big ritual in Ban Pon that involved chicken, pork, and beef—all nine kinds of offerings—because they had nine guardian spirits. […] I won't do that. I don't give raw meat or strong-smelling offerings to those spirits. Because spirits that consume raw food aren't the kind that can live peacefully among people. They don't belong in villages. They're nomadic and aggressive. If we feed them once, they'll want more. And they'll keep coming back. […] Then they start possessing people—causing illness, stiff eyes, foaming at the mouth, alternating fevers and chills. It's like something strange has taken hold, and it's hard to remove. […] in our belief, they're malevolent spirits—ones that crave raw, bloody offerings. We believe that if we give in to them, they'll take over the whole village. I use other methods—cooked rice, young green rice, sacred water from som poi, sacred leaves. Then I call on the guardian spirits or benevolent ones to drive them out. I absolutely will not appease evil spirits. They're not good spirits. They show up uninvited, always wanting	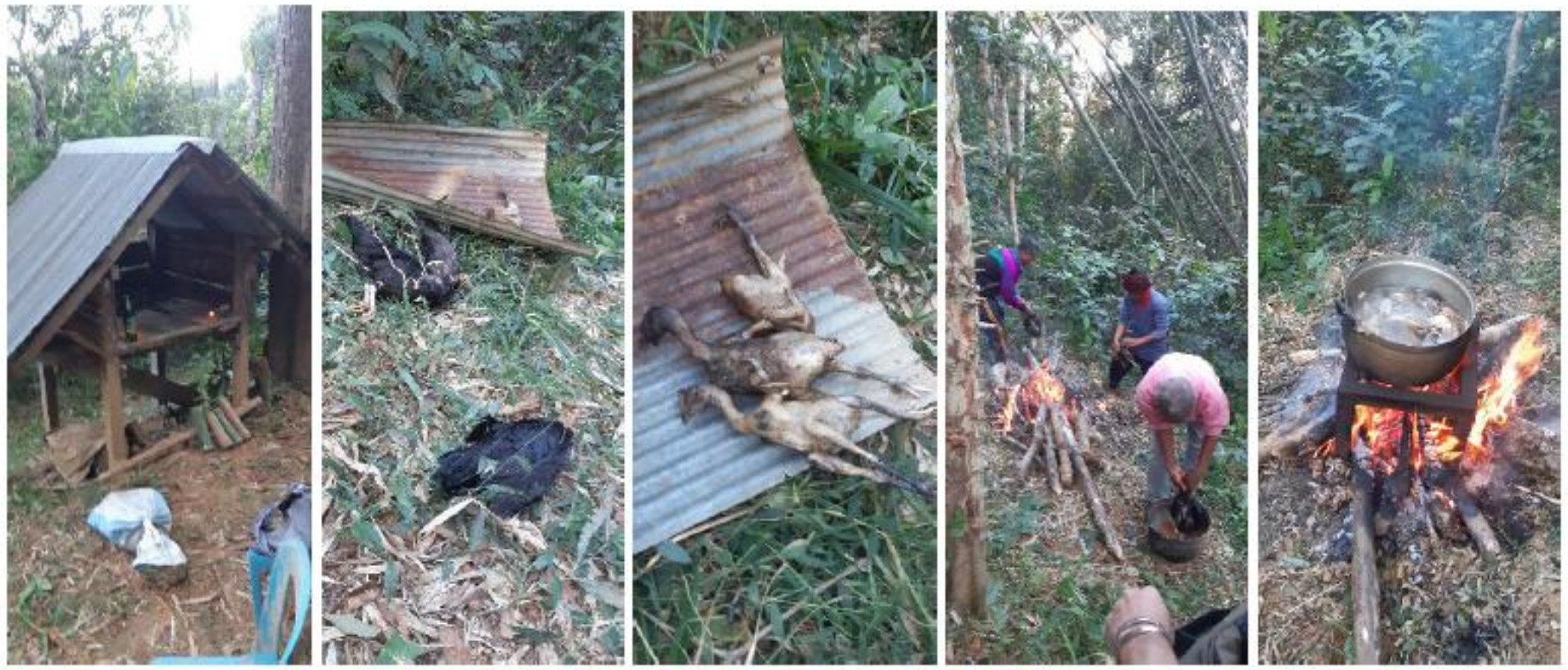 **3) The Sacrifice** Seven young female chickens/hens were brought to the ceremony as oblation for the sacrifice. As belief, chicken is the symbol of purity and linkage to the hereafter world because it has wings and legs to carry prayer to the gods by both frying and running. The numbers of oblation depend on the successful prayer which villagers or whoever received. This time there were 7 people get the success blessing and someone in this group were high status government officers from Thai and Laos. By the way, all process of chicken preparation were done by bare hands. They hit the chicken heads with wooden stick by disciples of sorcerer head, but they incompletely died, some of them still moved on the ground. And then those dead chickens were drenched in water before throwing into the bonfire in order to remove feathers and finally cut them into pieces. However, intestines were thrown away into the bon because the forest gods and wisps don't like stinky disgusting organs. The chickens were cooked without any season at the medium rare. By the explanation of participants, they said the gods and wisps like eating natural taste.	tradition called "Kwan Khao," which is a ritual to honor the spirits before starting the new planting season. The family members must work together to prepare the offerings, which involves slaughtering a chicken and then boiling or steaming it before offering it to the spirits. After the ritual is completed, the chicken meat becomes food for the family members. This tradition is carried out both at home and in the agricultural fields, where the chicken is

	something. Always hungry. No matter how much they get, it's never enough. Some like raw beef, others like raw eggs or fresh blood. Just one look and you know they're not the local protectors. […] Once they've had it, they keep coming back—every year, every season. Sometimes they even claim they're here to help or protect. But really, they just come to take. They have no sense of how people here live. They've never even smelled the earth in this place. I don't feed them. No appeasement. No raw food. No blood. I don't try to satisfy them. Spirits like that can't stay long if we don't feed them. Eventually, they leave. What I do is call on the good spirits—the ones who truly guard this place—to help drive them out. This village is not a place for wandering spirits. Those ones only want to take. […] If it's that kind of spirit, I don't welcome it at all. That's not a household guardian spirit. Spirits that eat raw things are too powerful—they can't live with people. Once you feed them, they latch on and keep haunting people in the village. […] Once they've tasted it, they won't leave. They'll crave more. And if they don't get it, they become stubborn. In		slaughtered at the specific location. (Women, 75)

	some households, kids fall off their bikes, people fall ill—all because they gave offerings to that kind of spirit. These aren't spirits that can live peacefully with us. […] . Good spirits don't take things that harm people. Offerings to good spirits must be clean and cooked. If it's a spirit that eats raw or strange things, we drive them out—tell them to go feed off some other land, not here. (Man,63)<br><br>If it's not something directly used in the ritual—like feathers or chicken intestines—we just bury or burn them. There's no need for any extra chanting, but we still clean it up properly. Since those parts were involved in the ritual, we don't let them sit around messy. […] It depends on the timing. If the ritual is happening and the auspicious time arrives, we offer it as is. We can finish cooking it afterward. Good spirits don't care whether the meat is fully cooked or not—they care more about our sincerity. (Man,63)<br><br>They have to wash—definitely. Their hands must be clean. Because it's a ritual, both body and mind have to be pure. Anything stained		

	with blood has to be washed before touching food.[…] for washing hands, dishes, pots—everything must be clean before preparing food. Otherwise, the good spirits won't accept the offerings. And when we bring items up to the mountain, we carry water and utensils too. No one reuses things from home, because anything used in the ritual is considered to belong to the spirits. […]. Those items are never brought back home. If they break or wear out, we replace them with new ones. The old ones are buried or left at the ritual site—as a way of returning them to their spiritual owner. […]. They belong to the spirits. If you bring them back home, something bad might follow. [hahaha] The elders take this very seriously. […] banana leaves, tamarind leaves, guava leaves—they all work. They're like natural sponges. Since we don't carry too much up the mountain, we use what's available in the forest. But even then, we have to ask for permission before picking leaves—otherwise the spirits might be displeased. Cleanliness is very important. All food has to be thoroughly cooked—raw food is strictly forbidden. Good spirits don't consume		

	anything raw because it's considered impure. (Man, 63)<br><br>No, if it's sick, we don't use it. […] we have to find another one. […] one that is healthy and that we can eat after the ritual. (Man, 72)<br><br>Well, we prepare two chickens as offerings in the field, and two more chickens at the edge of the forest to appease the spirits.<br>[…] After the ritual, we eat the chickens. We don't just let them go.<br>[…] Usually, we hold the chicken by its legs and hit it with a stick.<br>[…] we sprinkle the blood where we do ritual, especially on the bamboo structure. […] We leave them for ten minutes. We offer them to the spirits first, but since we can't see them, we just follow the tradition. After about ten minutes, we tell the spirits and get the chicken back. […] we've never seen any evidence that the spirits have eaten the offerings. (Man, 41)<br><br>Firstly, we need chicken, liquor, and other items as a tribute to the spirits. We perform the *Slod* ritual, which involves a ceremonial bracelet or sacred objects		

	placed in the fields. We sacrifice a chicken and call upon the field spirits, pray for prosperity of farming and take a vow to give them compensation. This is the way we seek blessings for rice. This is part of the Kwan Khao (Rice Spirit) ceremony, where we symbolically bring the spirit of the rice from the field into our homes. […] Yes, we do [slaughter the chickens ourselves]. […] it must be cooked. First, we place the blood on the rice plants. Then, we cook the chicken and offer it to the spirits again. Then comes the Songkran festival, where elders are honored through water-pouring ceremonies. (Man, 52)<br><br>They still eat the meat. I've heard stories like that. They cook the meat before eating it. We always advise them not to eat raw meat from a cow that died without knowing the cause. They have to cook it thoroughly before eating it. […] If it's Laab from the market or meat from a live cow that they just slaughtered, then yes, they eat it raw. They eat raw Laab and soy Ju. [But] not very often. Only during festivals when they slaughter cows or pigs.		

	Like after Buddhist Lent, New Year's, Songkran, New Year's Eve, they'll slaughter pigs and then invite everyone to come and eat together. Like, "We're going to have a countdown here and slaughter a pig," things like that. They only slaughter cows and pigs during festivals. (Women, 50)<br><br>There is a cockfighting tradition. My husband raises chickens for that purpose. He has one rooster for fighting. (Women, 49)		
Raising farm animals	For cattle, we don't allow them in the household area. […] we separate the zones for cattle. We've always done it this way. (Man, 55)<br><br>M: When we walk around the village and see pigsties, are they usually located as far from the houses as yours, or are they closer?<br>KI: There are some [pigsties] that are closer. […] Mine are on the other side. I have to use EM (Effective Microorganisms).<br>To reduce the smell. Pig manure can be fertilizer for our vegetable garden. […] We use disinfectants. [But] only if there's a disease outbreak or something like that. (Women, 50)	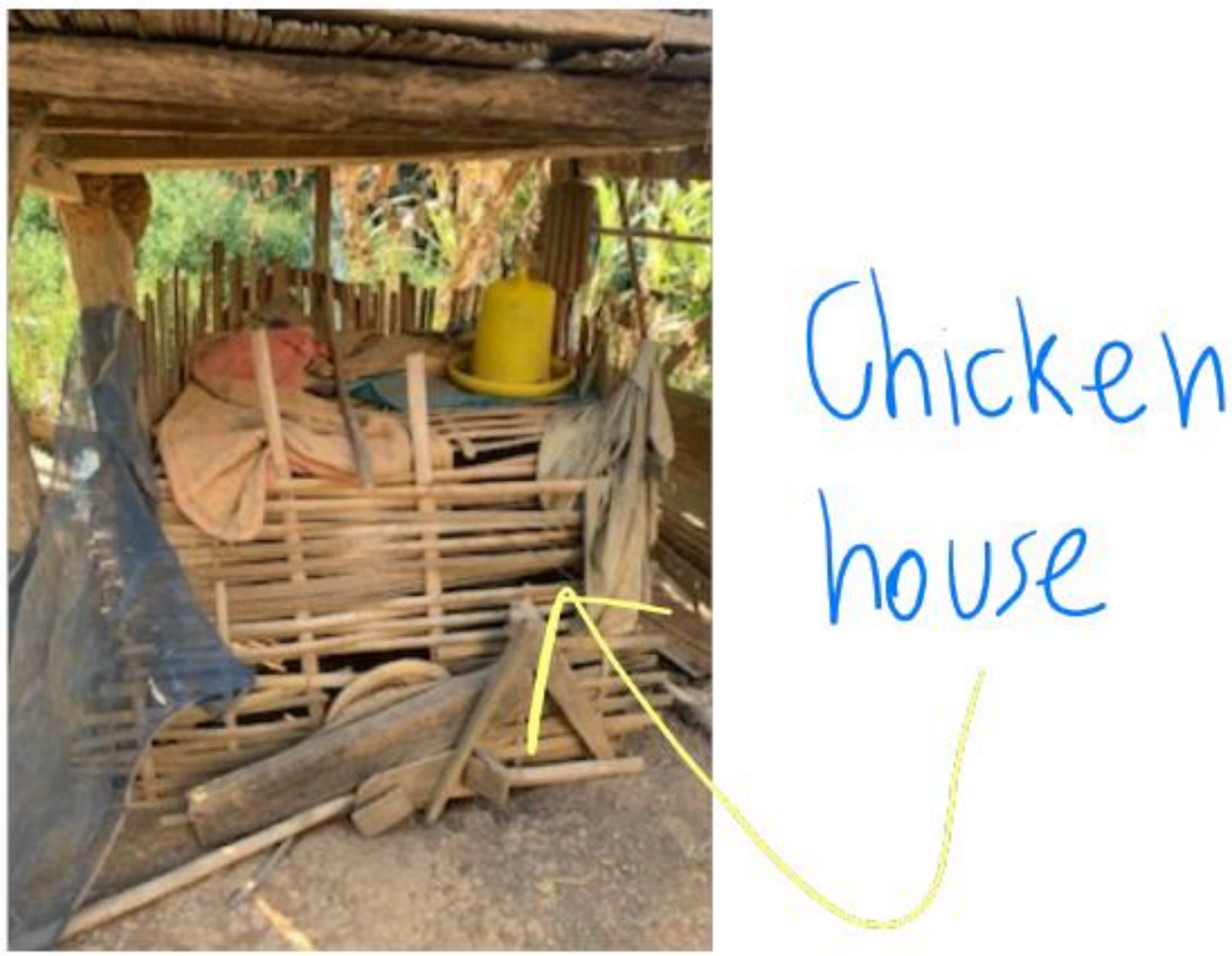	**Most of villagers usually farm these local pigs in the re-used furniture parts built as simple pig pen. The pig is used for domestic consumption and for ceremony. The baby pig is popular for using in the wedding of Lua ethnic.**

Nowadays, there's no more raising chickens under the house. Every house has separate coops. Most houses here, the uKMer floor not very high from the ground, even if they could raise chickens under the house, they don't. They raise them in separate coops or in their gardens. At home, they can take care of them more closely. But in the gardens, they only check on them in the evenings and mornings. I think most people raise them at home. But if they're in the gardens, they're more likely to be attacked by dogs. The reason people prefer raising them in the gardens is because their houses are close together, and if someone's chickens eat another person's crops, they might argue and fall out with each other. […] mine is about 10 meters away. I used to raise them in the orchard, but dogs killed them all. So I gave up on raising them there. (Women, 50)

If the pigs are raised near the house, it does affect health because of the smell. They shouldn't be raised near the house; it's better to keep them in the farm or in an outdoor pen. […] chickens can also be a problem. If they get sick, they can spread diseases to

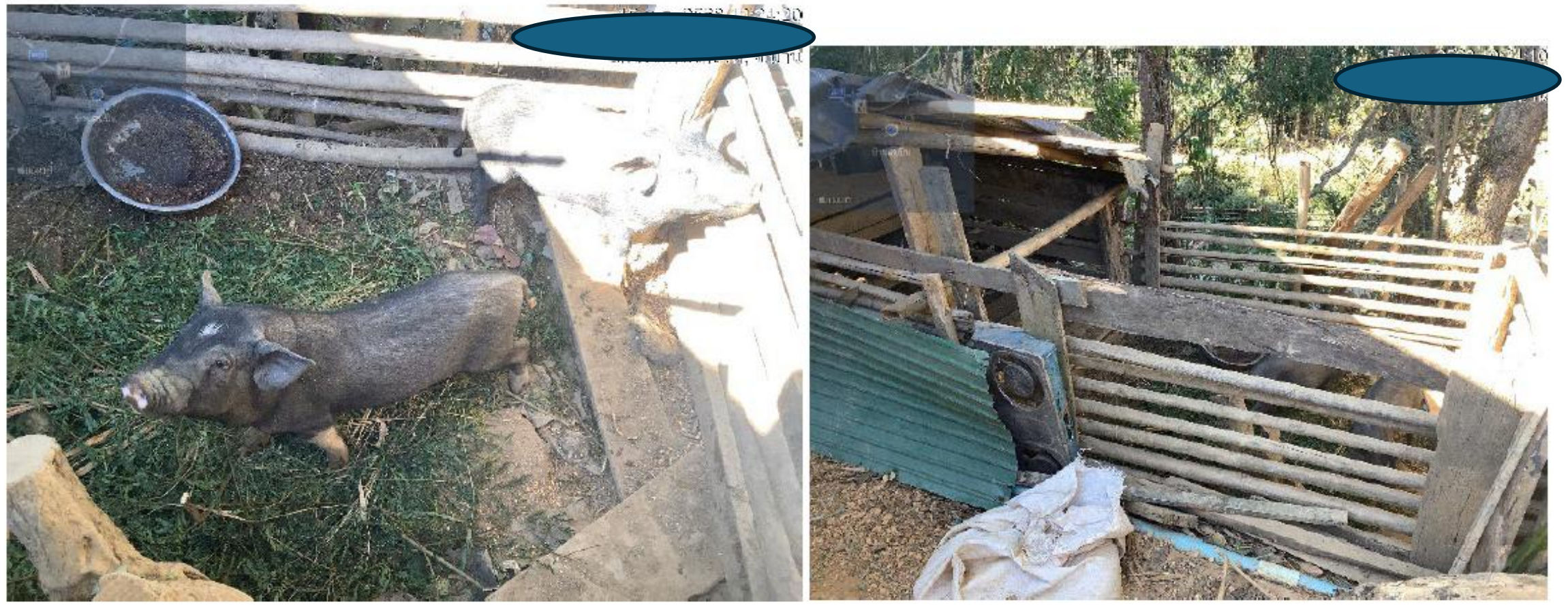

5) Local Pig and Pig Pen

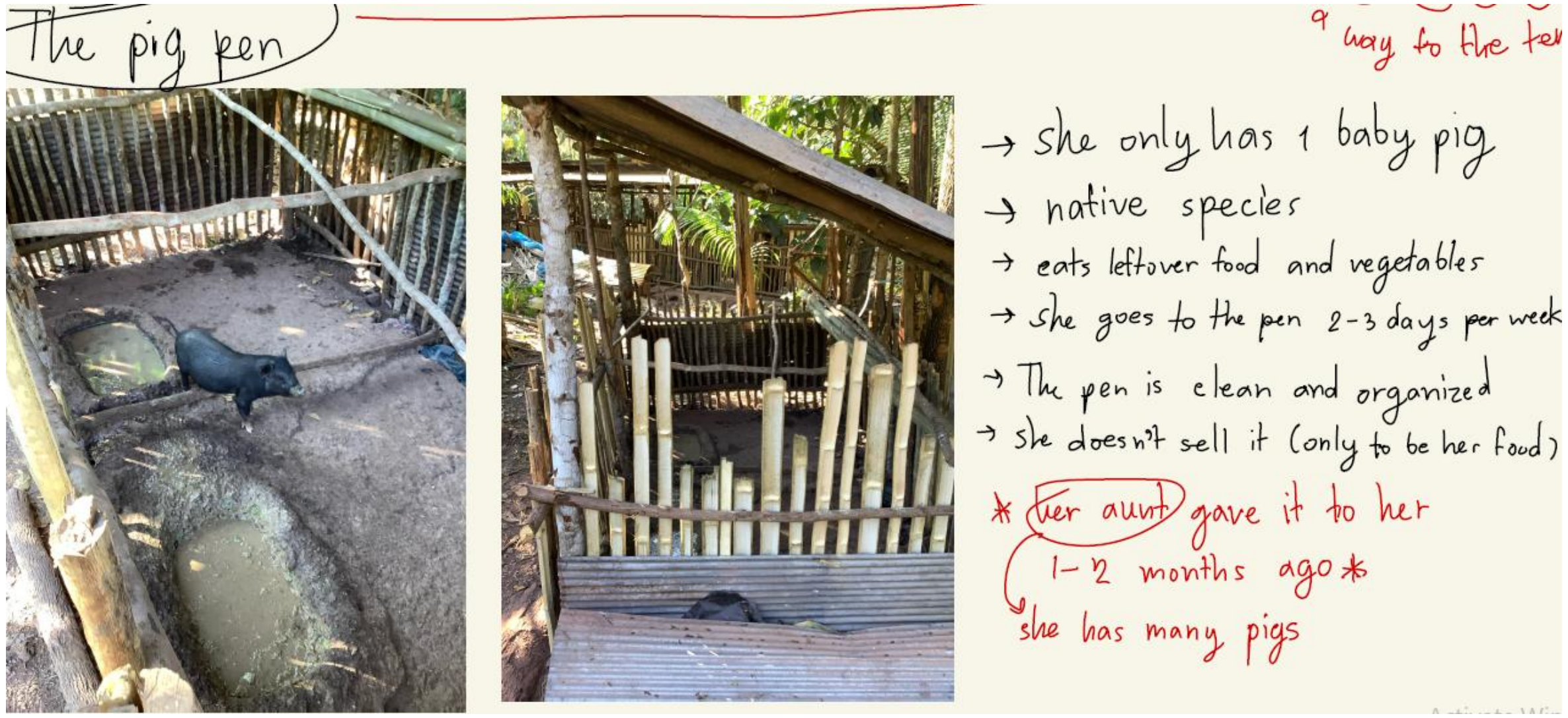

<table>
<tr>
<td></td>
<td>humans, which can be dangerous for children. Cats are also a concern because of the fur, which can cause allergies, especially in kids. In my village, people don’t raise cattle near their homes. They usually keep them in the field. If we talk about the potential health impacts, it’s the same animals that shouldn’t be raised too close to the house. It’s better to have them in a farm or on a large piece of land. (Women, 49)

No, there are no enclosures for dogs or cats. Most people have pig pens beside their homes, about 3-4 pens. Dogs and cats are left to roam naturally. Chickens roam freely, but sometimes there are small enclosures, though the chickens don’t stay in them. They tend to sleep in trees, and sometimes they sleep in front of houses, leaving droppings that create unpleasant odors. It’s not ideal to raise them like that, but we can’t tell them because they have been doing it for years. To improve health, it would be better to raise them at a farm so that disease outbreaks don’t spread in the village. (Women, 49)</td>
<td>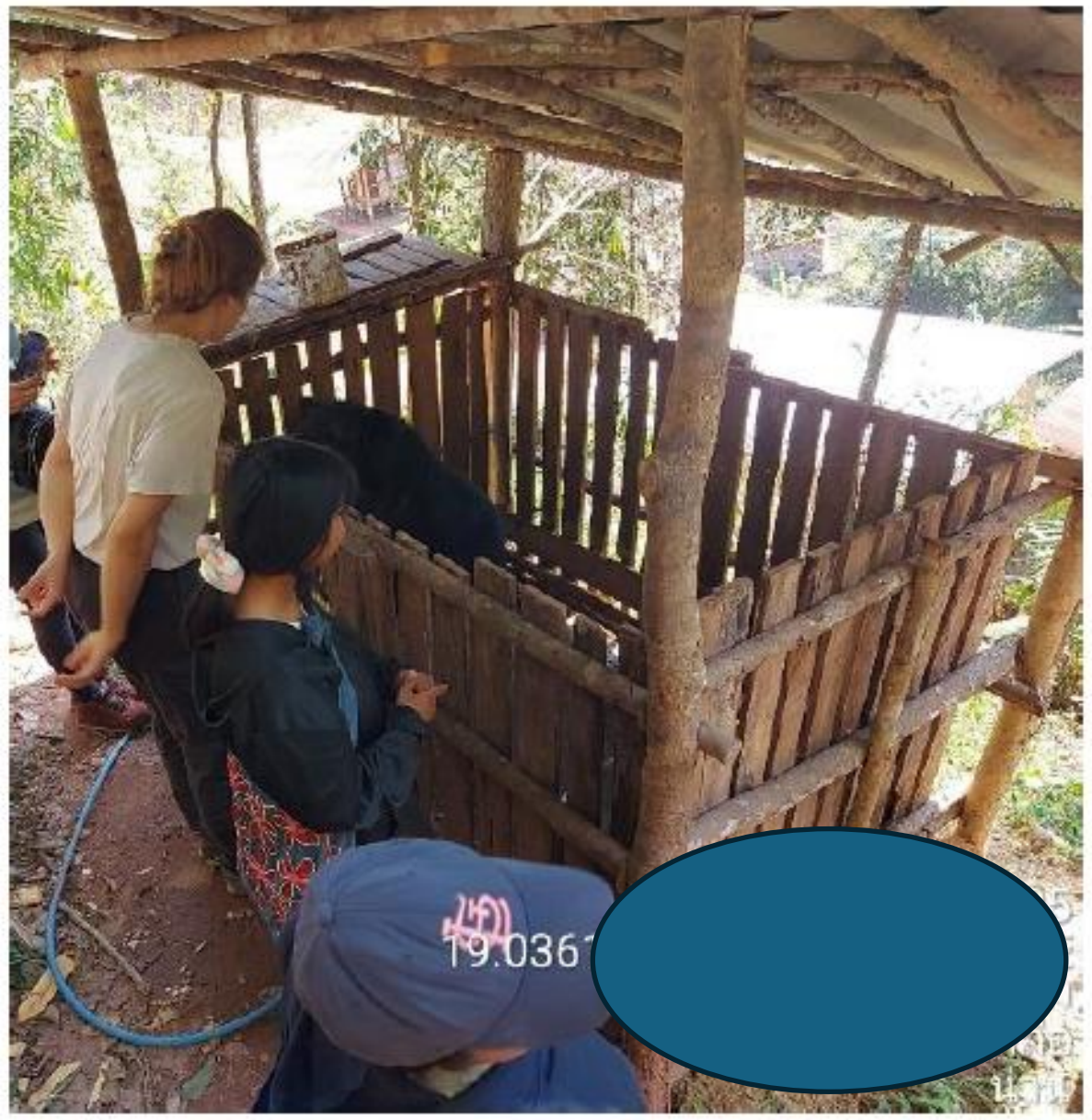
The pigsty is located behind the house
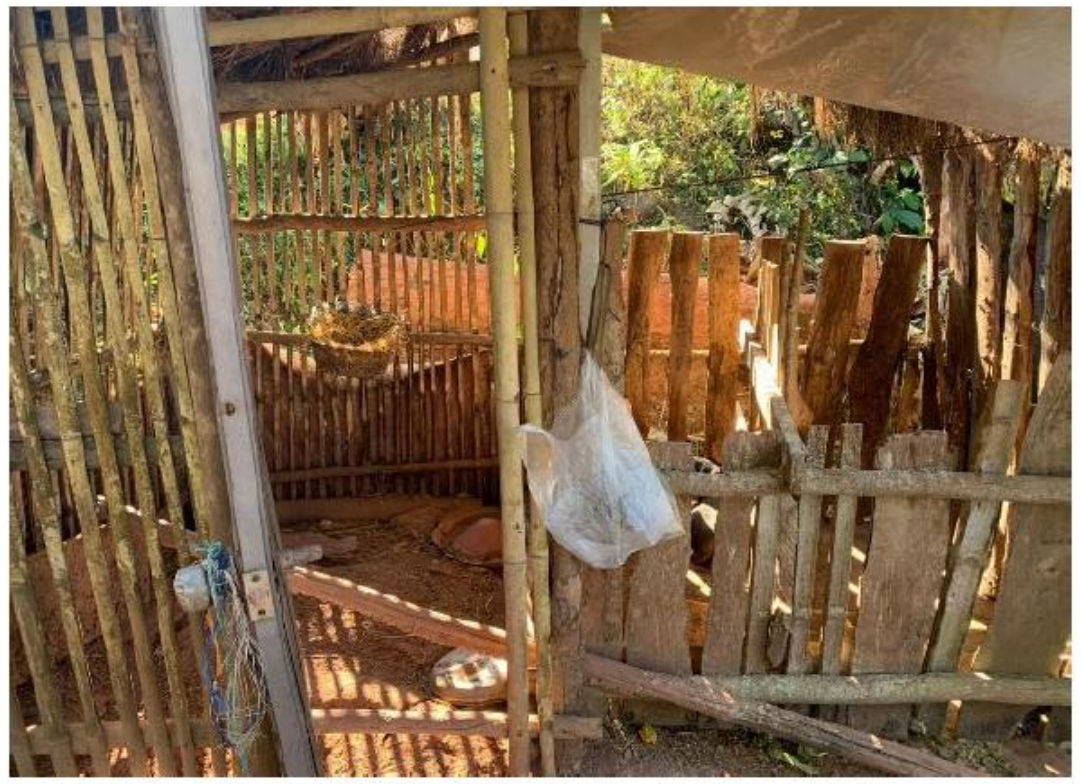 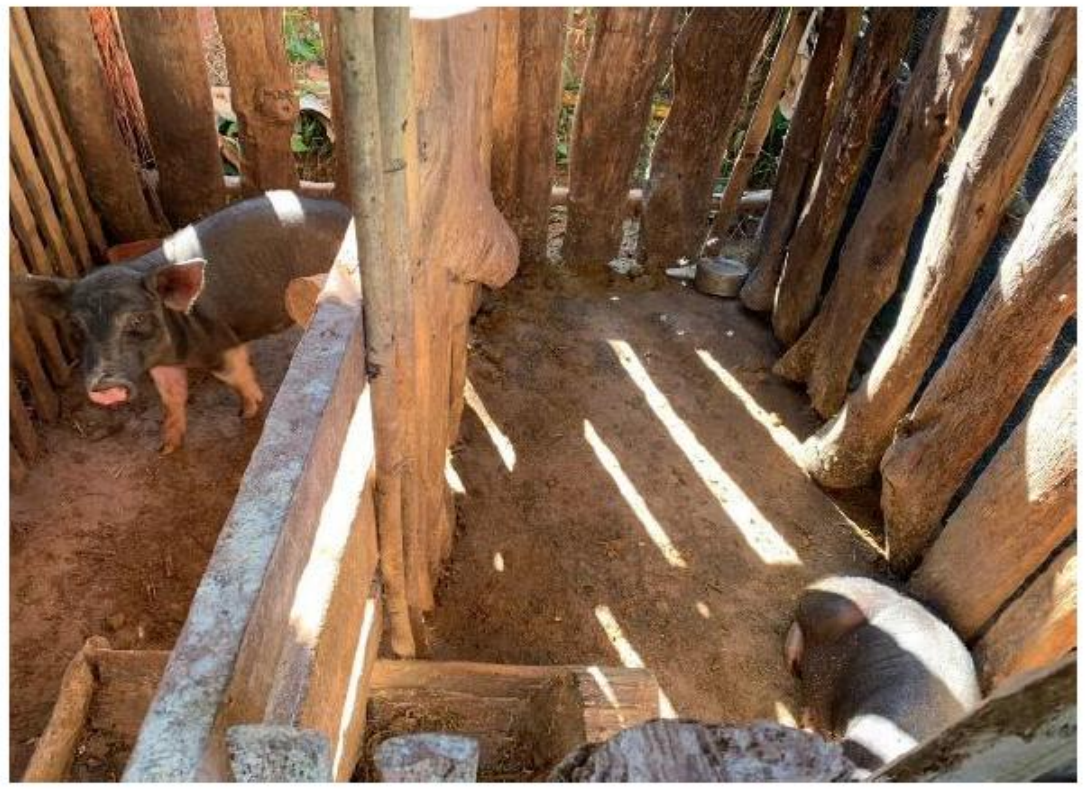

The interviewee has two pigs, and the pigsty is located behind the house. The pigs usually eat minced banana blossom and leftover food from its owners.</td>
<td></td>
</tr>
</table>

Pets	Well, we tell young kids not to play with them because we're afraid their fur will get into their nose or something. If it's a cat or a dog, we don't let kids play with them. They might get sick or get an infection. That's why we don't let young kids play with dogs or cats. We strictly forbid it. Another reason is that they might touch the animals and not wash their hands. Adults usually don't play with them either. We just give them food in a bowl. (Women, 49)		
Taking care of sick farm animals	We can hear their voices when they [fighting cocks] get sick. We won't let them leave the house or touch it or get near it.<br>[…] when they crow, it sounds like a person with a cold. Then, we give them medicine by injection.<br>They do in the winter and rainy season, because it's cold.<br>[…] We prepare the medicine from May to January because it's winter and the rainy season. The weather is humid during that time. We have to protect them from getting diseases.		

	[…] we are concerned that it will, which is why we have to prevent it. There has been COVID-19 before, we learn about that. We have to prevent it because we're living in a community.<br>[…] and it helps. I think it's like a person getting medicine.<br>[…] we always do because we want to prevent getting diseases as we live in a community.<br>[…] when we notice they're getting sick, we cover them because the weather is cold and take care of them as if they were a person. (Women, 46)<br><br>Since these are our own animals, and if they get sick, we can easily treat them in our own orchard. In the past, there was an outbreak of foot-and-mouth disease among cattle near the Nan River, but we were able to contain it in that area. […] it didn't affect us. I keep a close eye on my animals, so there have been no problems. […] No, never [did I vaccinate my animals]. I let them live naturally. […] I let my cows roam freely in the forest. However, if they get injured, I'll treat them with medicine. They die [id they		

	get sick]. […] and then I bury them. (Man, 41)<br><br>KThey had to bring them to the farm, and they reported it to the Department of Livestock Development. The department came and gave them vaccinations and treated them. They didn't bring the animals into the village, though. The affected areas stayed isolated. For example, mine was not inflected, but the cattle in the head-village assistant's farm over there were inflected, they stayed isolated and were not allowed to move anywhere else. They kept them on the farm, and the Department of Livestock Development came to vaccinate them there. (Man, 41)		
Recognizing sick animals	I rarely encounter sick ones. I'd get rid of it. Dig a hole and bury it. […] You can tell by its skin condition. It would have sores. […] It would have sores, kind of like a cut from a knife. Sometimes, it looks like the fur has been scraped off. […] like peeling the skin off. […], it would have small patches with fur peeling off. […] I mostly don't see it in birds. I usually see it in rats. (Man, 55, Hunter)		

	And the one that had rabies—people were certain it was rabies. It bit someone too. My grandma told someone to shoot it immediately. We had to chase it down because it ran into the forest. When a dog has rabies, the signs are clear—tail down, tongue out, drooling, and it bites at random. If it bites another dog, that one gets infected too. […] If your dog gets bitten, you have to isolate it right away and watch for symptoms. If it shows signs, you have to put it down, or it'll keep spreading. […] In the past, the disease spread badly. Now if we detect it, we act immediately. […] If it's clearly infected, we have to shoot it, especially if it's still inside the village. If it runs into the forest, we go after it and take care of it there—bang!—done. Last year, there was one case. I used my own backhoe to dig a deep hole and bury it. (Man, 56)<br><br>I think they [animals] probably can [transmit diseases to humans]. Because sometimes when I burn rats, I see white spots on some of them. I see it in the rat's internal organs. There are white spots, so I throw those parts away and don't eat them.		

	[…] If I see it, I'll throw those parts away. […] Some people might not throw them away, but cook them thoroughly. It depends on the person. (Women, 50)		
Transmission of diseases or parasites from animals	There have been some cases [of scrub thyphus], but it's not common. If people go into the forest often, they might get it because there are ticks in the forest. (Women, 65)<br><br>Someone in the village got lost in the forest and came back sick with scrub typhus. He got bitten by ticks and fleas from animals. People still get it nowadays — both villagers and officials. Hunter (Man, 52)<br><br>It was during the rainy season, and there were many mosquitoes. They would bite people and spread the disease [dengue fever]. Mosquitoes have a short life cycle; they can reproduce very quickly. If someone in my household didn't sleep under a mosquito		

	net, they would get sick within a few days because we live close together. […] it was serious at first. The village health volunteers would come to inspect every week. If they found any mosquito larvae, you'd get fined. It was a village rule to prevent dengue outbreaks. (Man, 41)<br><br>We have to eliminate mosquito breeding grounds first. If there are no mosquito eggs, there won't be any mosquitoes, and there won't be any disease. We have to take responsibility ourselves, even without being told. If we see standing water, we have to cover it or put sand granules in it. In my area of responsibility, I emphasize that if I find any mosquito larvae during inspection, they have to be eliminated.<br><br>(Women, 50)<br><br>last year, our village had a dengue outbreak, with over ten cases. Since our village		

	had a high number of cases, we had to distribute larvicide and Abate sand granules to households and actively eliminate mosquito breeding grounds every day. VHVs [village health volunteers] had to control it, or the outbreak would have worsened. (Women, 49, VHV)<br><br>And also intestinal worms. […] Yes, there are. If there is a doctor, they will bring medicine for the VHVs to distribute to those who have it. […] Last year there were about 10 people. But usually, they will give everyone two pills to prevent it. […] Mostly, they are mostly from raw fish, vegetables. Some people will have liver flukes, mostly liver flukes. It comes from fish, from, from vegetables, fresh vegetables, things like that. (Women, 36)		
Animal vaccination	it's from the SAO [Subdistrict Administrative Organization], they provide free rabies vaccinations every year. […] They come once a year to vaccinate the dogs.		

	[…] Usually, we take our dogs to the village hall, and the SAO team comes there to vaccinate them. They send us a form beforehand to ask how many dogs we have and their gender. […] I usually do it myself. It's not difficult; you just pinch the skin and inject the vaccine. (Man, 41)<br><br>Dogs and cats receive vaccinations from the livestock department, which works with the Subdistrict Administrative Organization to provide vaccines. […] The Subdistrict Administrative Organization allocates a budget to purchase vaccines, and officials visit the area to vaccinate pets like dogs and cats. (Man, 52)<br><br>If there's an [avian flu] outbreak, or if I hear that there might be one, I'll vaccinate our chickens. Even if I'm not sure if they'll get sick, I'll vaccinate them as a precaution. […] it's better to be safe than sorry. (Man, 41)		
Handling of disease outbreaks	I think it's possible [for bird flu to transmit to humans] because the virus in the air could drift towards us, and we could inhale it. That's my opinion. We need to take precautions and we should advise others that if they find dead birds or chickens, they		

	shouldn't touch them without gloves. They should use plastic bags to carefully pick them up, and then burn them. I think that's about it. Burying them probably won't work; they have to be burned. We need to spread the word to the villagers and let everyone know. […] And tell them that if they find any dead birds or chickens, they should immediately inform the village leaders or the village committee. They shouldn't touch them. (Women, 50)<br><br>If that happened, we would need to notify The District Public Health Officer at The District Public Health Office and The Health Officer to come and check for fevers and other symptoms, then refer anyone who needs medical attention to the hospital. People who have eaten raw pork should change their habits and start eating it cooked instead. This would help prevent diseases. (Women, 49, Head of VHVs)<br><br>It spreads from people who come from other villages, carrying the virus. When they're bitten by mosquitoes, the mosquitoes become infected, and then they spread	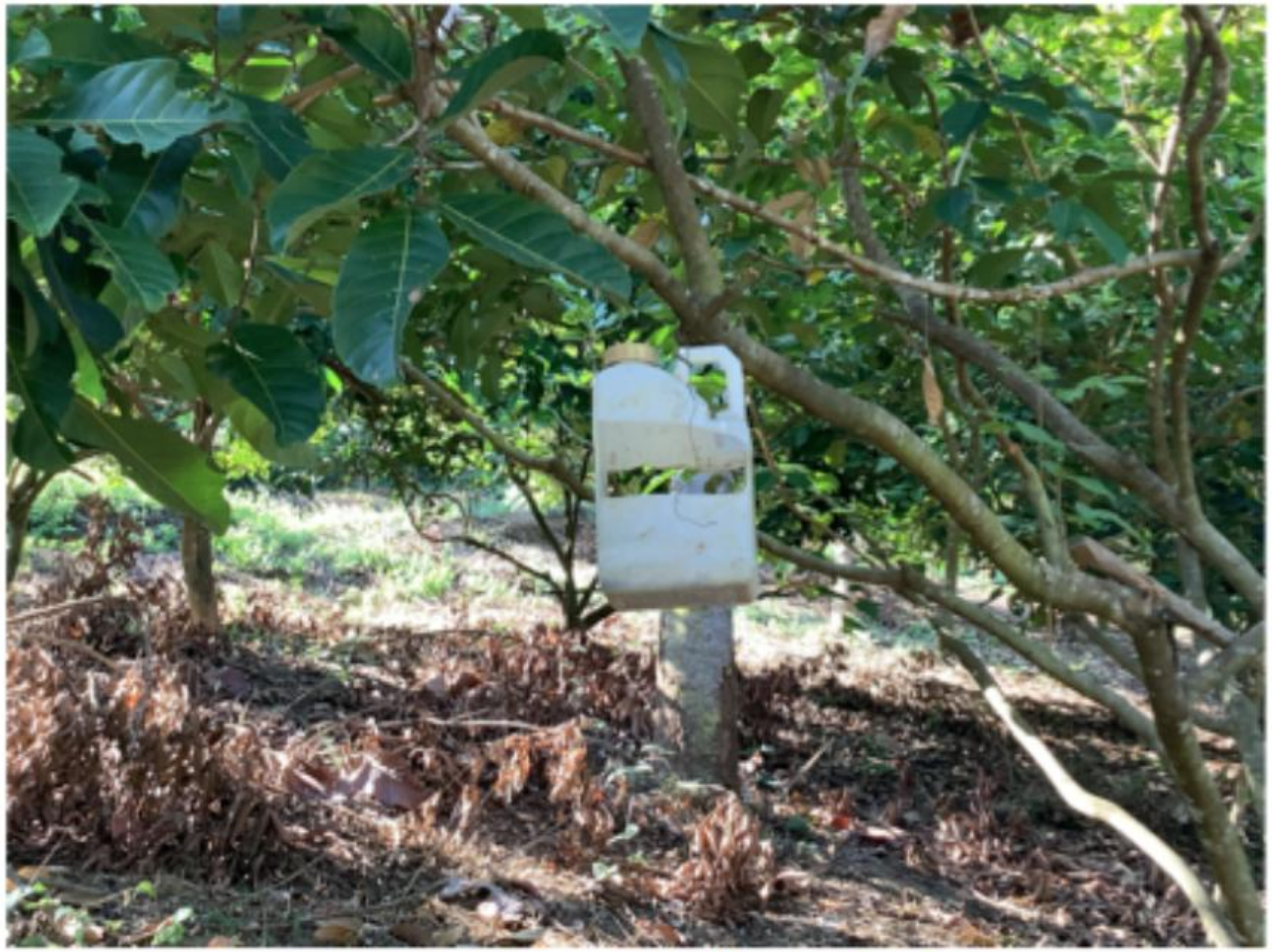<br>moth trap (ใช้กากน้ำตาลล่อ)	

	the disease further. I had to take action by launching a campaign to control and eliminate the breeding grounds for Aedes mosquitoes. I asked the village health volunteers (VHV) to help destroy the mosquito larvae by distributing temephos granules to the villagers. We also conducted field visits twice a week to prevent mosquito breeding. I need to report to my family and household members first, then consult with the sub-district headman to announce the news over the public loudspeakers. (Women, 49, Head of VHVs)<br><br>I've heard about it. There were two cattle that weren't properly cared for, so the disease spread to other people's cattle. Eventually, the infected cattle died, and they had to be buried. […] We had to buy vaccines and inject them into our cattle. […]The infected cattle had blisters all over their bodies. […] and it spread to nearby cattle. Some died, so The Department of Livestock Development provided medicine to sprinkle over the dead cattle. […] and our cattle didn't get sick because we took preventive	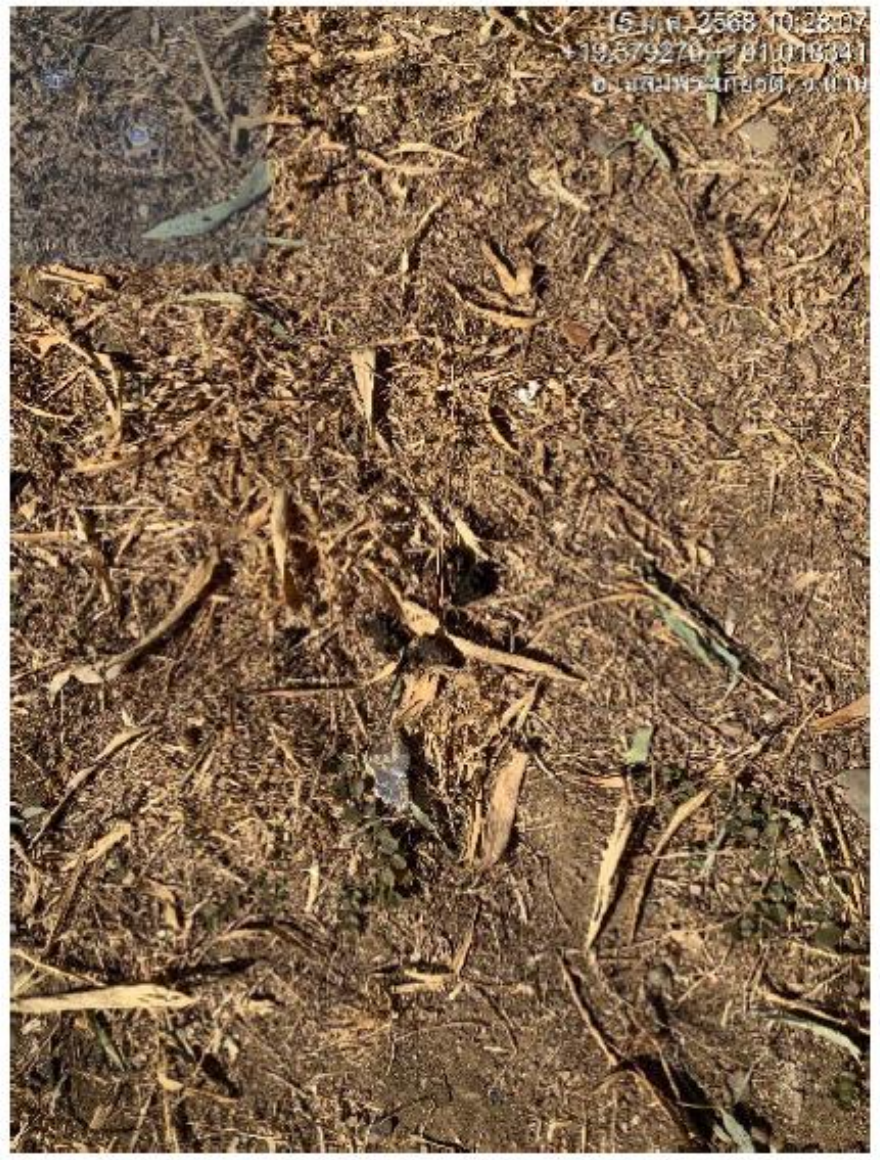<br>**7) Dog's Feces**<br>To my observation, a lot of dog's feces can be found spreading on the streets and drainages in the village, and most of households seem to have their own dogs.	

	measures. […] the disease spread to others, so people quickly vaccinated their cattle, and the outbreak was contained. (Women, 43)		
Dead animals	Mostly, we have problems with stray dogs from other places biting our animals. And it's not like they bite and eat them. They bite and just leave them there. […] It's very sad. Pigs too. […] They're not small pigs, even big pigs, they also bite and leave them there. It's heartbreaking to see that. But other people tell me to just take them and make grilled pork, but I refuse. I feel sorry for them, so I bury them instead. (Women, 50)<br><br>Yes, there were [dead animals during a disease outbreak]. [We] burn them. (Man, 41)<br><br>In our village, many animals died. It was around 4-5 years ago. In the area of [x], there were a lot of deaths. […] we buried them. […] we don't eat them. […] The animal owner buries them. […] The government sometimes helps. We take pictures of the dead animals, then report them to the district office. The village headman reports it, and we get some compensation, though it's not a full amount.		

	[…] we dig it ourselves. Everyone helps, like 4-5 people with shovels. […] If we raise them in the forest and they die there, we bury them there. (Man, 72)<br><br>We bury it. About—if it's just a chicken, we bury it about almost a meter deep. […] We don't want it to spread. (Man, 72)		

Table 4. Human-Environment interface

Code	Quote	Image	SW Note
Land use shift	Shifting cultivation like before is no longer allowed. The authorities say it’s a form of unsustainable farming. They want us to plant permanent crops instead—like tamarind, rubber, longan, rambutan, those kinds of trees.<br>[…] The focus is on planting long-term crops—no moving plots around—so the land won’t be continuously encroached upon like it used to be. (Man, 56)<br><br>Like with water—honestly, I still have doubts. The theory says that planting trees will increase water and moisture. But what’s happening is that the big trees we’ve planted are blocking the flow. They hold all the water. The places that used to have flowing water are now completely dry. It’s true. Imagine a forest that’s overgrown with huge trees—it used to still have streams. But now, with all these new tree species, they soak up all the moisture. The water that used to flow	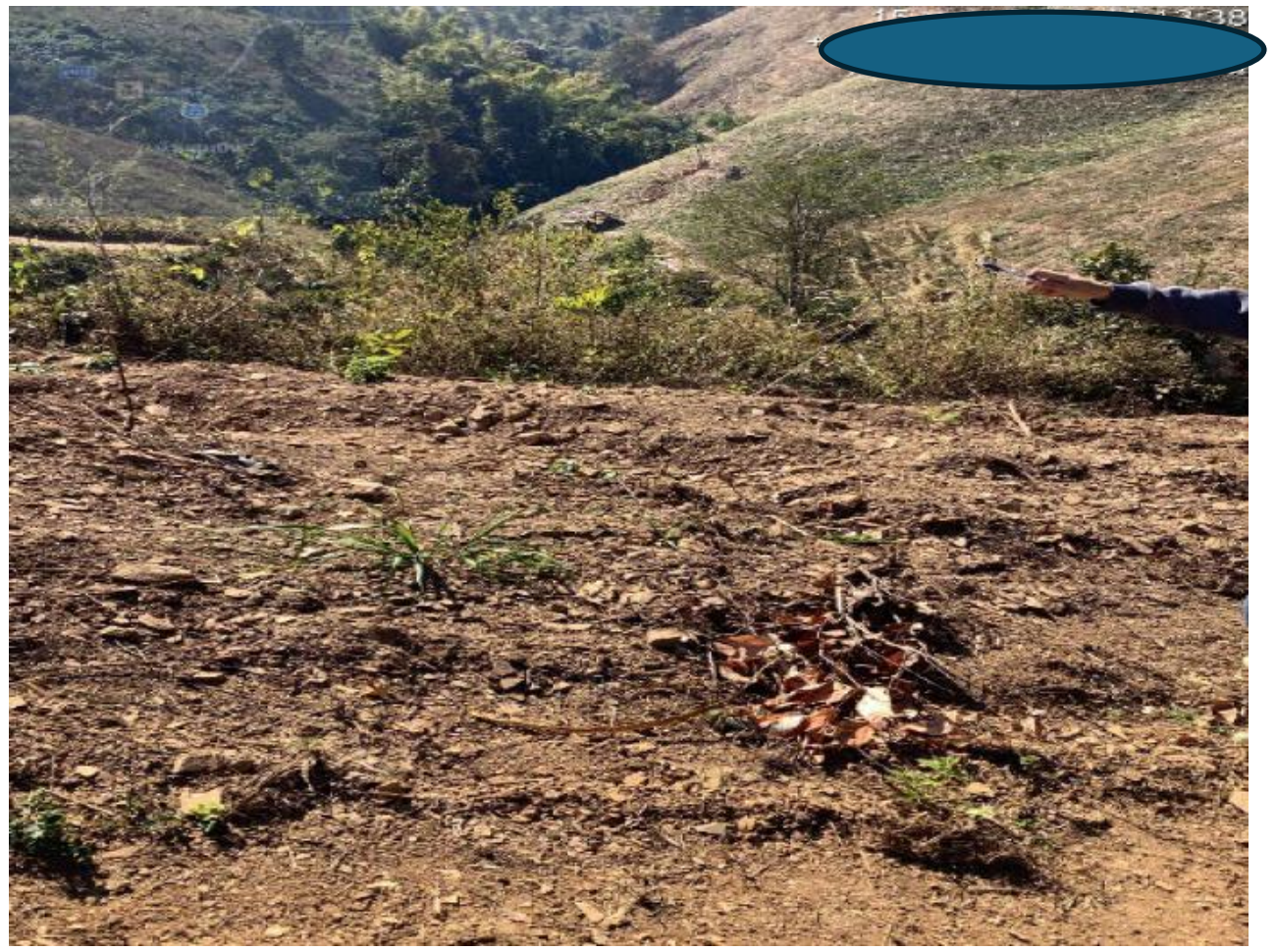<br>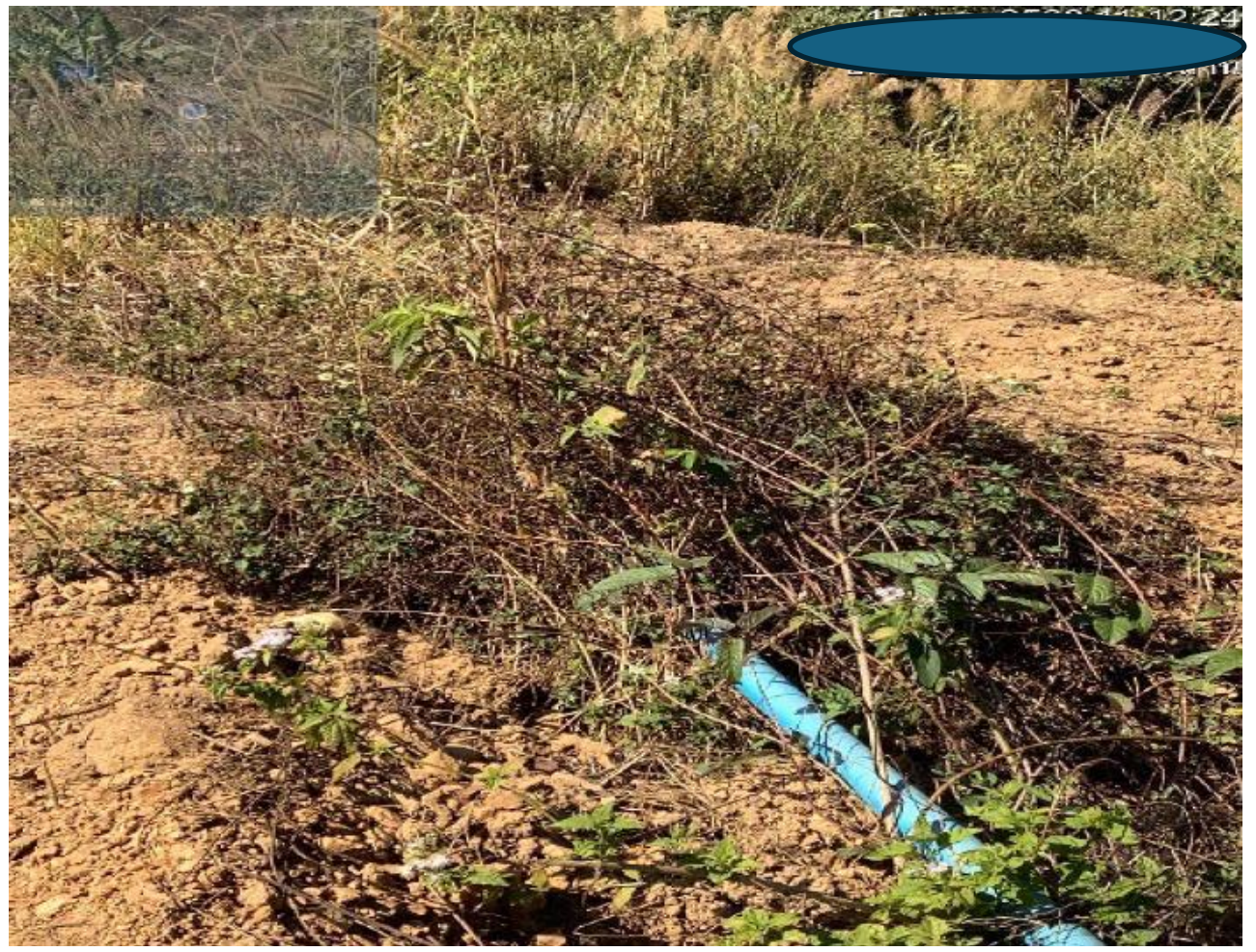	This land looks lack of water resource and drying while that green ridge belongs to head of subdistrict’s farm. In this dry land can be seen the old tracks of stream which connected the streams are being drained by pipes into the successful one.

	through is all gone, and the area becomes dry. […] The stream used to be strong. But now people have cleared the hills for farming, and once the high ground is bare, the water just disappears. (Man, 56)<br><br>The government has been promoting reforestation, especially the “three types of trees, four kinds of benefits” model. But when too many trees are planted, water ends up seeping underground instead of flowing where we can use it. So now, the forest looks green up top, but the land below is dry. […] Back in the day, we practiced shifting cultivation. We would clear a patch, grow rice or sugarcane, then leave it fallow and move on. Water still flowed then. But once the forest filled up and we weren’t allowed to clear or expand anymore, the water started disappearing. (Man, 56)	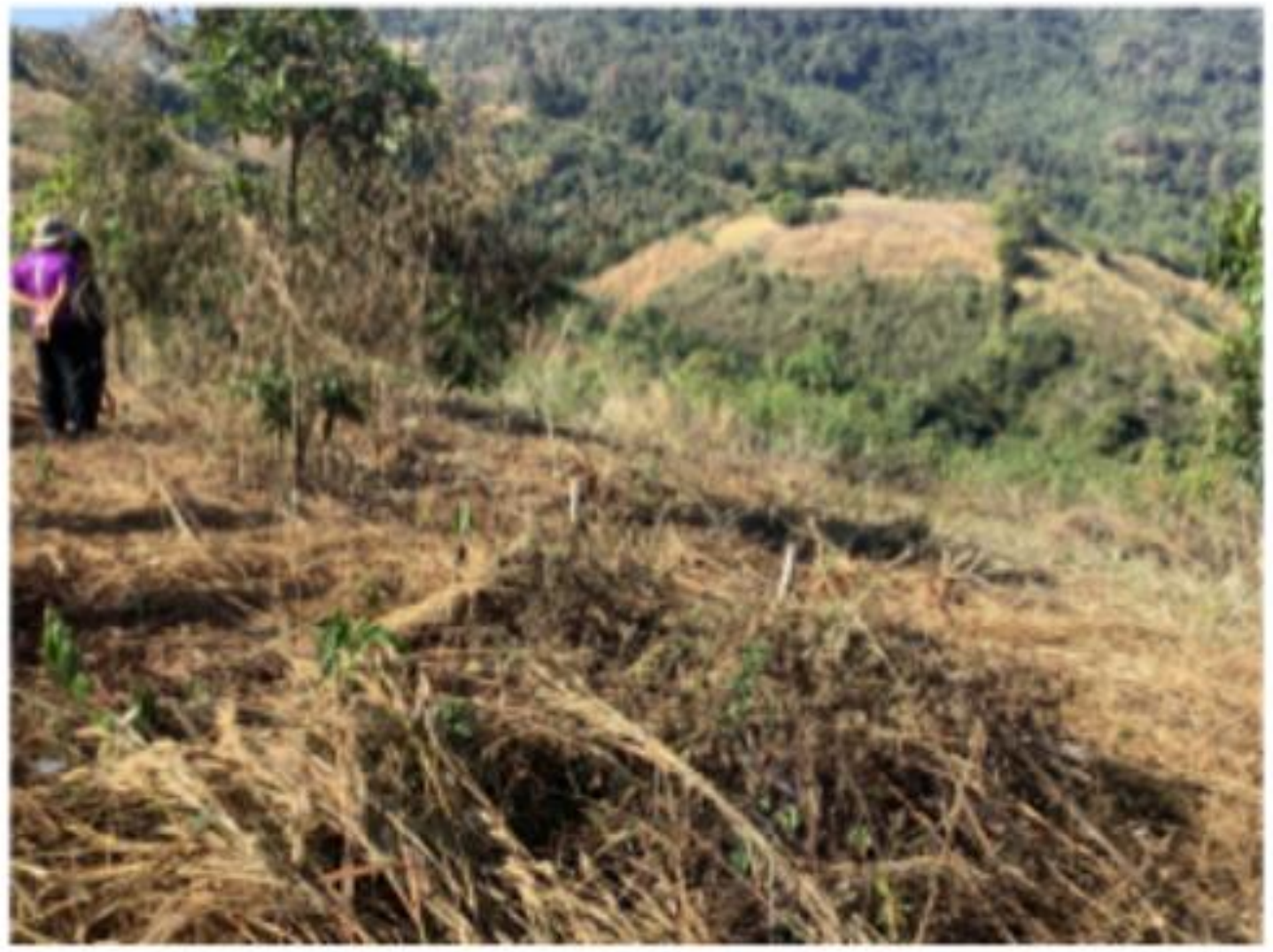<br>พื้นที่ ปลูก พืช ขั้นบันได	
Environmental pollution	It’s in Laos. *Hongsa* is a power plant. If it operates for 5 – 6 years, toxic substances can be released. […] it affects agricultural production, causing poor crop growth and making the soil acidic or alkaline. […] it [air pollution] could affect those		

	who already have health conditions. (Woman, 49)		
Field burning	Everyone has their own farmlands, including orchards and fields. Before burning, they're required to create firebreaks around their farmlands. […] Since before the government agencies would come in, the villagers had already been doing this themselves. Nowadays, there's a bit more monitoring to ensure the fire doesn't spread into the community or other people's plots. This is because farmlands are now more open and connected, with some villages even extending into the area of another village. People who own farmlands know where their borders end. They create firebreaks around those areas, and we look out for each other in certain spots. They use bamboo, splitting it into strips for sweeping, and then use knives to cut and pierce openings along the edges of their own plots. They clear the area, leaving a gap about one *wa* (two meters) from the spot they intend to burn. For example, if they plan to burn here, they'll create a cleared perimeter around it, leaving a safe space from the area to be burned. Yes, they clear the area like a road, leaving no leaves or anything that could		

	catch fire. […] we use all natural materials. (Women, 46)<br><br>Especially during the times when people are clearing fields or small garden plots. It's still allowed in small amounts because there are certain periods when burning is permitted.[…] but you have to check the season. Every year there's a strict no-burn period. The early burning practice—ching phao—is traditional local knowledge. (Man, 56)		
Pesticides	For the corn, I use chemical fertilizer, specifically urea 46. For cassava, I don't use fertilizer, but I do use glyphosate weed killer. I spray the glyphosate before planting the corn, but after planting, I don't add anything else. […] It's expensive. The fertilizer is quite costly. (Women, 43)	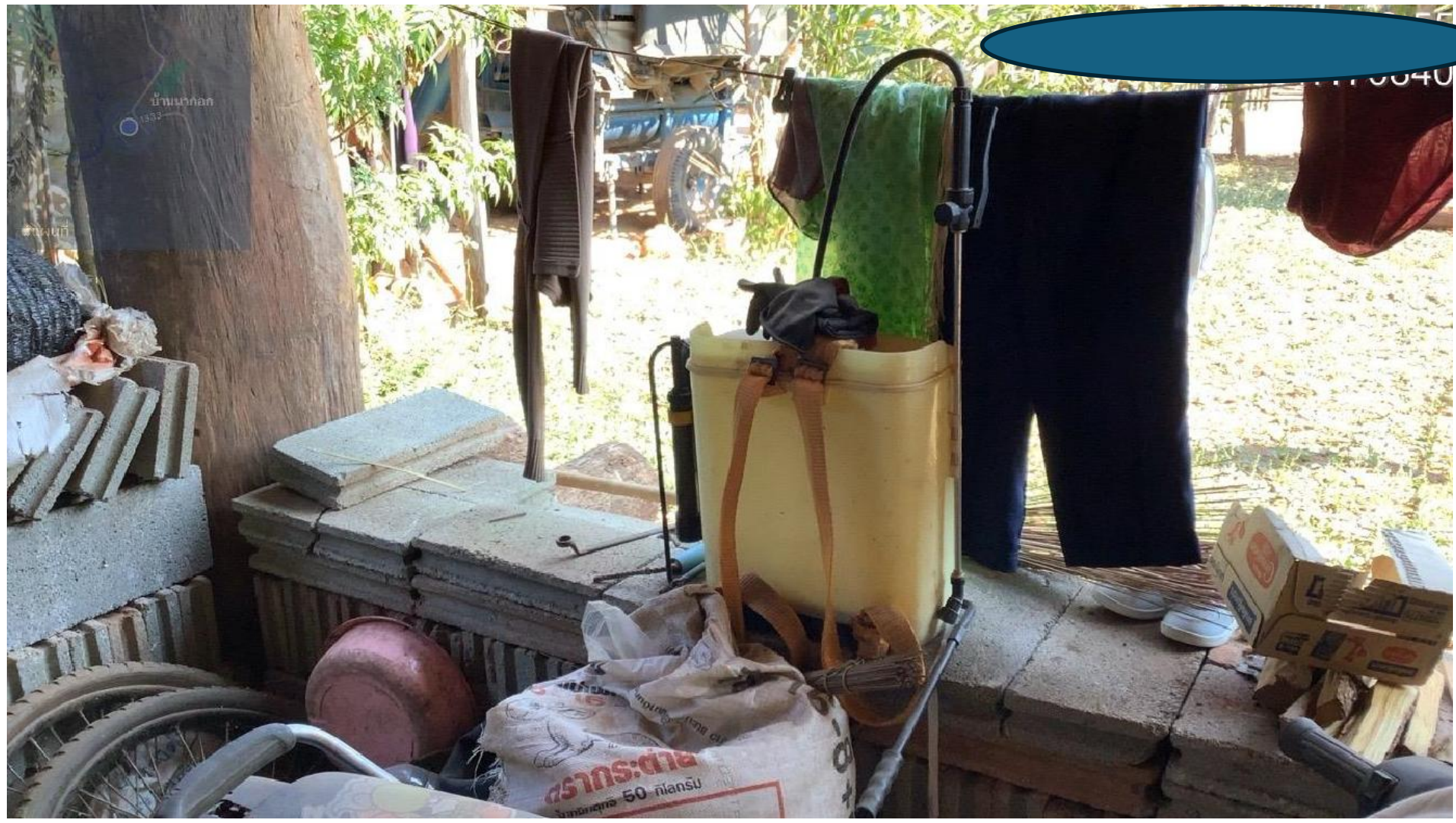	He and his wife use this hand sprayer contained with insecticide and herbicide to make earning from hired in other's agricultural sector. This is their more important tools to make a living comparing to weapon for hunting. Moreover, in the white bag under the sprayer is the

			chemical fertilizer (16-8-8) is provided by the corn farm owner who wants they to put for his this week.
Natural fertilizer	Pordor2 is a liquid organic fertilizer that comes in a bag. If it's solid compost, it's called PorDor1, but this is PorDor2, we use it to make bio-extract. […] For the fertilizer, we use ripe bananas and water. For each bag of PorDor2, we use 20 liters of water. We can also add other organic materials like shells. Since it's organic fertilizer, we can add almost anything.<br>[…] The best shells for making fertilizer are from golden apple snails. Golden apple snail shells make the best fertilizer. When we use it on plants, they grow faster. I usually mix the fertilizer with cow and pig manure.<br>[…] Even for my rambutans, I use cow manure. I just go stalls, ask for some manure, and put in my truck. Pig manure, I stored it in sacks. When the rainy season comes, I spread it around the base of the trees. […] When you taste a naturally ripe rambutans, it's sweet and crunchy, unlike those grown with chemicals.	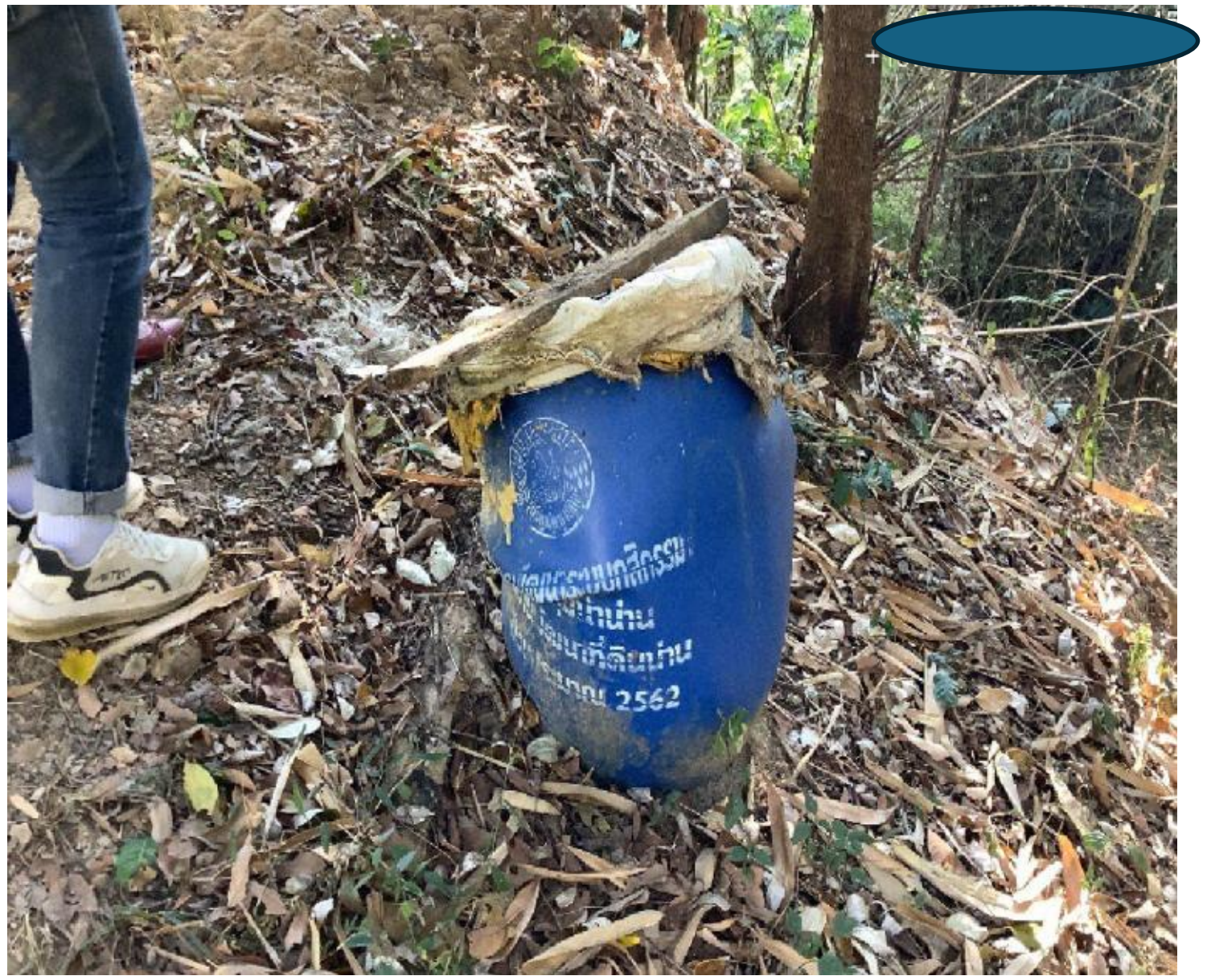	For every pilot farm, they are informed to use only organic fertilizer, so the organic fertilizer tank is provided them to mix their own by dry leaves and wet garbage like remaining food.

	This one is organic, completely natural. The soil here is also healthy and fertile. I let nature do its thing, I just apply cow manure. […] There are so many types of fertilizers. Some people prefer chemical fertilizers because they give quick results, but I prefer natural fertilizers because they last longer. For example, with rambutans, my friend uses chemical fertilizers and his produces don't look good. Also, it requires huge budget, like a thousand baht per sack for urea fertilizer. But with cow manure, I just need to collect it from my neighbors, doesn’t cost any money just only use physical power. And the fruits taste sweeter and crispier. (Man, 41)	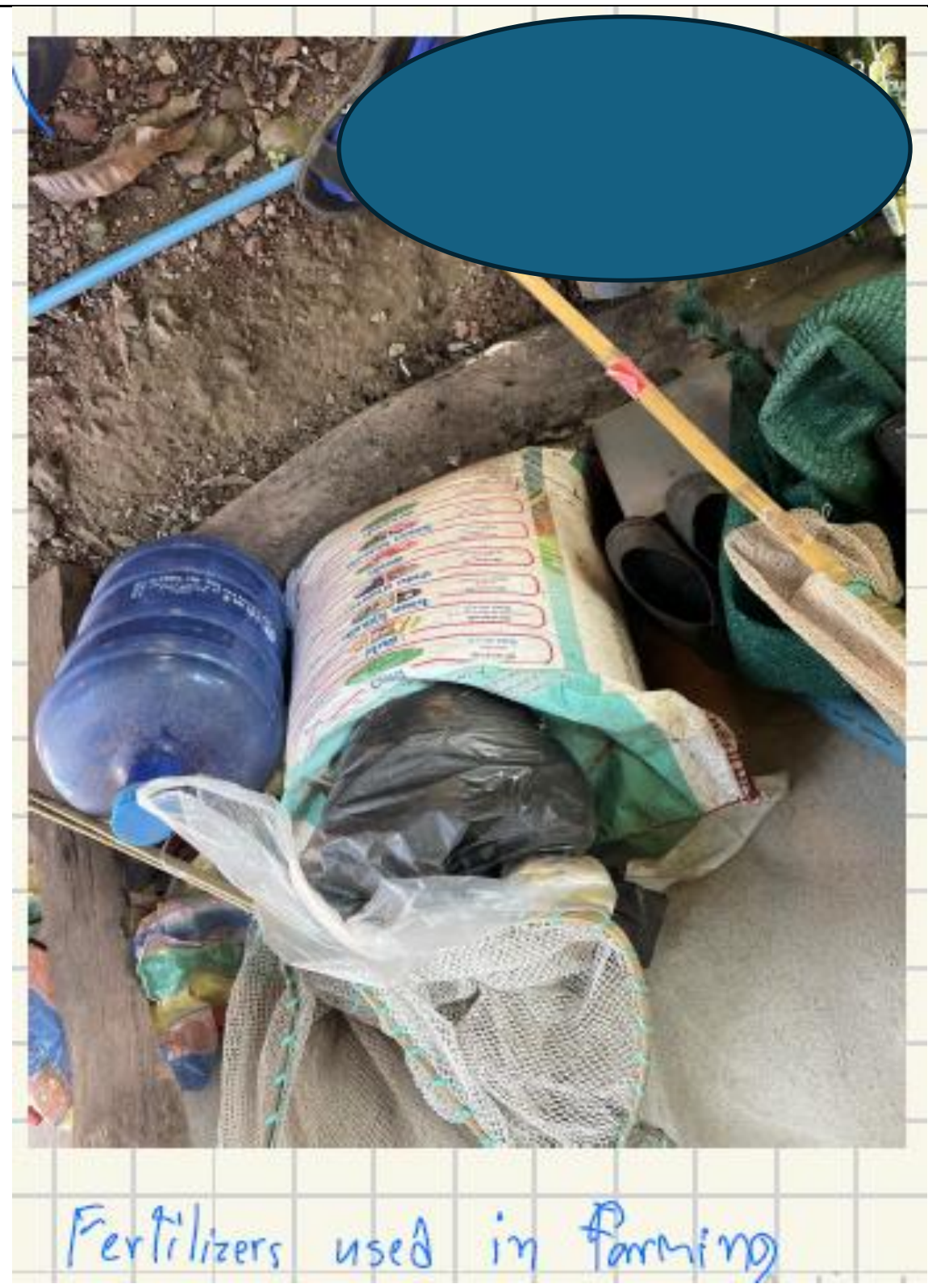		

Water scarcity	The main problem is water. We’re located on high ground with no reservoir. In the dry season, there’s not enough water. (Man, 56)<br><br>About 4-5 years ago, I used to raise ducks behind my house, about 50 meters away. But the ducks died because there was no water for them. So, I stopped raising them. (Women, 49)<br><br>The biggest problem in Chaloem Phra Kiat district is drinking water. In the past, we had to buy water, but now thanks to the installation of a borehole and a storage tank in the village, the situation has improved. However, we still need to buy water besides drinking water. A tank full of water costs around 250 to 300 baht for someone doesn't have a car. If you have car, you can go get the water at station below. Now, we also have a solar-powered water system provided by the sub-district administrative organization (SAO) last year. […] we take turns every three weeks. But it's not enough because we use water every day for washing dishes, doing laundry, etc. For my family of four, my mother-in-law, my wife, me, and my daughter,	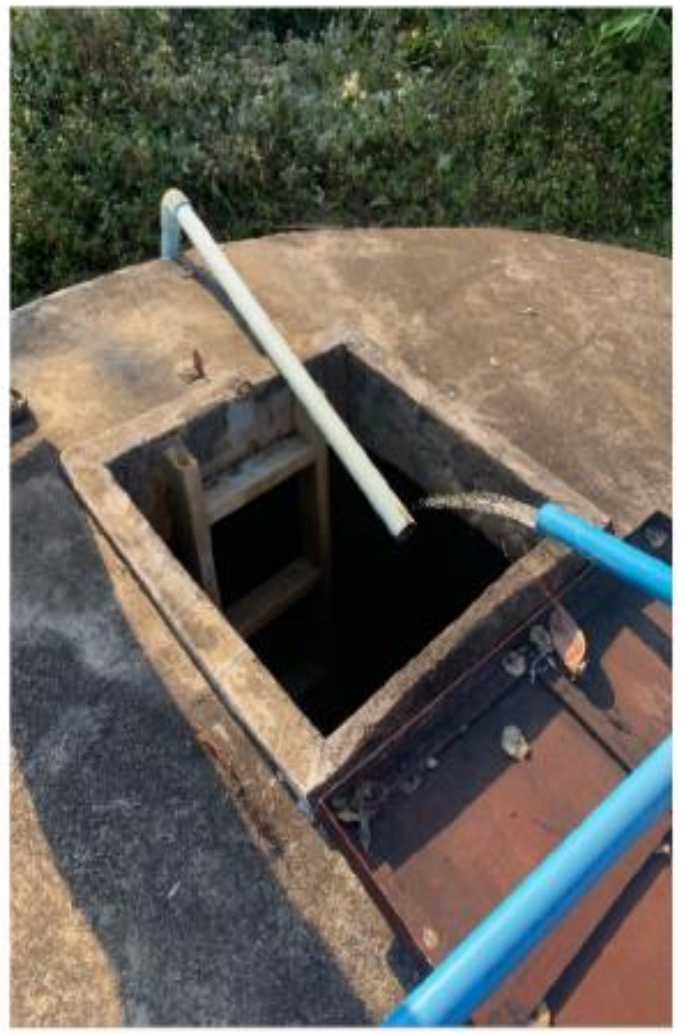 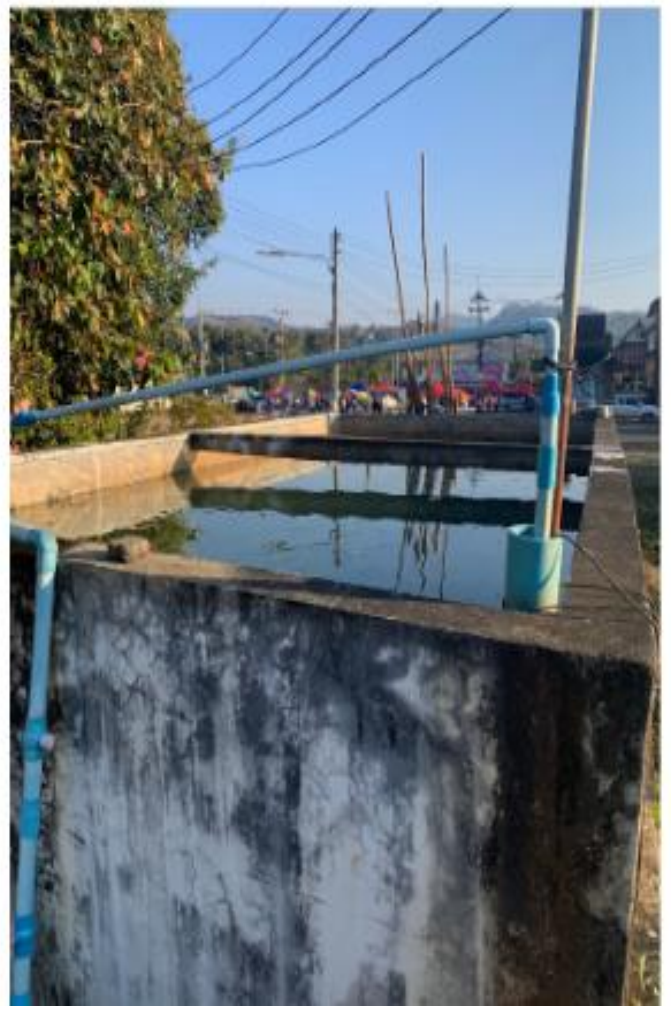 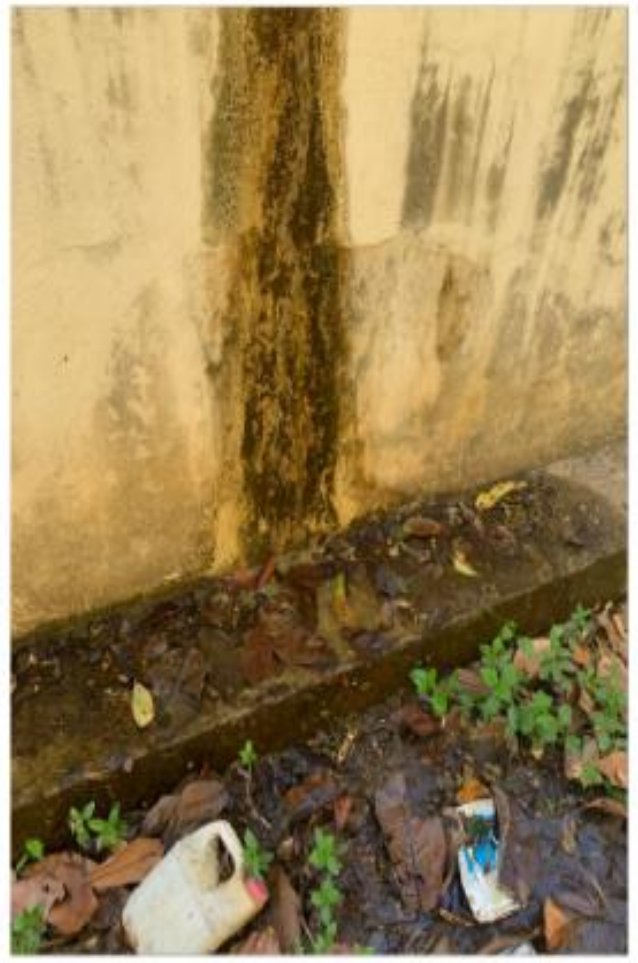<br>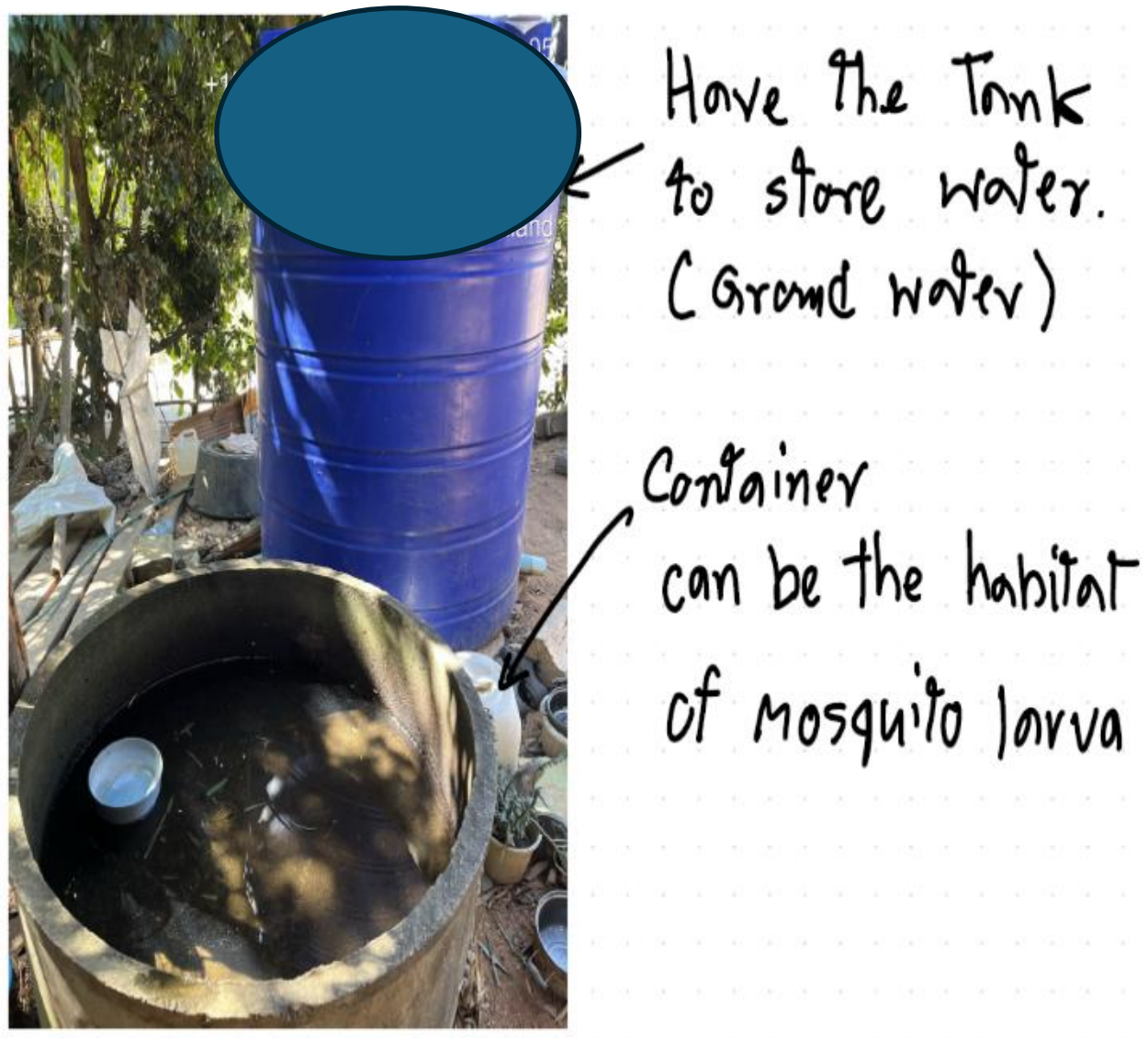<br>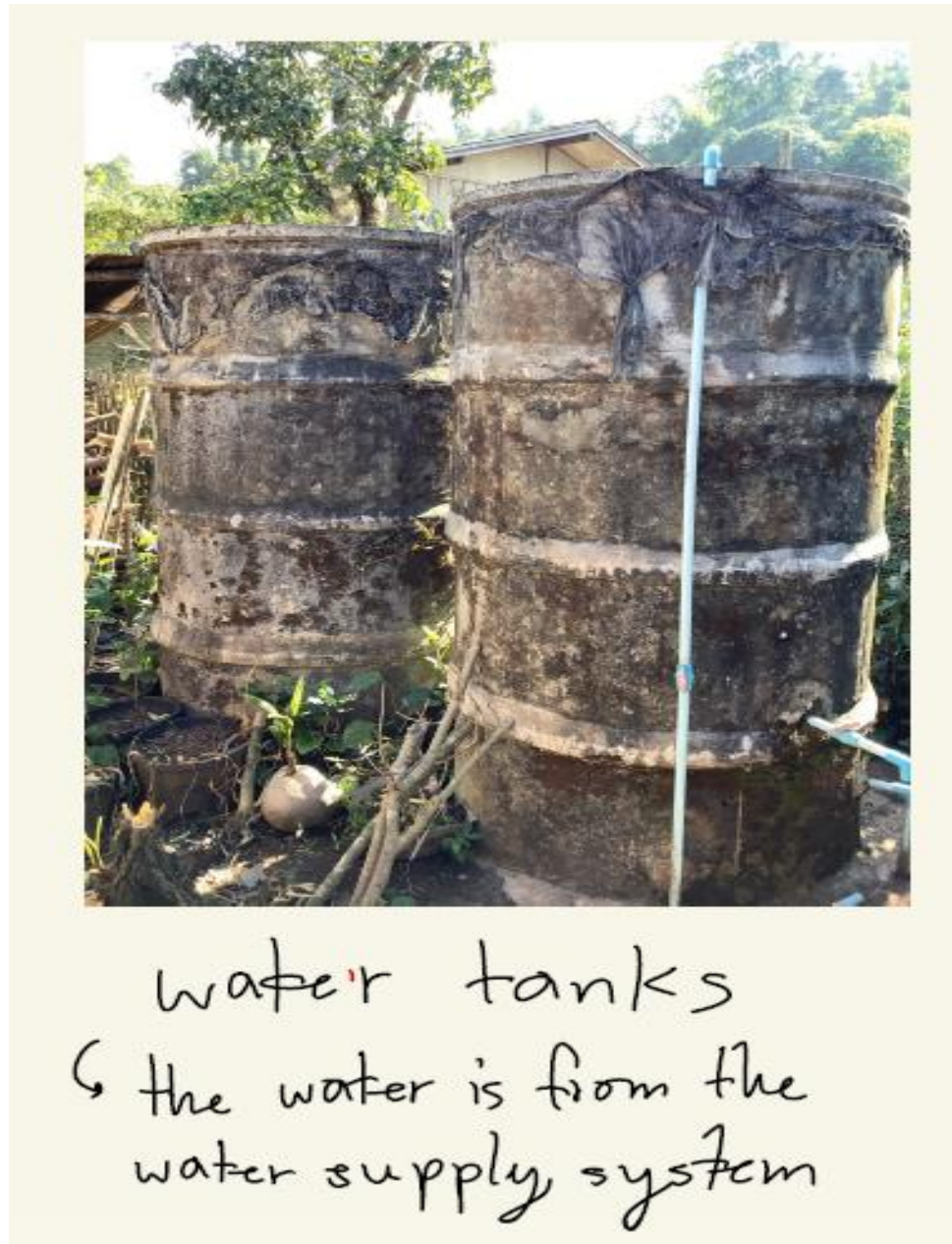	

	one 1,000-liter tank lasts about 3-4 days. (Man, 41)	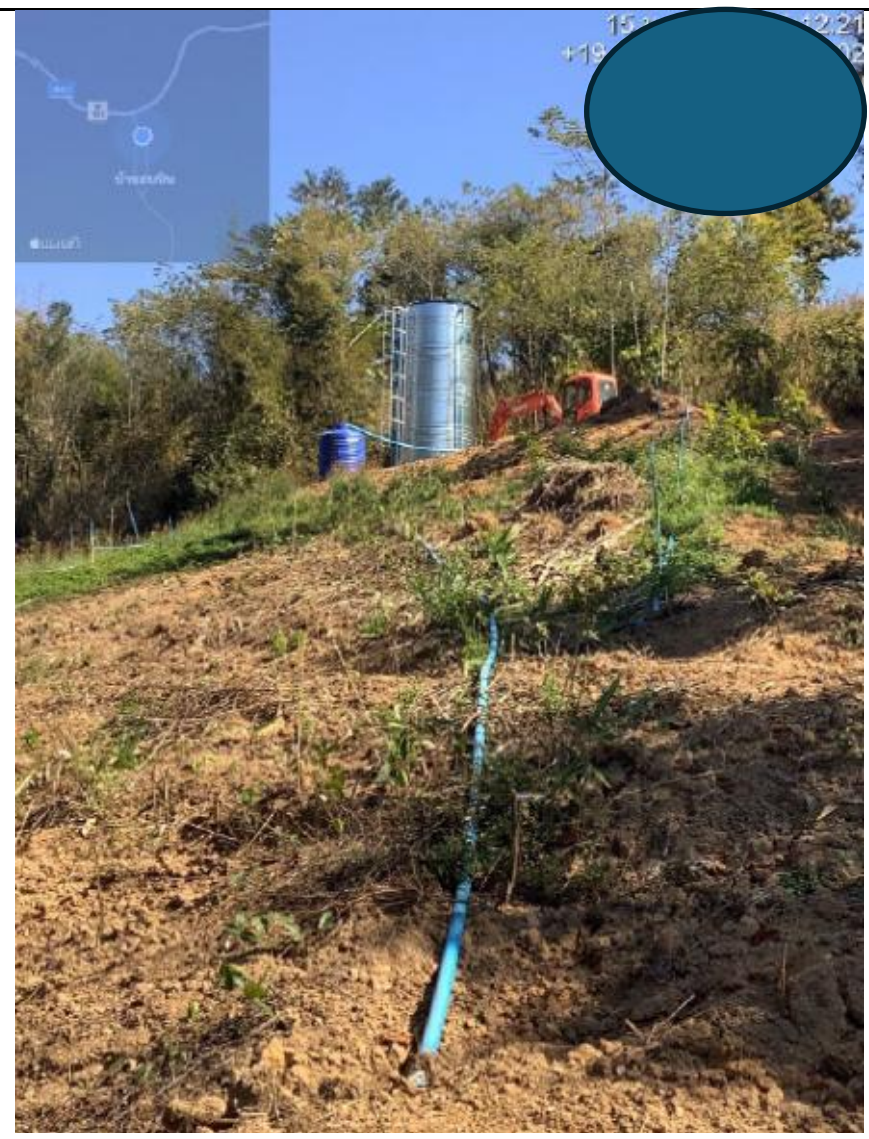 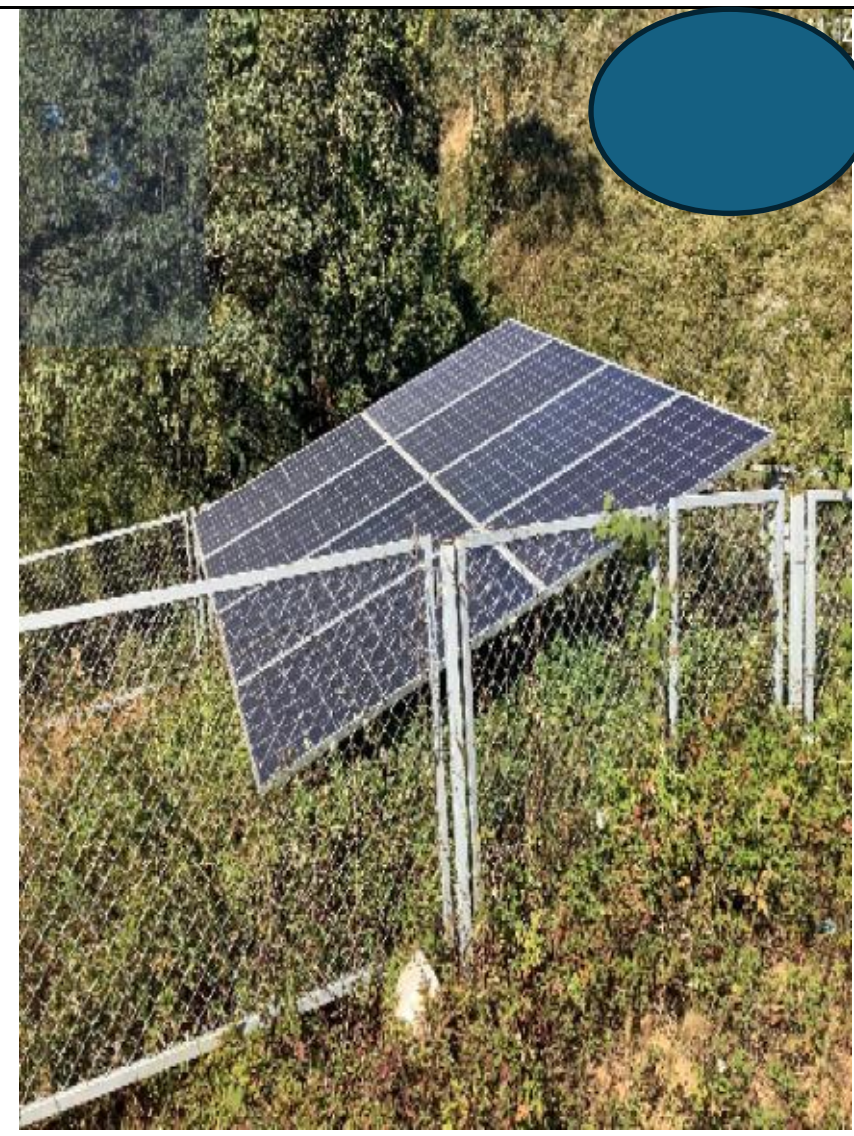 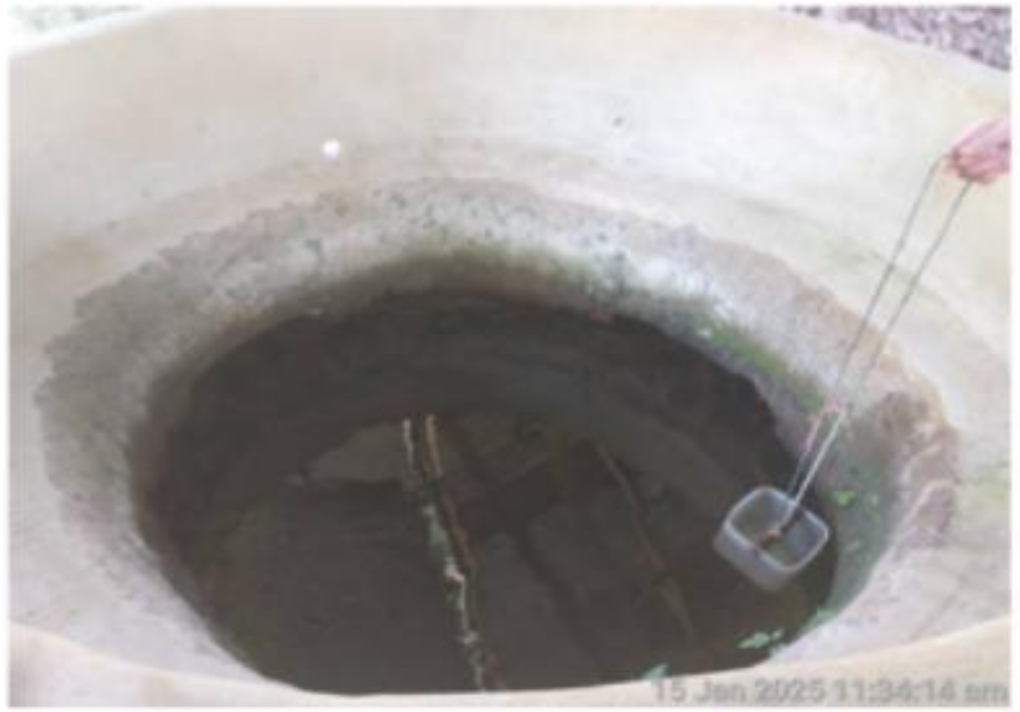 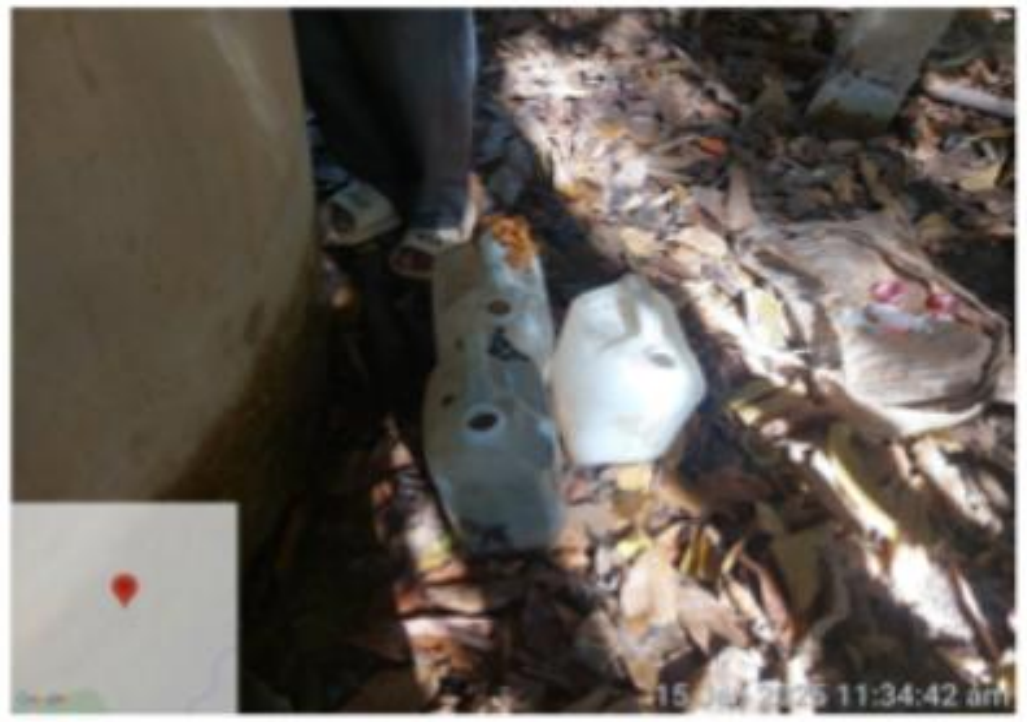<br>One of the villagers was taking water from the artisan well for her household usage. She shared that the spot is no longer prefered because there is another spot in the village to get underground water. She mentioned that people had to wait in line to take water.	
Waste disposal	At my place, since I have my own backhoe, I dug waste pits—two of them. […]The other pit is in the community		Garpage dump near the cow cage, a lot of poop

	forest—there were no big trees in that area, so I dug a pit about 15 by 15 meters. It's for the villagers to use. I don't let people dump or burn trash by the roadside. It has to be disposed of properly in those designated spots.[…] We have a committee go out to check if the pits are full. If they are, we burn the trash. Most of the trash is dry waste, so it's easy to burn. No problem. The pits are wide and nearly 3 meters deep. I've even built fences around them—to keep dogs from digging around, especially with things like styrofoam and plastic. So I put up fencing and a gate. Since we're next to the forest reserve, the Subdistrict Administrative Organization can't dig in the protected zone. But in the community area, we're allowed. […] I even built an access road so vehicles can go in and turn around easily. (Man, 56)<br><br>The village has a designated area in the forest for garbage disposal. When it gets too much, the villagers organize a cleanup and burn it. […] Usually, if I go to Huai Kon, I'll dispose of it at the sub-district's landfill. […] We	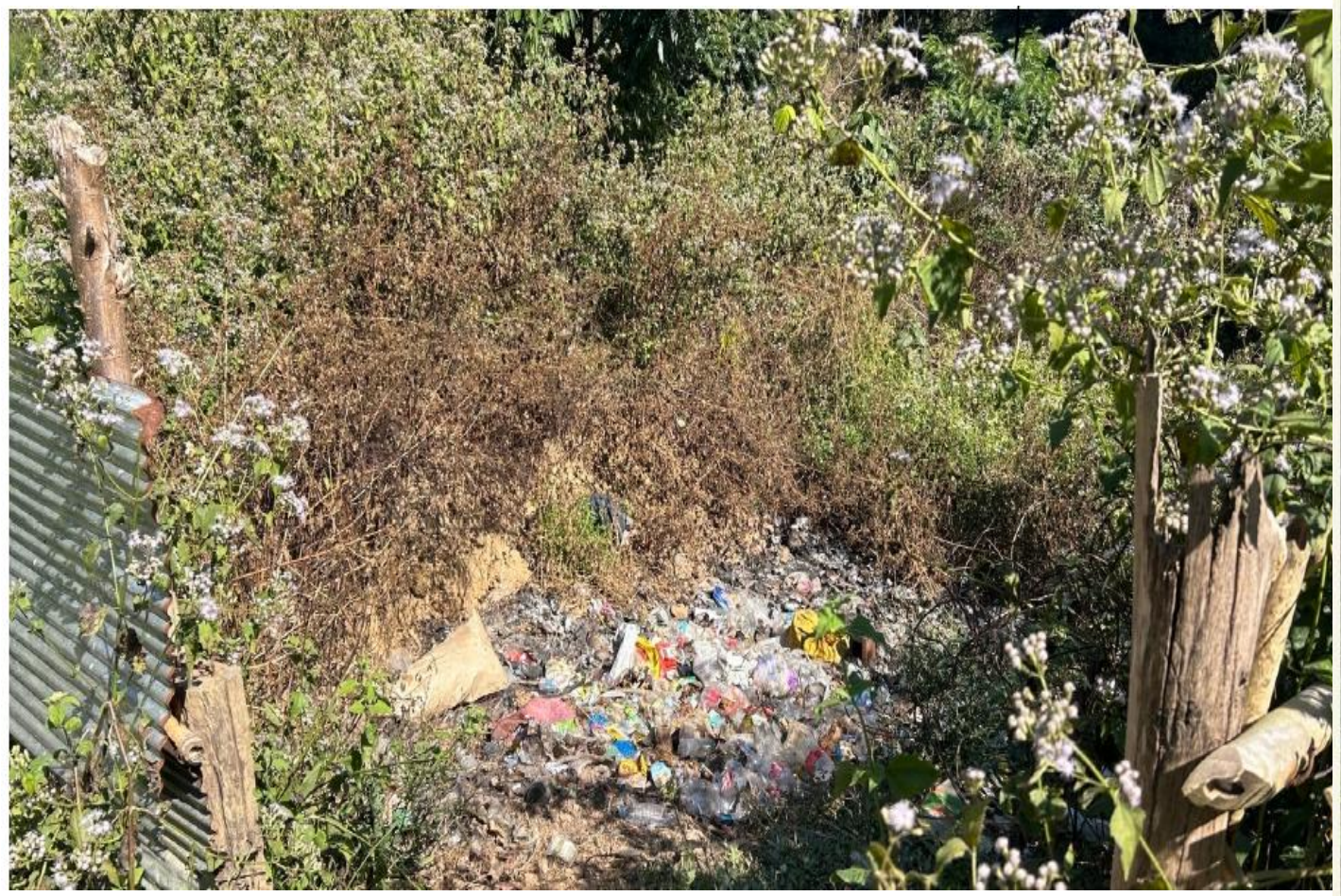 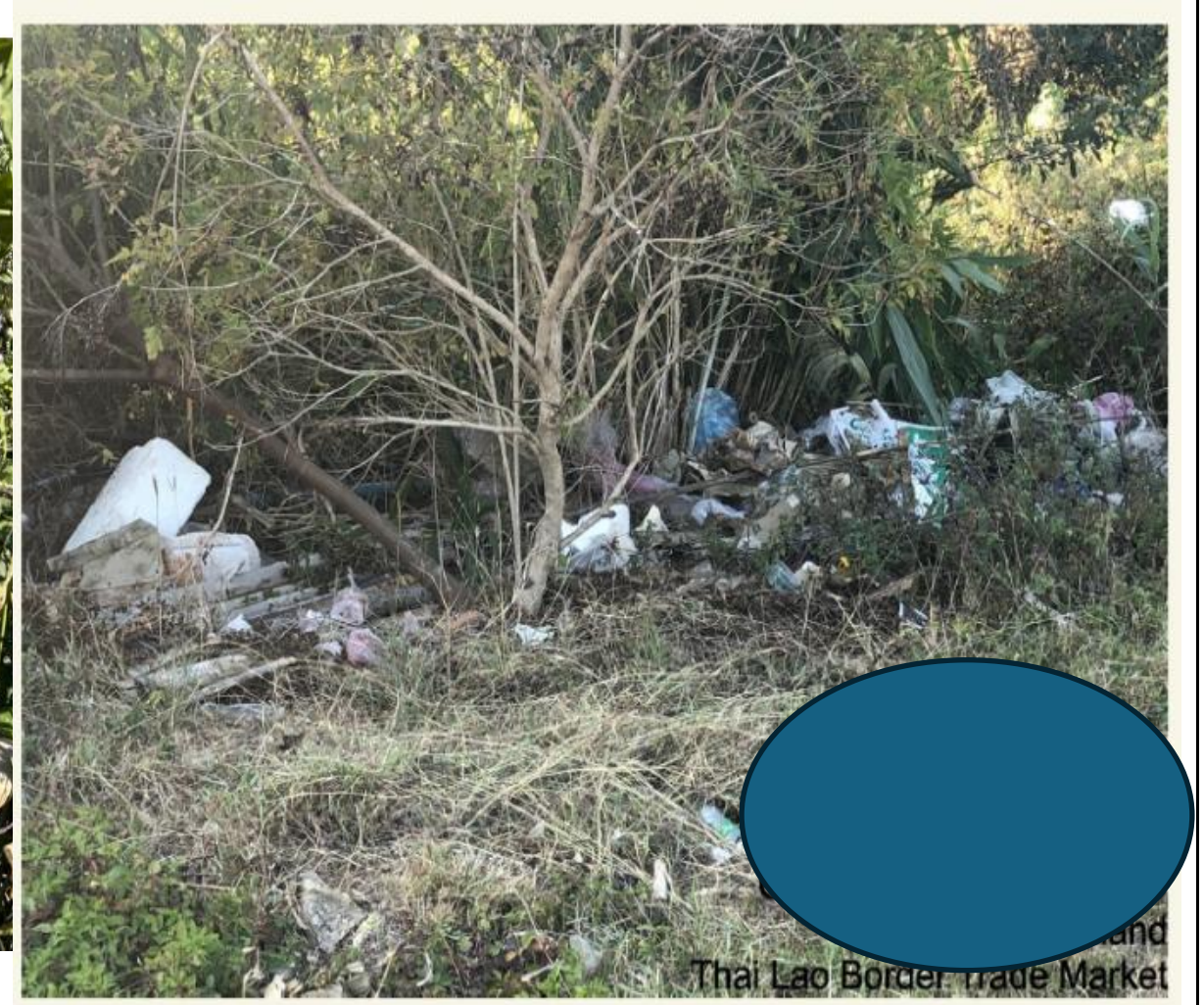<br>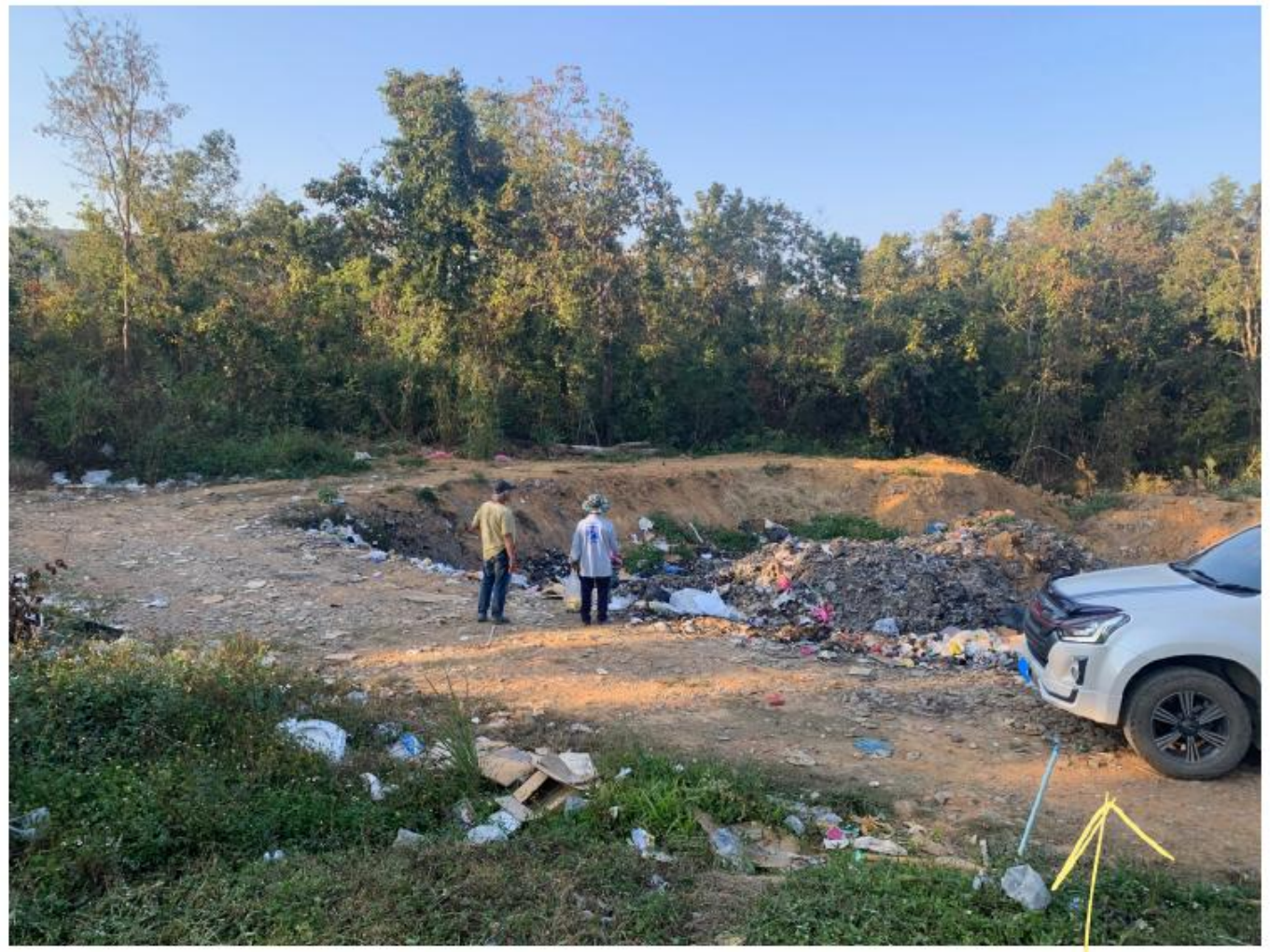<br>The waste landfill is under the subdistrict Administrative organization's control (SAO), provided to people in the area for waste dumping.	from the cows and dogs grabbing the garbage on the grassfield

	usually just burn it together. (Man, 41)	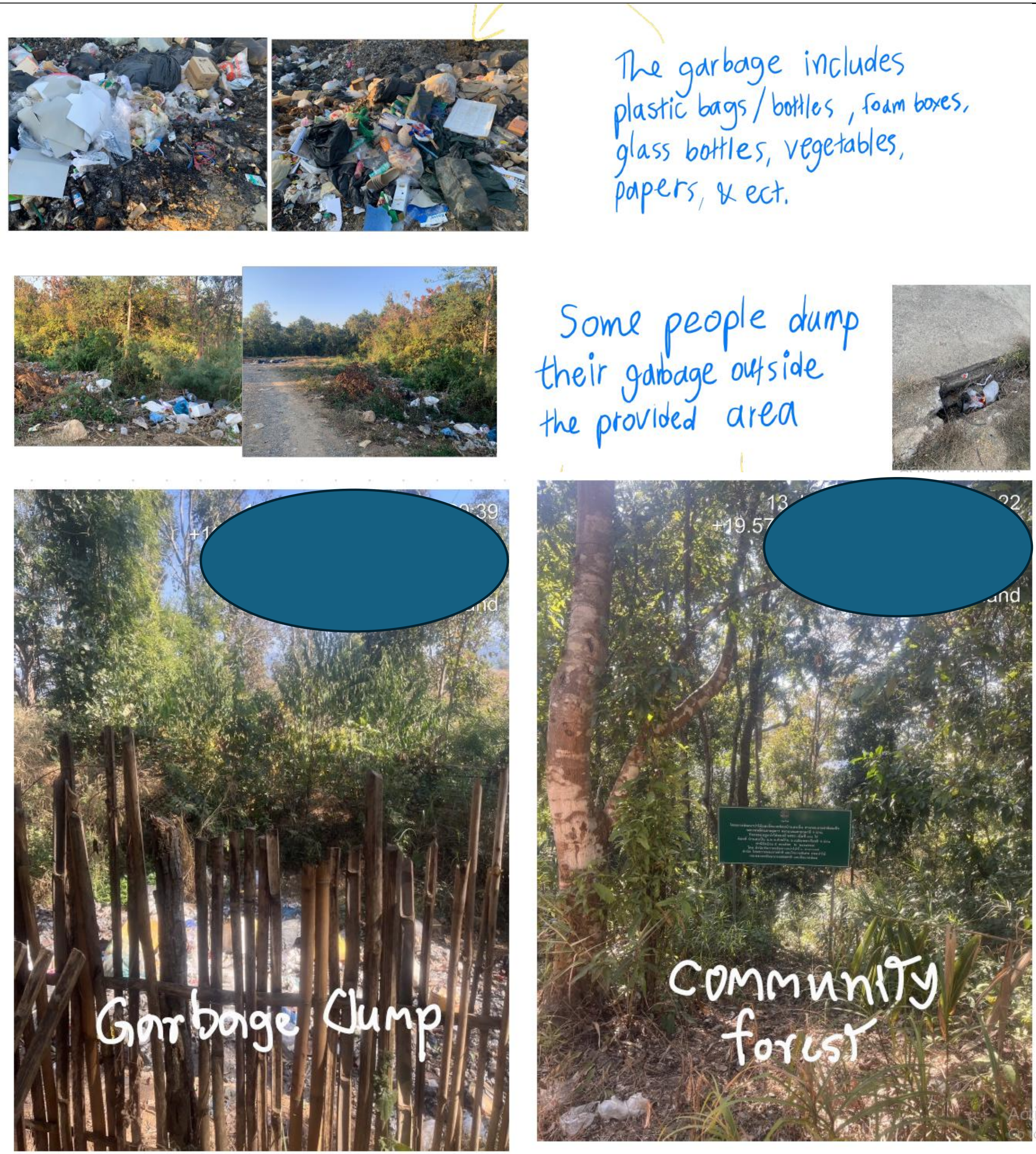	

Foraging in the forest	We still go into the forest to gather food. We gather mushrooms, and since we don't cut down trees, the forest authorities don't mind. We gather mushrooms, crabs, and fish. […] I go with female friends. About 4 - 5 people. […] We usually get enough to eat, like huak and shrimp. […] The amount is only enough for eat. […] Last year, we sold it. But this year, there's not enough to sell. (Women, 43)	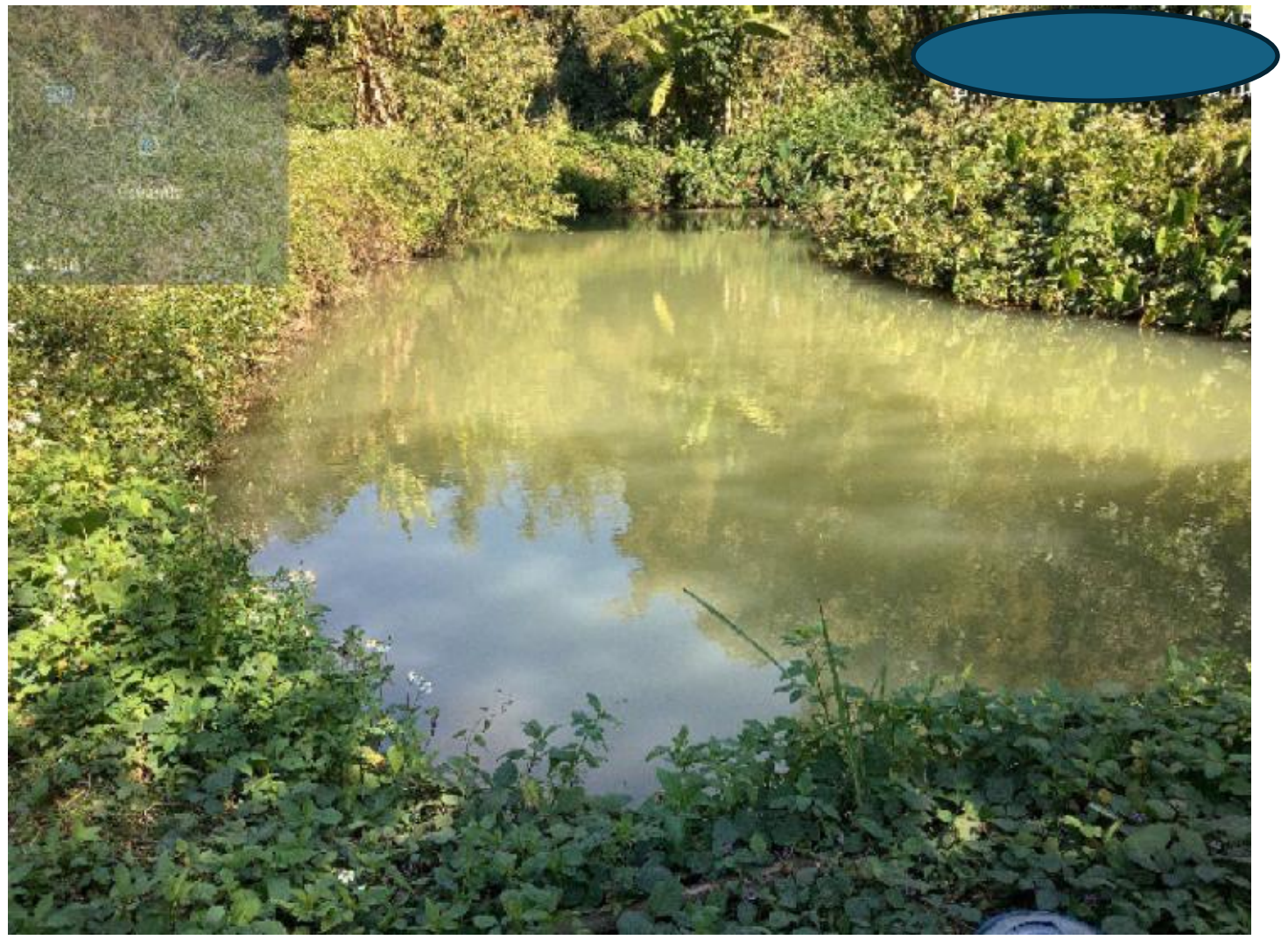	There is the forest behind this housa and the hunter sometimes he hunts some small animal like squirrels and chipmunks as well as gather bamboo shoot and rattan stem here in this wood. He said there was once he found a boar, of course he killed it.

Forest Beliefs	Like if you're going to sleep near a big tree or near a mineral lick, they'll tell you to be cautious and to ask for permission first. Before sleeping there, cutting trees, doing anything—you need to speak to the spirits first.<br>[…] If I go with the villagers, they take care of all of that. I wouldn't dare go alone. […] When I go with them, they tell me what to do. I really wouldn't go alone. (Man, 43)<br><br>They had to do a ritual called khor khama—to ask forgiveness before entering the forest. They were afraid of the forest spirits, the land spirits. People believed the forest had its own guardians. […] Before going into the forest, they'd set up a little shelter, cook some rice, and leave offerings at the entrance—rice, water, liquor. Sometimes even a chicken, to offer to the forest and mountain spirits before anything else.<br>[…] Especially those who still hold on to the old beliefs or still feel afraid. It might not be exactly like it was back then, but they still do it. They still place rice, still offer prayers, still talk to the forest spirits—saying things like, "Please allow us to enter. We	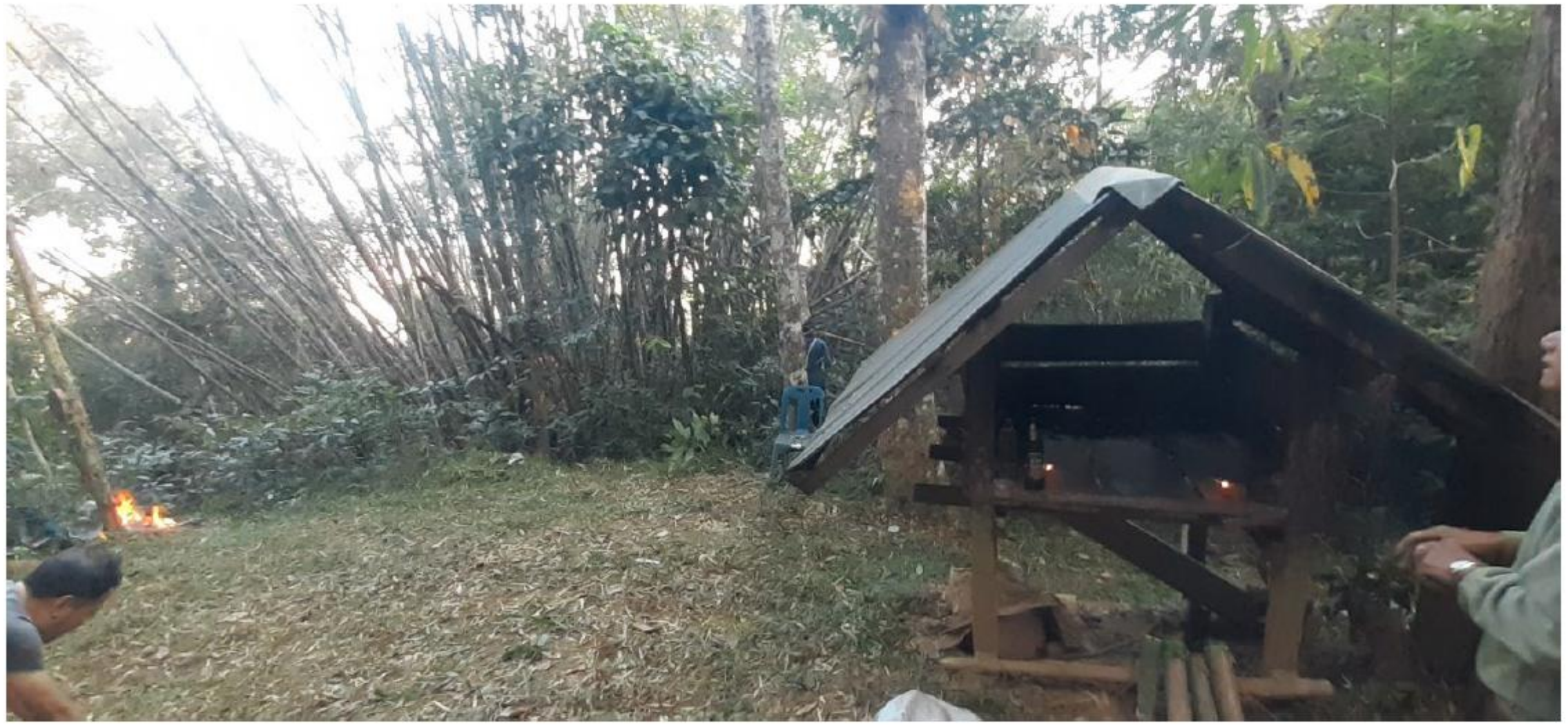<br>2) Ceremonial Area<br>Activate W<br>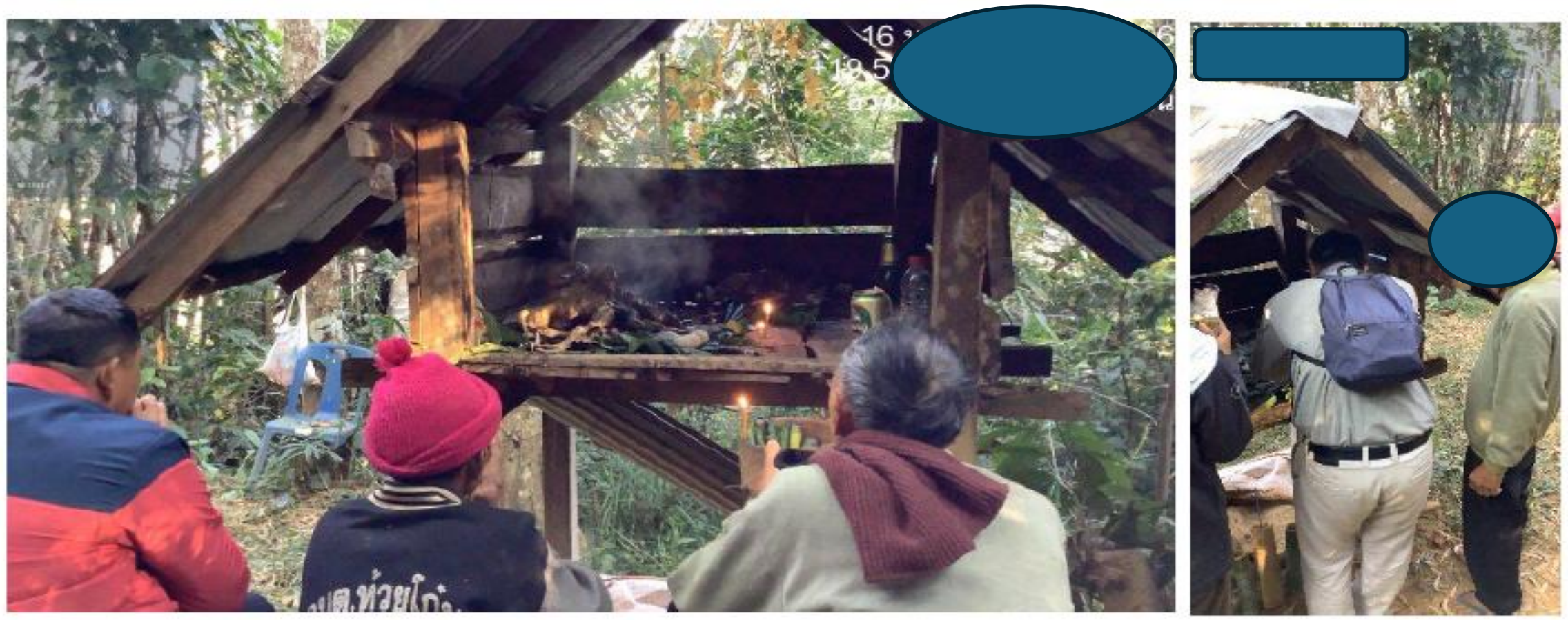<br>4) The Ritual	This area is the wide flat space in the center of the sacred forest which villagers from Lua Ethnic use it to do the ritual called 'Dok Dam Dok Daeng' means black and red flower and the shrines is built as the house of sacred spirits of the forest. The main purpose of ceremony and ritual is to praise and thank the forest gods, spirits and the wisp, who give villagers prosperity after harvesting or receiving success from taking an oath. The head of sorcerer led the ritual by

come with no harm." Something along those lines. […] If you don't ask for permission and something happens, it's really easy to get possessed—especially by the spirits that guard the sacred forest. They're strong. […] Sometimes they appear as a flash of light, kind of like a wisp—quiet and flickering. They're there guarding the forest. They don't like people wandering around without respect. […] There are quite a few. Traditionally, before we go into the forest, we first pay respects to "Mae Thoranee"—the Earth Mother. We ask forgiveness before stepping onto the land. […] Mae Thoranee is the guardian of all the earth. If you don't ask her forgiveness first, some people who go into the forest might get sick, or even get lost and not find their way back. […] We live with the spirits, with the land guardians. We need to show them respect, otherwise they won't protect us. […] We're staying on her land, so we have to call on her, ask her to protect us. Back in the day, we didn't even have cooking gear—no pots or pans—but we still had to make rice, make some food, and offer it to her. […]

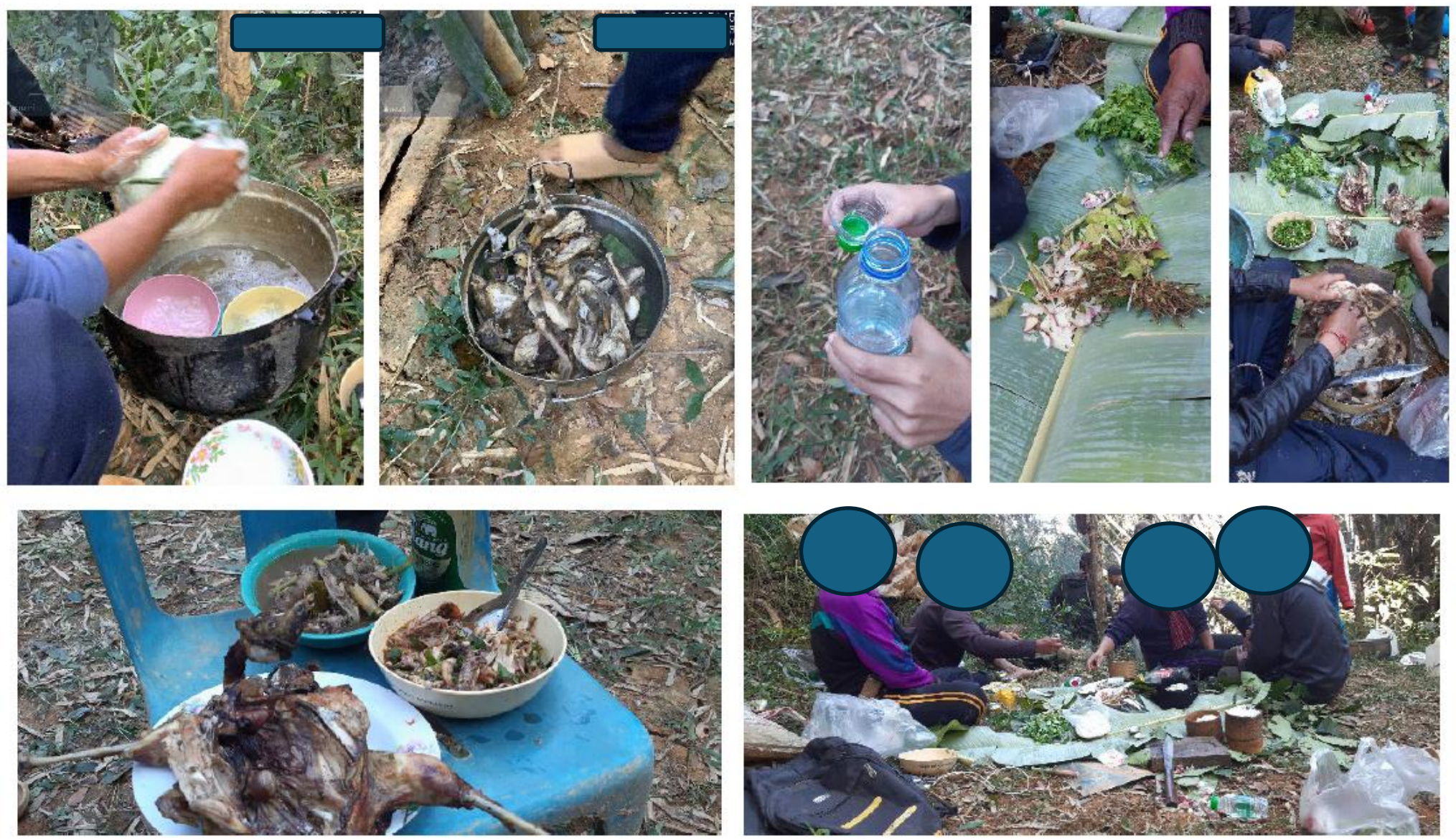

5) Time for Human's Feast

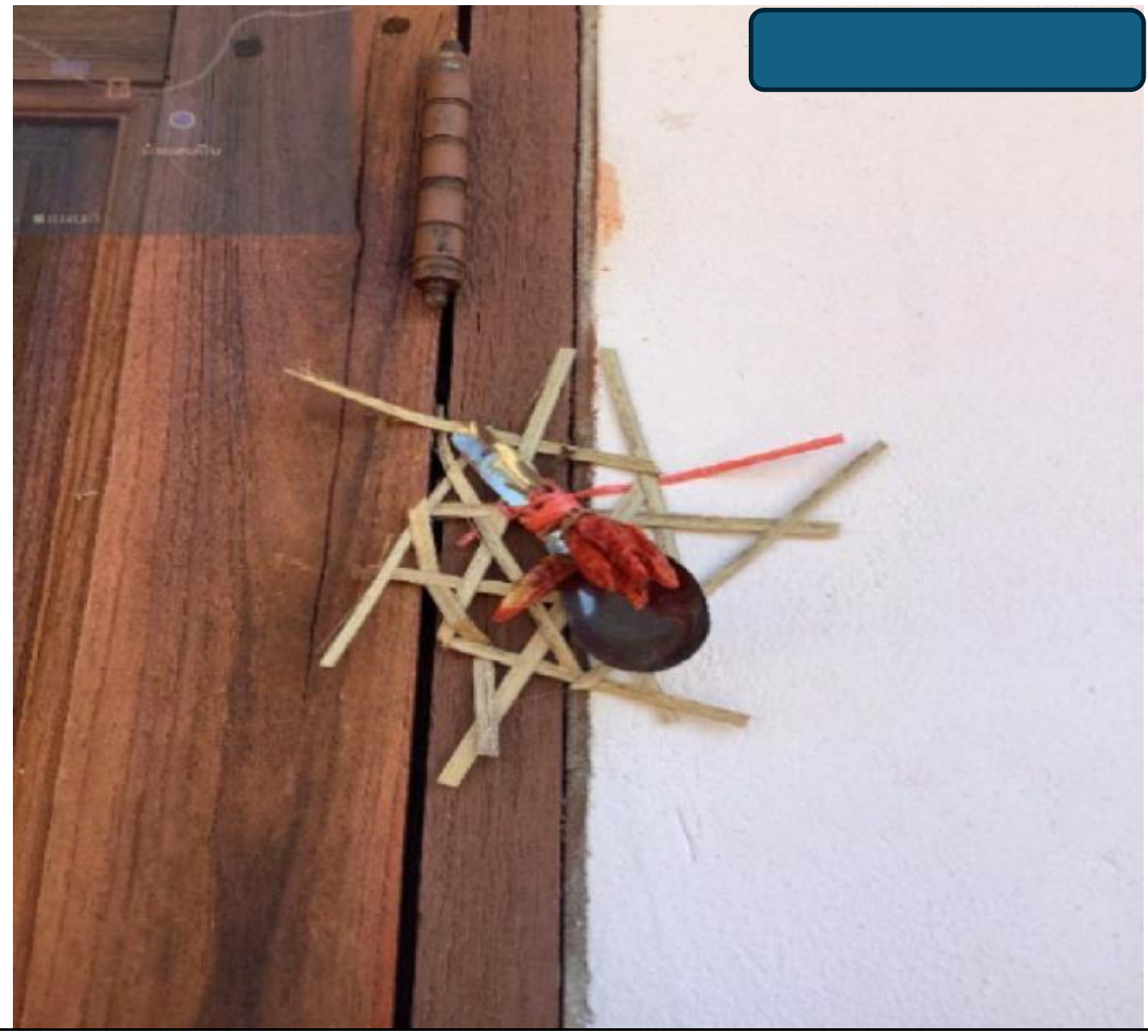

Ta Laew is the symbol of Lua's belief which is usually hanged at the front door of house, the entrance of village and the sacred forest. Villagers believe the amulet can expel the evil spirit and protect them. For the scared forest and community forest, the amulet is very effective to be used as the sign of restricted area.

placing medium rare chickens in the shrine and his disciples help him to spell ancient Lua words after the ending of spelling, all participants needed to tear chicken meat and place on the leave inside the house in order to make the respectful for the gods and wisps. Herbs, wild vegetables, and simple seasoning like salt, MSG and dried chili flakes were prepared and rest them on banana leaf. Not so long, variety of alcohols and more were distributed among them and guests must drink as

	The first thing is to never cause suffering or disturb the spirits. You have to call them, invite them first. Like when staying in the forest, you have to tell them how many people are in your group, where you'll be staying, how long you'll be there, who you are. You need to say it all. […] Let's say there are four or five of you—you have to name everyone. Otherwise, the forest spirits might misunderstand and think someone's sneaking in, hahaha. (Man, 63, Sorcerer)	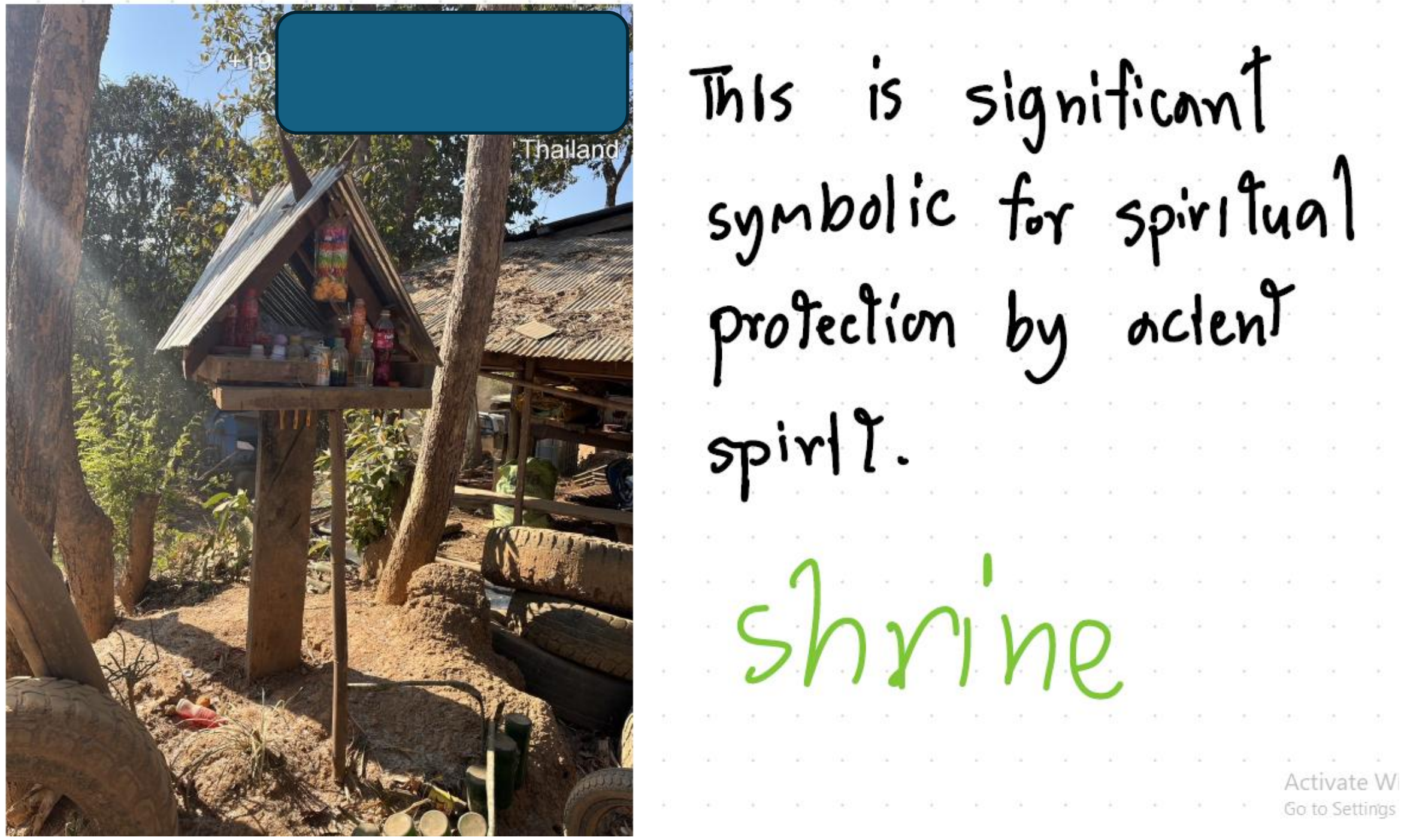<br>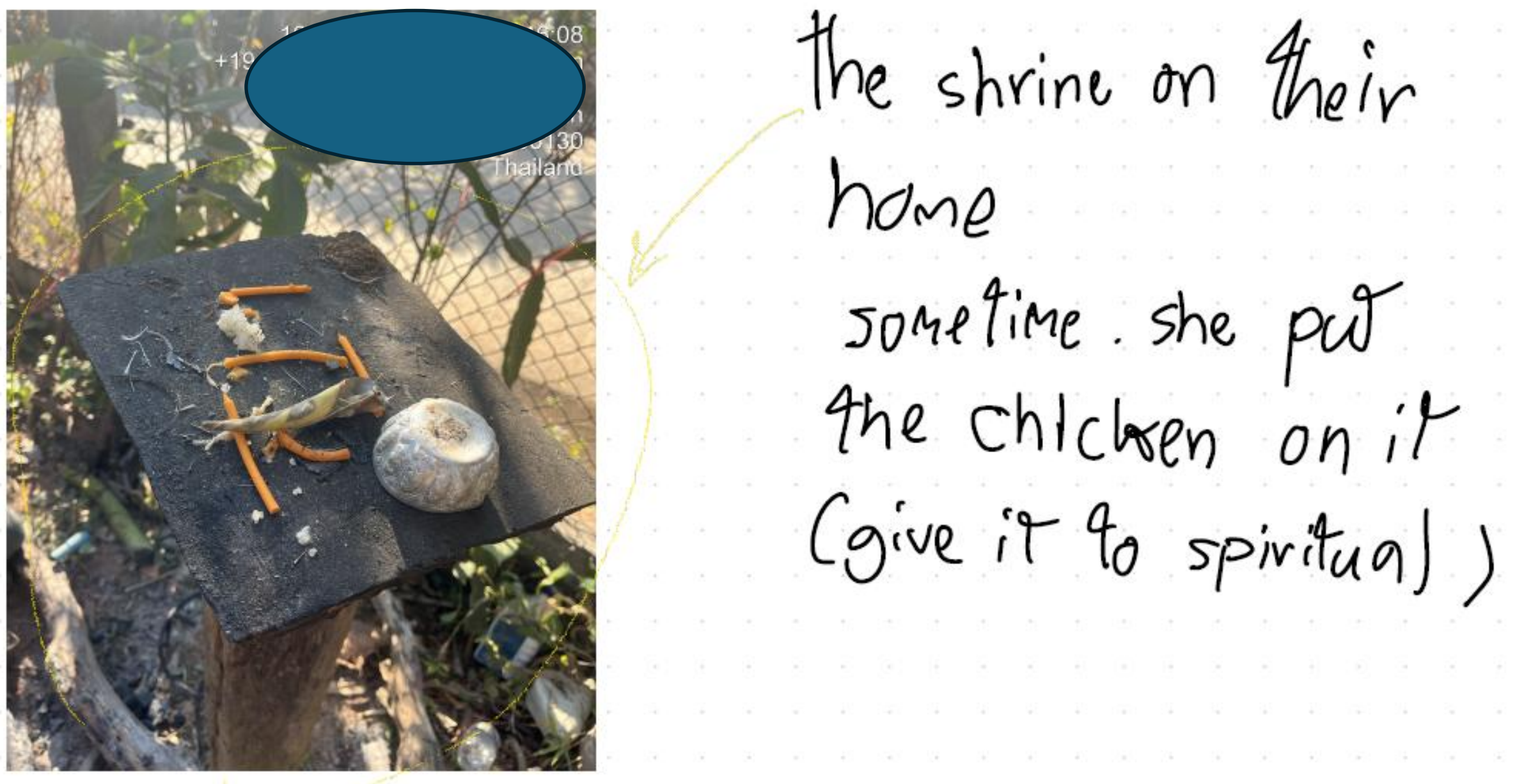		well and then chickens were brought to cook as simple recipes

Land traditions	We use the grain. There are four grains of milled rice and four grains of paddy rice. There's also a chant. After chanting, we place them down and call upon *Mae Nang Thoranee* (Mother Earth). We use a wooden stick to make a hole, place the rice grains inside, and cover it with seven leaves. Then, we cover it with soil so the leaves are not blown away. After two days, we have to check. If the grains are still there, it means we can cultivate that land. But if they are gone, it means the spirits don't allow us to use it, so we move elsewhere. For example, in my family, we practice rotated cultivation. Some people misunderstand this and think we destroy forests, but it's not like that. These lands have belonged to our ancestors for generations. (Man, 42)		
Traditional healing plants	For severe cases, people ask for rituals to remove poison, remove misfortune, or pray for help from the good spirits. Sometimes they also call the spirit (or soul) back—because it's believed that when someone is seriously ill, their duang (life force or soul) slips away into the forest, and has to be called back. […]It's like the person's inner strength is gone.		These herbs he grows for using in the ritual such as expelling the evil spirts, exorcism and healing

	Without their duang, they lose all energy. That’s why we ask the good spirits to help call it back. Sometimes we use the sound of gongs or flutes to guide the soul back into the body.<br>[…] For example, fa talai jone has been used for generations—as fever medicine, as an antidote, and even to ward off spirits. [hahaha] The old folks used it as both medicine and protective charm.<br>[…] It’s an herb used to stop bleeding. If someone gets cut with a knife or has a tear in the skin, you pound the leaves and apply it right away—it stops bleeding for sure. I’ve tried it myself. Got cut while chopping wood in the forest. Crushed the leaves, applied them—and just like that, the bleeding stopped. It is risky, though. If the wound is deep or not clean, and you use it the wrong way, some healers might say you’ve disrespected the spirits—like you performed the ritual incorrectly. […] If someone uses them carelessly, and the wound doesn’t heal or the condition worsens, some spirit healers won’t continue treating them. They’ll say “the offering was rejected”—and the person needs to make amends or find someone to	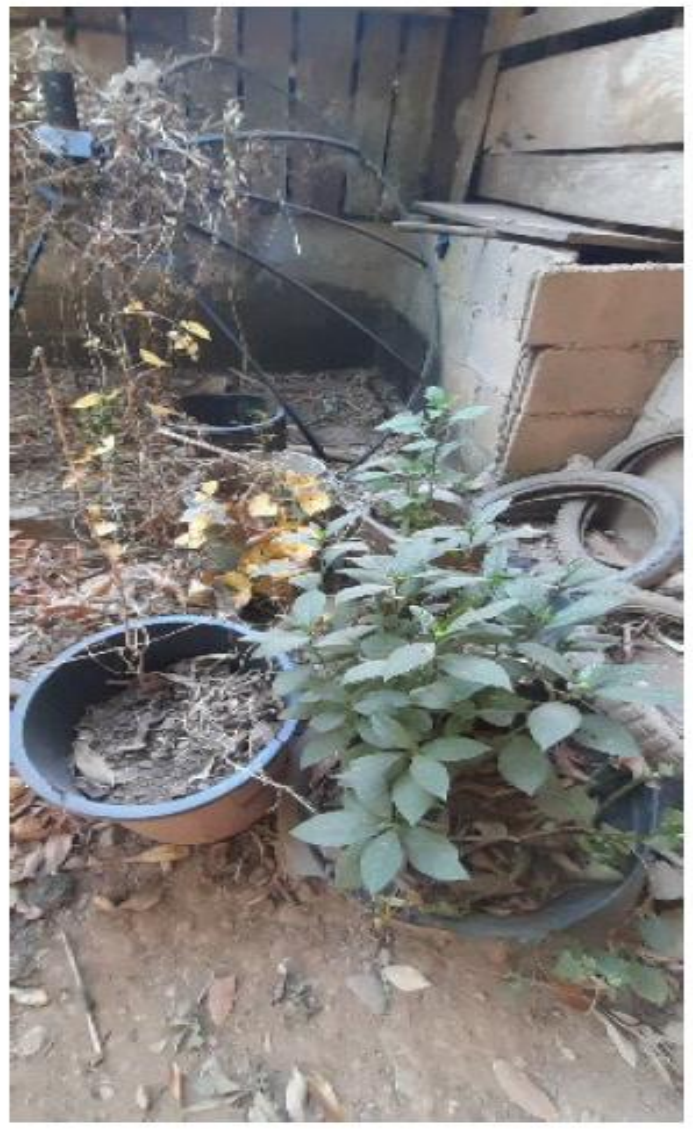 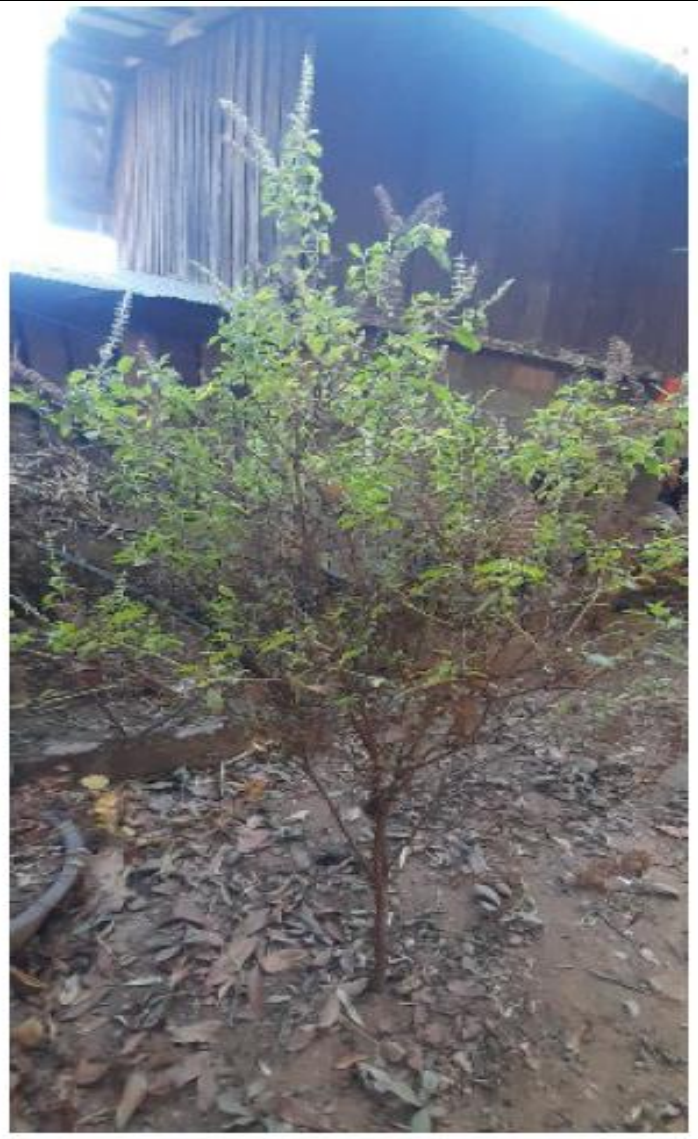 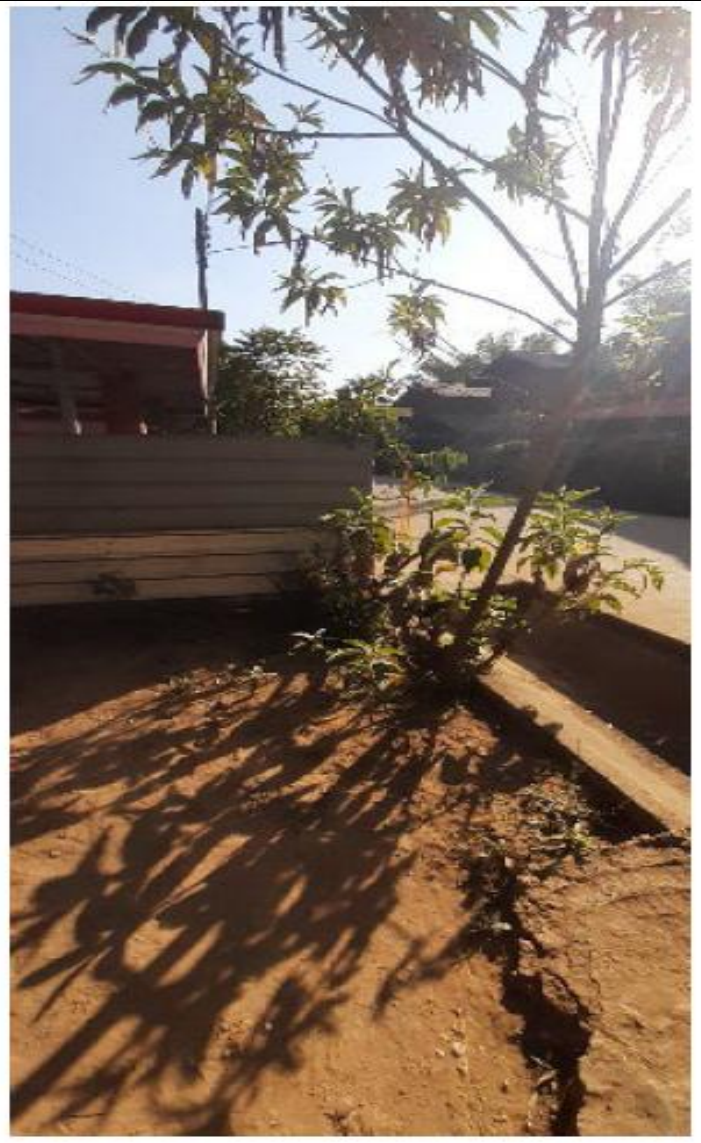<br>**4) Herbs for Mystical Practices**<br>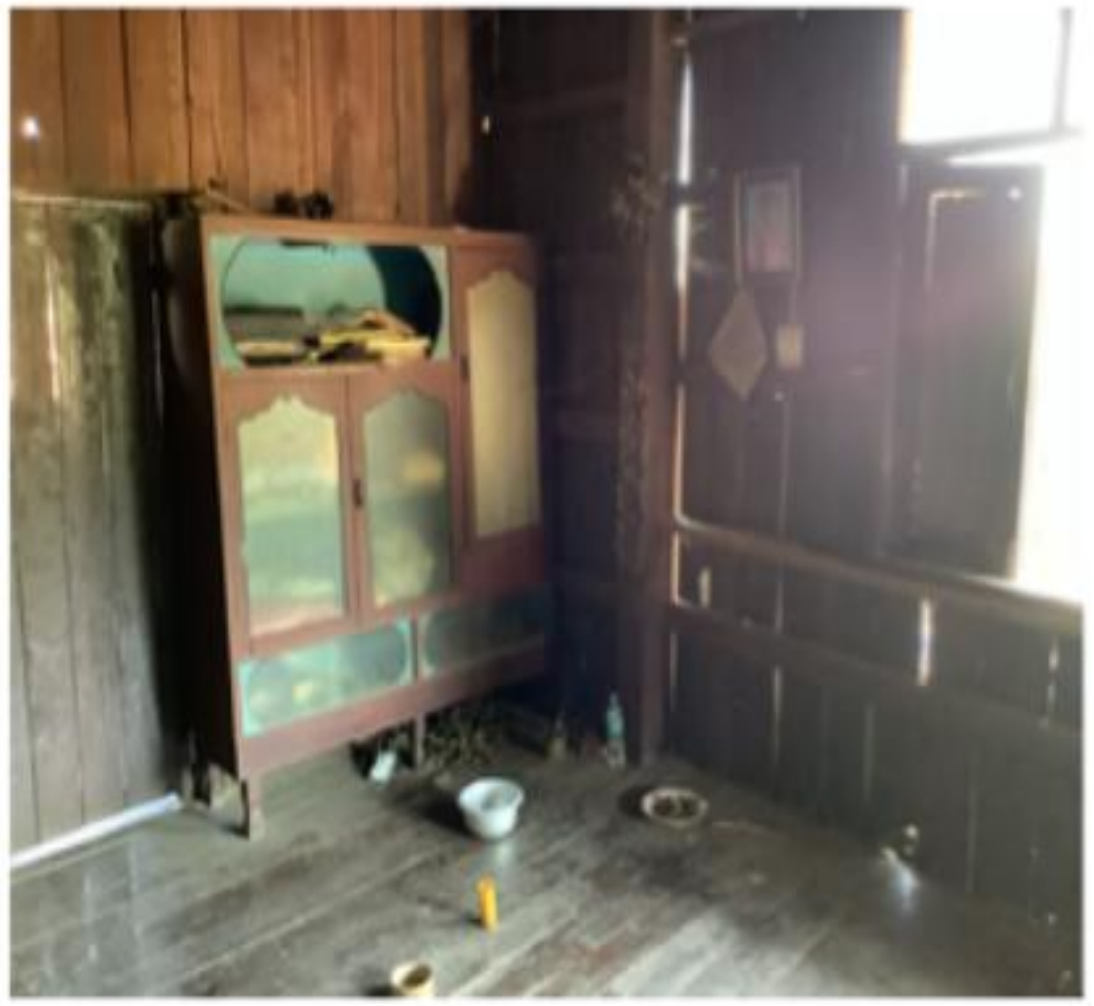<br>The place where people in the village come to do wishing ritual which is led by him (socerer). This place is inside his house (2nd floor). From what I observed, the 2nd floor only be used for this purpose.	

	perform a ritual first. […] Bai nad (Blumea balsamifera) is used too. It helps relieve bloating, indigestion, and body aches. Some people use it to compress and treat wounds. Others wrap it with other herbs, heat it up, and use it for hot compresses—it's great for muscle relaxation. It's also used to ward off spirits. […] They're not just for healing—each one is believed to have a spirit within. If you pick them without asking permission, or without respect, they won't work. The spirit of the herb won't help you. […] Before picking, we speak to the plant, saying we're taking it to heal someone, not to harm. And after using it, we bury the leftover leaves in the soil or place them back under the original tree—as a way of returning it. […] Som poi is considered a sacred herb too. You burn it first, to release its fragrance, then add it to holy water. The resulting water is used to wash away misfortune or negative energy. […] we do know that each has its own role. Som poi is used to cleanse bad energy, wash away curses or misfortune. Bai nad is more for protection—used in rituals to guard against illness, block bad spirits, or call back the	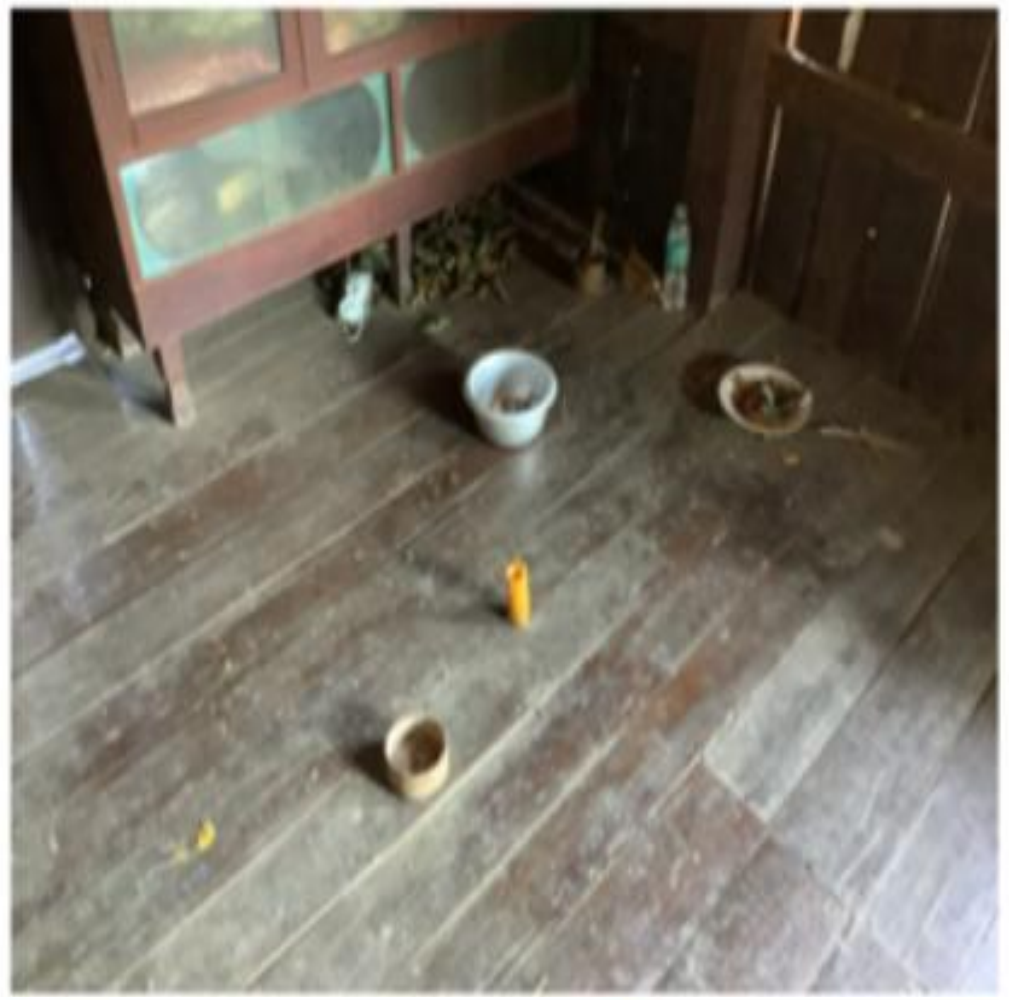<br>The place where they sit while doing the ritual with a big candle.<br>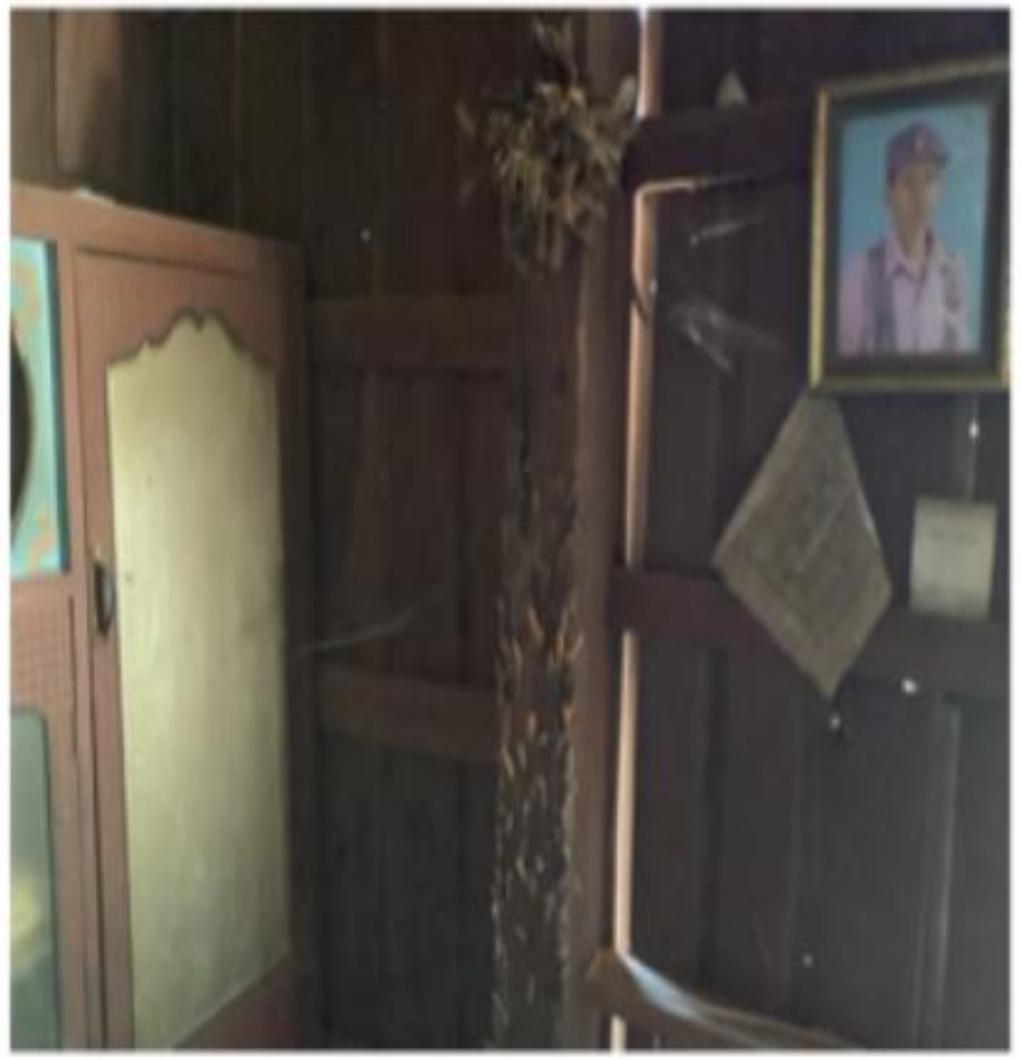<br>All candles brought to the ritual are lighten up and stuck to the pole.	

	soul. […] The natural scent is believed to activate the power of the sacred. Good spirits can sense it through the smell, while evil spirits are driven away. […] these sacred plants carry their own power. The old folks knew this. They didn't use them carelessly. Every time you pick a leaf, you must ask permission. […] Like our parents used to say—they didn't always know why it worked, but they did it anyway. And when the spirits reacted, they believed.[…] Phi ka and phi pob fear strong flavors, intense smells, and sharp shapes. So people use chilies, pointed leaves, and aromatic herbs to create a spiritual shield for their homes. (Man,63, Sorcerer)		
Insufficient infrastructure at the market	They [authorities] don't pay much attention to this area because it's an open-air market. If it were a space managed by the subdistrict, it would have concrete flooring and drainage. […] but this area is under the customs department, and they don't have the budget. The subdistrict office here generates revenue from the market, but as long as the land belongs to the customs		

	department, we can't make any significant improvements like paving the area or installing drainage. We can only make it functional because this area is under the customs department, but there are budget limitations and other constraints. There are many factors involved. (Women, 47, Market Organizer)		
Waste separation and disposal at the market	The Thai vendors collect their trash and put it in the garbage bins near the streetlights. Then, a worker with a tricycle will come and collect it, put it in a bag, and take it to the landfill. The subdistrict has dug a landfill pit for this purpose. […] There's usually not much animal waste because every part of the animal is valuable. […] Wastes are from some rotten fruits or vegetables. […] They butcher the animals at home, so there's almost nothing left. Everything is valuable. (Women, 47, Market Organizer)		

	There are deer blood, civet blood, you know, a lot of things. They put everything into the bin, and it starts to smell. So, I just cover my nose with a mask and wear gloves. They throw everything together, and I can't separate it. Each type of bin is provided for garbage separation, but they don't separate it. Oh. It's even more difficult during the rainy season. Oh, it gets messy. There's the vehicle for garbage collecting, and they drive while collecting it. It gets all messy. Even with an apron, even wearing boots, and with cloth, it still gets messy. Everywhere, the whole border—down below, up above. Both the waste, the blood, and everything. Oh, it's everything. […] they use diesel oil to burn it. There's also use oil from the subdistrict administrative organization. (Man, 62, Market cleaner)			

	The bins belong to the subdistrict administrative organization. They come and throw their waste in those points. Up there, sometimes, there is blood everywhere. They don't separate it. We have bins for separating, but they don't separate it. They are not well educated… […] the waste is a big issue, along with the water. Those two are the main problems. Everything else is not as bad. […] They don't separate it. They throw everything together, and it smells bad. They [subdistrict administrative organization] promote it, but people don't separate it. One person can't manage it. There are 30 bins sitting there. I have to drag them into a pickup truck. (Man, 62, Market cleaner)<br><br>It's a pit. […] 20x20 meters in size, and 5 meters deep. […] If they cover it up again, they will dig it up again. We have to cover this pit, then dig another one. […] If it's full, it will be covered. […] The burnt garbage and soil in the		

	first pit will be dug out to cover another pit. Dig from that to cover this. Move in and move out like that. […] It will be burnt every Sunday. […] Everything. All sorts of things. (Man, 62, Market cleaner)		
Protection gear of market cleaner	They put everything into the bin, and it starts to smell. So, I just cover my nose with a mask and wear gloves. [They also provide] Boots. […] They also give us bathroom cleaner, 3 bottles per week. […] and also 2 mops per year.<br><br>(Man, 62, Market cleaner)		

Table 5. Human-Communication interface

Code	Quote.
Trust in community leaders and VHVs	We're lucky that the villagers trust and listen to their leaders. (Man, 56, Community leader)<br><br>If someone's in a leadership position, people listen. I always tell the villagers—trust your leaders. We live with the forest, and the forest lives with us. People and the forest need to support each other. […] I think it's because I do what I say. If I say something is banned, and someone disobeys, I take action. I don't make empty threats. At the same time, if a villager is in trouble, I listen and help. […] The villagers trust me because I actually help—not just talk. If I didn't really help, I wouldn't have lasted 30 years. These days, people file complaints all the time. Some village heads are constantly being reported. (Man, 56, Community leader)<br><br>When villagers have problems, they come to consult and ask me for help. They cooperate with us well. It's like they trust us and come to us for advice. They can come to me with any problems. When I talk to them, it makes them feel better. I get along with people easily. (Women, 50, Village Head)<br><br>They trust it because I always update them whenever there is an outbreak in the village. If it's nearby, I go and tell them in person. But most of the time, I use the Line group to spread the news. (Women, 49, Head of VHVs)
Adapted language	I always prepare the information before going to work in those villages. I speak in the northern dialect. If people from the city go there, it might be a bit difficult for them because they're concerned about scams. […] If we speak the same language, it's easier to understand. There hasn't been any issue. I just inform the village chief, and there's nothing to worry about. (Women, 46, office worker in forest office)
Mode of communication	The district office sends announcements via the public relations LINE group for the villagers. Most of the announcements during the rainy season focus on mosquito larvae—advising people to keep their homes clean and eliminate standing water to prevent mosquito

	breeding. These messages are usually sent out twice a month. (Man, 52, Head of village)

Table 6. Animal-Environment interface

Code	Quote	Image	SW Note
Free roaming farm animals	I have water buffalo. I let them roam in a designated area in the forest. […] In this area, everyone has their own designated space. We use barbed wire to mark the boundaries. The cattle can roam freely within these areas and find water from the streams in the forest. […] It's an area of national park but we can raise them there. […]. If you're raising cattle or buffalo, you need to provide them with water. We have to find a wide area, build a fence, and let them have access to water. There's plenty of natural grass for them to eat. (Man, 41)	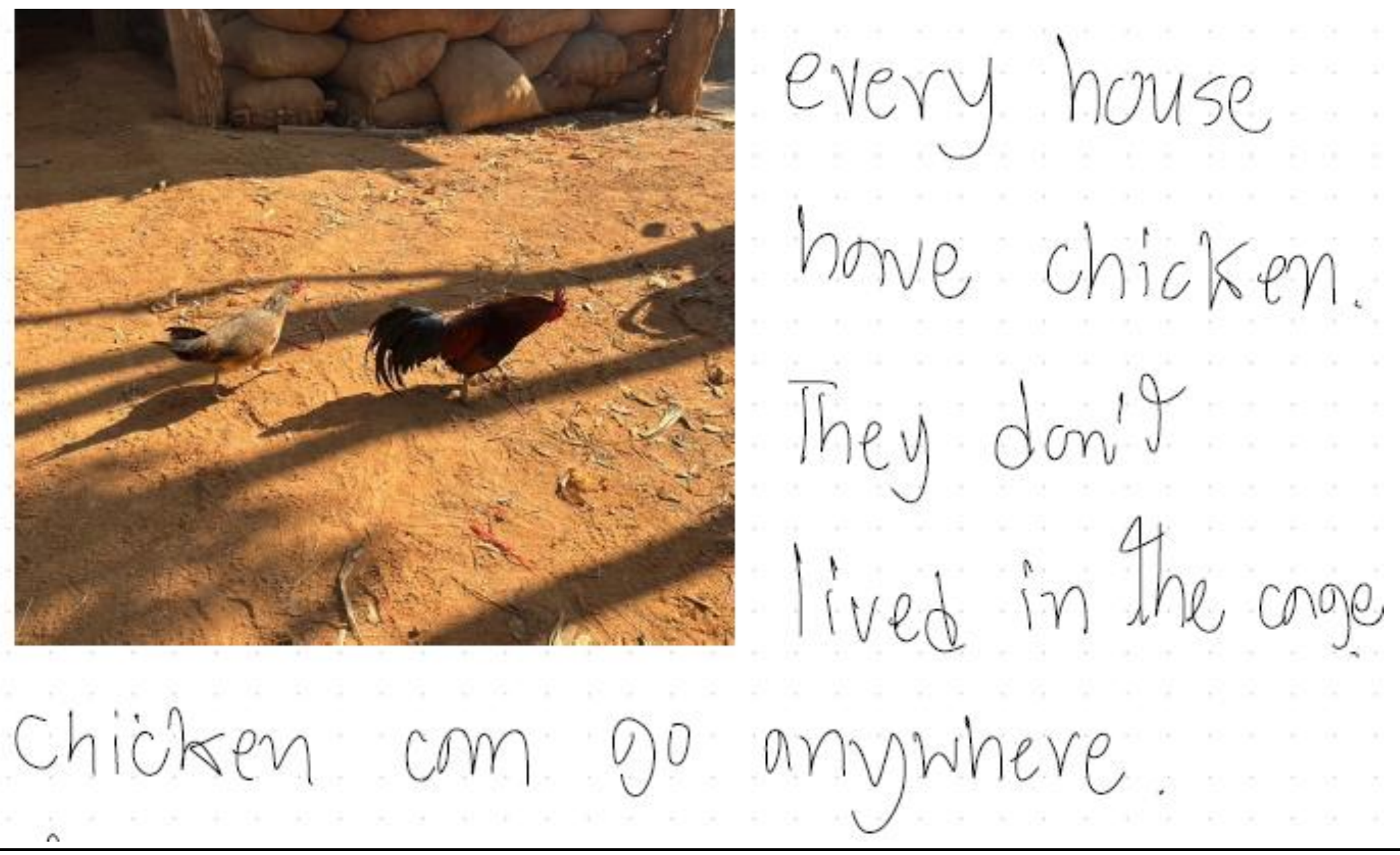	
Contamination of other products with wildmeat in the market	They hide the animals in pine wood or in vegetable bags. They have their ways of hiding things. (Women, 47, Market Organizer)<br><br>Normally, we hide them. I put them in the bag. Sometimes I bring vegetables too, and I put them in the bag and put the vegetables on top. […]		We saw that there were vegetables packed for sale in foam boxes. But hidden under the vegetables was bushmeat. - RA 3

	they'll see vegetables and other things. (Women, 29, Market vendor)		
Animal waste in market environment			During market closing times, all Lao vendors who sell bushmeat wash the blood in their foam boxes with water and dump onto the ground at their selling area. -RA 3

Water and animal waste interface	This is the water pump that pumps water up to the tank above. […] this is for the border trade market. So that the vendors who rent space here can use the water. […] It comes from below. […] And this is where the water goes into the high tank, the sedimentation tank, and then the alum tank. […] the water is pumped up to a high tank and then released. […] If you need more water, you come here. […] there are faucets. Sometimes they wash their hands there. (Women, 47, Market Organizer)		Water used for tap water for people living in border areas is pumped from the reservoir lower than the landfill and market. Market cleaners said that during the rainy season, the market area where [foreign] vendors sell their products is in terrible condition due to the stagnant rainwater. From the environment of the market, it can be seen that the market is not far from the garbage dump and the reservoir used for

				making tap water. The market is at a higher level than the garbage dump and the reservoir. From observing the market, it was found that during the closing time, all [foreign] vendors who sell bushmeat will wash the blood in their foam boxes with water and dump it onto the ground at their stalls. Therefore, during the rainy season, the water flowing from the market and the garbage dump will flow into the reservoir that

			used for making tap water .- RA 3

Table 7. Animal- Animal interface

Code	Quote	Image	SW Note
Transmission of disease in farm animals	There were two cattle that weren't properly cared for, so the disease spread to other people's cattle. Eventually, the infected cattle died, and they had to be buried. […] We had to buy vaccines and inject them into our cattle. […] and it spread to nearby cattle. Some died, so The Department of Livestock Development provided medicine to sprinkle over the dead cattle. […] and our cattle didn't get sick because we took preventive measures. […] The owner didn't take good care of them. […] the disease spread to others, so people quickly vaccinated their cattle, and the outbreak was contained. (Women, 43)<br><br>But there was one year when there was an outbreak of avian flu. Many chickens died, but the ones I raised were fine. It was called "Ha Kai" in our local dialect, which means "Chicken Plague." […] there are many chickens died from that. […] My chickens were fine, even though I was worried. My farm is quite isolated, so the		

	disease didn't reach us. But this disease spreads very quickly. […] Sometimes it starts in neighboring villages. People might bring in chickens from other places, and that's how the disease spreads. […] Before they died, the chickens had a runny nose. It was probably a type of flu. […] just like when humans catch a cold, it spreads easily. It's like a bacteria. (Man, 41)		
Rabies in dogs	We had outbreaks [rabies] too, especially during the hot season. That's when rabid dogs would start appearing. […] But the people who were bitten went to see the doctor right away and got vaccinated in time, so no one was seriously harmed. […] But most of the time, the outbreak was just among the dogs themselves. They'd bite each other. People rarely got bitten. […] We didn't really do much. We just left it. Eventually, the infected ones would die off. […] Yes, the ones that were seriously infected would die, and in the end, there would be none left. […], if a dog was badly infected, it would die, and the owner would dig a hole and bury it. […] It was simple. Just like digging a hole in the field—	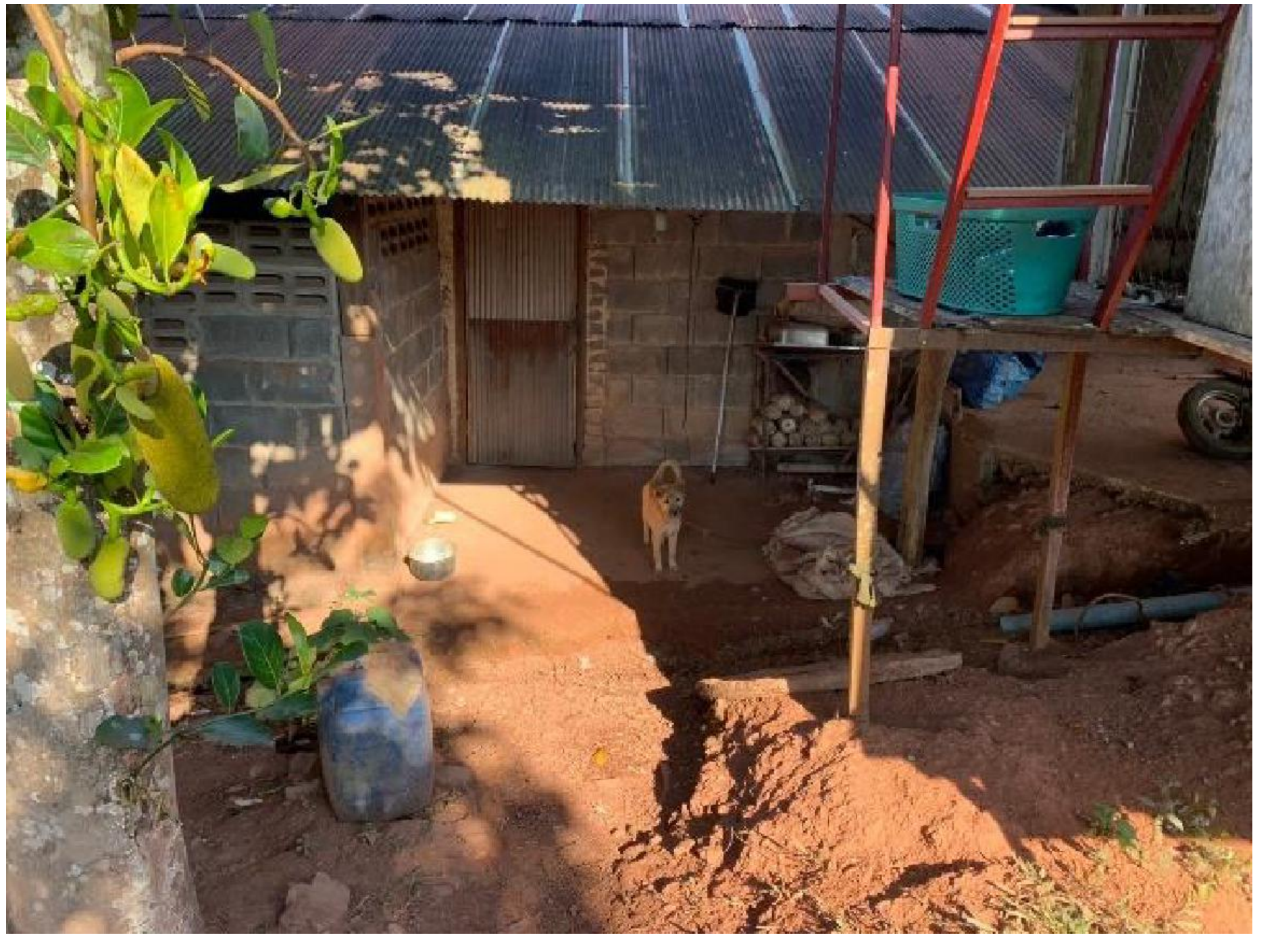	

	dig it and toss the dog in. No cremation. (Man, 56)		
Live animal interactions in market			The catfish flailed and were pressed against one another, many with visible wounds, swimming in murky water that reeked of a thick, fishy slime. (RA 7)<br><br>I also observed the cages in which the vendors kept these animals [mole rats], which were old and crowded enclosures that forced the animals into cramped conditions. (RA 7)
